# Computationally Guided Design, Synthesis, and Evaluation of Novel Non-Hydroxamic Histone Deacetylase Inhibitors, Based on *N*-Trifluoroacetamide as a Zinc-Binding Group, Against Breast Cancer

**DOI:** 10.3390/ph18030351

**Published:** 2025-02-28

**Authors:** Gerardo Morales-Herrejón, Juan Benjamín García-Vázquez, Cynthia Fernández-Pomares, Norbert Bakalara, José Correa-Basurto, Humberto L. Mendoza-Figueroa

**Affiliations:** 1 Laboratorio de Diseño y Desarrollo de Nuevos Fármacos e Innovación Biotecnológica, Escuela Superior de Medicina del Instituto Politécnico Nacional (ESM-IPN), Plan de San Luis y Salvador Díaz Mirón S/N, Casco de Santo Tomás, Ciudad de México 11340, Mexico; gerardo_mor@outlook.com (G.M.-H.); benjagv_5202@hotmail.com (J.B.G.-V.); cyfernandez@uv.mx (C.F.-P.); 2 Investigadoras e Investigadores por México CONAHCyT-Sección de Estudios de Posgrado e Investigación de la Escuela Superior de Medicina, Instituto Politécnico Nacional, Plan de San Luis y Salvador Díaz Mirón S/N, Casco de Santo Tomás, Ciudad de México 11340, Mexico; 3 University Bordeaux, CNRS, Bordeaux INP-ENSTBB, CBMN, UMR 5248, F-33600 Pessac, France; norbert.bakalara@enstbb.fr

**Keywords:** histone deacetylases, HDAC inhibitors, hydroxamic acid, zinc binding group, molecular docking, ADMET, breast cancer

## Abstract

**Background**: Histone deacetylases (HDACs) are enzymes that deacetylate histone proteins, impacting the transcriptional repression and activation of cancer-associated genes such as P53 and Ras. The overexpression of HDACs in breast cancer (BC) underscores their significance as therapeutic targets for modulating gene expression through epigenetic regulation. **Methods**: In this study, a novel series of SAHA (suberoylanilide hydroxamic acid) analogs were designed using an in silico ligand-based strategy. These analogs were then synthesized and evaluated for their HDAC-inhibitory capacity as well as their antiproliferative capacity on breast cancer cells. These compounds retained an aliphatic LINKER, mimicking the natural substrate acetyl-lysine, while differing from the hydroxamic fragment present in SAHA. **Results**: The synthesized compounds exhibited HDAC inhibitory activity, suggesting potential for binding to these pharmacological targets. Compounds **5b**, **6a**, and **6b** were identified as promising candidates in the evaluation on breast cancer cell lines MCF-7 and MDA-MB-231 at 72 h. Specifically, compound **6b**, which contains an *N*-trifluoroacetyl group as a zinc-binding group (ZBG), demonstrated an IC_50_ of 76.7 µM in the MDA-MB-231 cell line and 45.7 µM in the MCF-7 cell line. In the non-tumorigenic cell line, the compound exhibited an IC_50_ of 154.6 µM. Conversely, SAHA exhibited an almost negligible safety margin with regard to its cytotoxic activity when compared to breast cancer cells and healthy cells (MCF-10A). This observation underscores the elevated toxicity exhibited by hydroxamic acid-derived molecules. **Conclusions**: The bioisosteric modification of ZBG by *N*-trifluoroacetyl in **6a** and **6b** demonstrated favorable cytotoxic activity, exhibiting a higher safety margin. This study underscores the challenge of identifying novel ZBGs to replace hydroxamic acid in the development of HDAC inhibitors, with the objective of enhancing their physicochemical and toxicological profile for utilization in BC treatment.

## 1. Introduction

Cancer is a group of diseases where abnormal cells grow uncontrollably and spread throughout the body, driven by genetic alterations in oncogenes like *Ras* and inactivation of tumor suppressor genes like P53 [1,2,3]. It has been reported that epigenetic mechanisms, such as DNA methylation and histone modifications, are intimately involved in cancer, with histone acetylation playing a crucial role in its development and progression [4]. Histone deacetylases (HDACs) are enzymes that deacetylate histone and non-histone proteins, promoting transcriptional repression and the silencing of cancer suppressor genes [5,6]. Currently, eighteen isoforms of HDACs have been identified in humans, classified into two families according to their catalytic mechanism: Zn^2+^-dependent metalloenzymes and those that use nicotinamide adenine dinucleotide (NAD+) as a cofactor [7]. HDACs are also classified into four classes according to their homology and intra-cellular localization: class I (HDACs 1, 2, 3 and 8), class II (HDACs 4, 5, 6, 7, 9 and 10), class III (Sirt1–Sirt7), and class IV (HDAC11) [7,8,9].

Breast cancer (BC), the most common type among women and the leading cause of cancer-related death, develops in the breast due to the uncontrolled proliferation of glandular epithelial cells [10,11]. It has been shown that cancer cells overexpress HDACs in certain types of cancer such as BC [9,12,13,14]. HDACs are involved in key cellular processes such as transcription regulation, apoptosis, and DNA damage repair [15].

**Figure 1:**
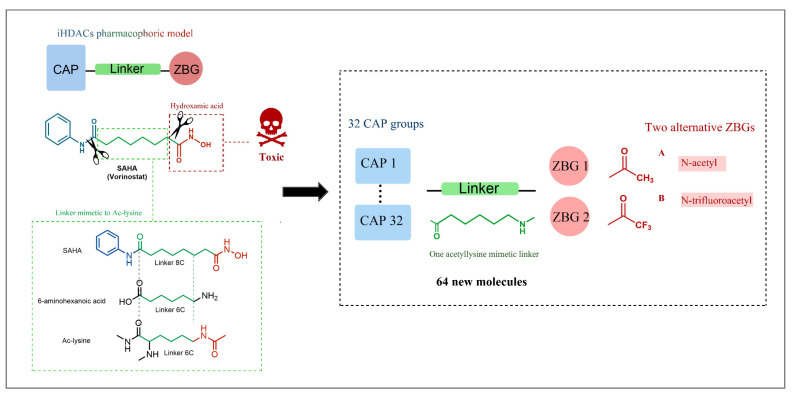
Design of *N*-acetyl (A) and *N*-trifluoroacetyl (B) derivatives based on the pharmacophore model of HDACi (CAP-Linker-ZBG). The ZBG is represented in red, the LINKER chain in green (in this representation includes the carbonyl carbons and amine base as part of the linker chain), and the proposed *CAP* groups in blue.

HDAC inhibition has emerged as a promising epigenetic therapy for cancer treatment [16,17]. HDAC inhibitors (HDACis) prevent deacetylation, restoring cellular acetylation homeostasis and normalizing the expression of proteins that can reverse cancer progression [17,18]. Thanks to crystallographic information of HDACs reported in the Protein Data Bank and numerous structure-activity relationship studies (SAR) of various HDACis, a general pharmacophore model for HDACis has been developed [19,20]. This model identifies three domains: (1) a CAP group, consisting of a hydrophobic or aromatic group capable of interacting with the edge of the interaction site; (2) a zinc-binding group (ZBG), which can coordinate with the zinc ion at bottom of the cavity; and (3) a LINKER domain, which connects the CAP group to the ZBG, as depicted in Figure 1 [20].

Nowadays, four HDACs have received FDA approval for the treatment of lymphomas and multiple myelomas: Vorinostat (SAHA), Belinostat, Panobinostat, and Romidepsin. Chidamide, a non-hydroxamic inhibitor, has also been approved by the CFDA (Chinese Food and Drug Administration) for the treatment of refractory peripheral T-cell lymphoma (PTCL). However, most of these drugs contain a hydroxamic acid fragment, leading to toxic effects due to binding to other zinc-dependent enzymes and poor pharmacokinetics, causing a numerous side effects such as fatigue, nausea, and thrombocytopenia [3,4,5,6]. Given this, the toxicity of current HDACis highlights the need to develop non-hydroxamic acid inhibitors [21]. This would result in better-tolerated compounds with fewer side effects, thus improving the effectiveness of cancer treatment [22].

In response to these challenges, several alternative ZBGs have been developed to replace hydroxamic acid and improve the therapeutic profile of HDAC inhibitors. Carboxylic acids are among the most studied alternatives, offering moderate zinc affinity and reduced toxicity [23]. Benzamides, provide enhanced selectivity for specific HDAC isoforms, making them suitable for targeted therapies [24].

Furthermore, boronates have emerged as potent ZBGs due to their reversible interaction with zinc, improving the inhibitor’s stability and activity as HDACi. Other promising ZBGs include phosphonates and sulfonamides, which demonstrate strong zinc coordination, and heterocyclic groups such as imidazoles and oxadiazoles, which exploit nitrogen and oxygen atoms for efficient binding. These innovative ZBGs have shown potential to minimize off-target effects, improve pharmacokinetic properties and ultimately lead to safer and more effective HDACis. These strategies highlight the importance of exploring novel ZBGs that can maintain or enhance the therapeutic efficacy of HDACis while reducing side effects. This approach is key to developing safer and more effective compounds, particularly for cancer treatment [23,25].

In the aforementioned context, the present work focuses on the design of novel non-hydroxamic HDACis that are structurally related to the pharmacophore group of classical inhibitors [19], such as suberoylanilide hydroxamic acid (SAHA or Vorinostat), which demonstrate antiproliferative activity against BC cell lines.

## 2. Results and Discussion

### 2.1. Rational Molecular Design

Hydroxamic acids are known for their potent inhibitory activity against HDACs due to their ability to chelate the Zn^2+^ within their catalytic site. However, this binding property can contribute to adverse effects such as fatigue, gastrointestinal issues, thrombocytopenia, and cardiotoxicity. These toxicities are significant concerns in clinical settings and drive the ongoing research to develop HDACis with alternative ZBGs that may offer improved safety profiles [6,22].

In our rational design, we have employed a ligand-based strategy, which included the bioisosteric modification of the pharmacophoric scaffold of classical HDACi. Our efforts were primarily focused on the modification of the ZBG fragment. To achieve this, we considered SAHA as the reference scaffold using the phenylacetamide group as the CAP, a six-carbon alkyl LINKER, and a hydroxamic acid as ZBG. Then, to design acetyl-lysine mimetic ligands, we used a hydrophobic linker with five carbon atoms and an acetamide group as the ZBG, including several steric and electronic variations on the CAP group (Figure 1). These structural approaches were taken with the intention of promoting and favoring molecular interactions on the surface of the catalytic cavity and its neighbors’ amino acid residues, which would modulate the affinity of the inhibitors by properly orienting the LINKER and ZBG in the catalytic cavity [26].

**Figure 2:**
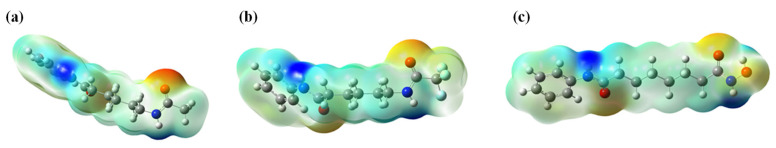
Molecular electrostatic potential diagrams of (**a**) *N*-acetyl and (**b**) *N*-trifluoroacetyl derivatives and (**c**) SAHA, calculated at theory level M062X (d,p) with a cubegen isovalue of 0.00100 (Gaussian 09 software, Gaussian Inc., Wallingford, CT, USA). Red: high electron density (negative charge, nucleophilic sites). Blue: low electron density (positive charge, electrophilic sites). Green/Yellow: neutral regions.

Although the acetamide group is undoubtedly an excellent choice as a ZBG, in our design we have incorporated a trifluoroacetamide group under three premises: (1) the fluorine atoms will facilitate the formation of favorable electronic environments to improve the F···H-N, F···H-O interactions (with catalytic amino acids) and even the F···H-C interactions can be enhanced, due to the hydrophobic environment of the cavity [27]; (2) the trifluoroacetyl group increases the electrophilic character of the carbonyl group, leading to in situ hydration in accordance with the catalytic mechanism of class I and II HDACs, resulting in a bidentate coordination to the Zn^2+^ ion through the two resulting hydroxyl groups [28,29]; (3) the trifluoroacetamide group exhibits slightly greater polarizability than acetamide (a property that has been investigated through the quantum mechanical calculation of the molecular electrostatic potential diagram, Figure 2), which will facilitate a more effective chelating and selective effect on the acidic ion Zn^2+^, in comparison to hydroxamic acid which is highly polarizable and capable of coordinating Fe^2+^, Cu^2+^, and Mg^2+^ [30]. Specifically, this bioisosteric change would result in enhanced cofactor binding selectivity, affinity, and reactivity competition with the natural substrate, with lower toxicity than hydroxamic acid.

Once the pharmacophoric requirements had been established, 6-aminohexanoic acid was selected as the structural basis for the design, with the objective of incorporating a variety of nucleophilic CAP groups and ZBG fragments with electrophilic characteristics. Accordingly, for the purposes of this study, 64 ligands with high feasibility of chemical synthesis are proposed (Table S1, Supplementary Materials), thanks to the pharmacophore and chemical versatility of aminocaproic acid.

### 2.2. Computational Design

#### 2.2.1. ADMET Prediction

ADMET properties were calculated for the 32 molecules from family **A** and 32 molecules from family **B**, yielding acceptable values in most molecules and, in many cases, values that were better than SAHA (Vorinostat). ADMET predictions for **SAHA** describe it as having good pharmacokinetic properties, favorable drug-like characteristics, and no cytochrome inhibition effects, although it presented two structural alerts from Brenk for the hydroxamic acid, indicating potential mutagenic and tumorigenic properties (Table S2). This is consistent with information reported in the literature on the mutagenic potential of Vorinostat and hydroxamate derivatives in general [3,31]. Table 1 includes the calculated ADMET values for the most promissory compounds **A6**, **A7**, **B6**, **B7**, and **SAHA**.

**Figure 3:**
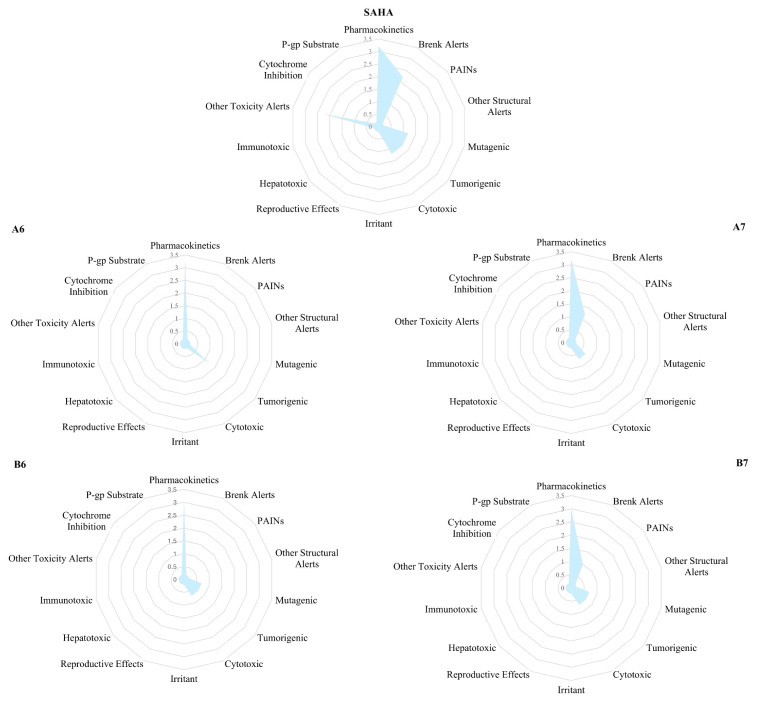
Radial diagrams of ADMET properties calculated for SAHA, **A6**, **A7**, **B6**, and **B7** (SwissAdme, ProToxII, Osiris, Molinspiration, and Microsoft Excel 365).

Structural modifications in the ZBG (*N*-acetyl and *N*-trifluoracetyl) and incorporation of 4-OH (**A6** and **B6**) and 2-OH (**A7** and **B7**) groups in CAP decreased Brenk’s and other toxicological alerts, while maintaining an adequate pharmacokinetic profile (Figure 3). Therefore, according to the in silico predictions, the structural changes positively modified the physicochemical and pharmacokinetic properties with respect to SAHA. The ability of trifluoroacetamide-based compounds to cross the blood–brain barrier highlights their favorable physicochemical properties, due to an optimal balance of lipophilicity and hydrophobicity. Substantial evidence supports the lipophilic character of the trifluoroacetylated fragment, as demonstrated in the design and synthesis of class IIa HDAC-specific radiotracers (HDACs 4, 5, 7, and 9). Specifically, 6-(di-fluoroacetamido)-1-hexanoicanilide (DFAHA), and 6-(tri-fluoroacetamido)-1-hexanoicanilide ([18F]-TFAHA) were identified as the most selective and effective radiotracers for noninvasive positron emission tomography (PET) imaging of class IIa HDAC expression activity in the brain. The translation of this radiotracer into a clinical setting holds significant promise, offering a novel approach to elucidating the epigenetic regulation orchestrated by class IIa HDACs. These proteins play a pivotal role in brain development and function, as well as in the pathophysiology of diverse diseases, including traumatic brain injury, post-traumatic stress disorders, depression, drug addiction, Alzheimer’s disease, Huntington’s disease, brain tumors, and other cerebral maladies [32]. These chemical characteristics, along with their demonstrated stability and good membrane permeability, position the target compounds as good candidates for the treatment of breast cancer. As competitive HDACi, these compounds hold significant promise for improving targeted therapy in BC, with their pharmacokinetic profile suggesting that they could be further optimized for safer and more effective treatments.

#### 2.2.2. Molecular Docking Simulations

In order to analyze binding mode, molecular interactions, and affinity energy caused by the target compounds (ligands from *N*-acetyl **A** and *N*-trifluoroacetyl **B** families) on HDAC isoforms overexpressed in BC, molecular docking studies were performed. For this purpose, we select the isoforms HDAC6 catalytic domain 2 (PDB ID: 5EEI) and HDAC8 (PDB ID: 4QA0), both co-crystallized with SAHA [33,34], and for HDAC1, PDB ID: 4BKX was used [35]. In all cases, the computational parameters were validated by the self-docking method. The alignment of the theoretical and bioactive conformations of SAHA, as well as the binding mode, demonstrated a high degree of geometric similarity (Figure S1, Supplementary Materials), particularly when the contribution of the conserved water molecules in the catalytic site of the HDACs was considered during the calculation.

**Figure 4:**
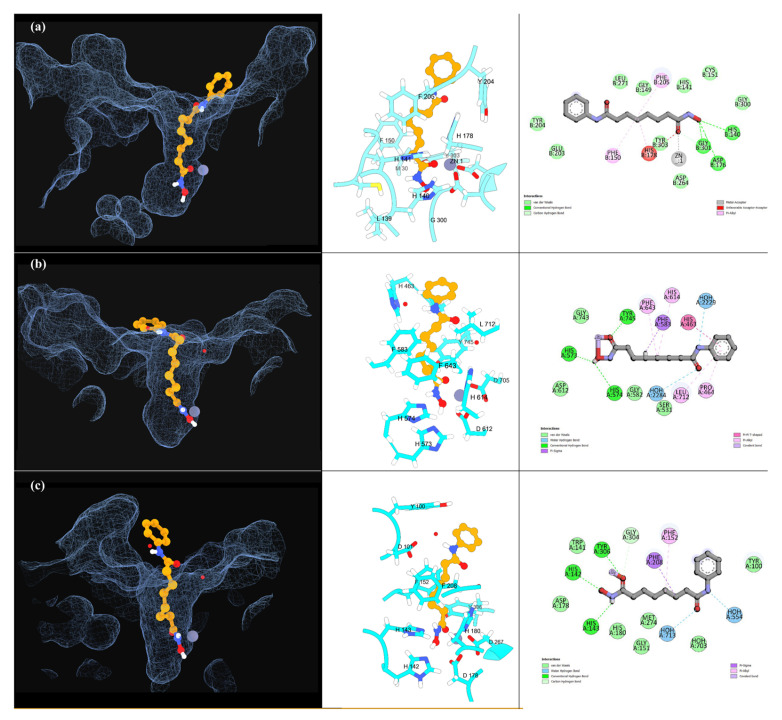
Crystallographic structures represented as a mesh for the enzyme and orange-colored balls and sticks for ligand of complex (**a**) HDAC1-SAHA (theoretical), (**b**) HDAC6-SAHA, and (**c**) HDAC8-SAHA.

Interestingly, the binding mode of SAHA maintains similar interactions, despite the few topological differences in the catalytic site cavity of each HDAC. Firstly, the aliphatic hydrocarbon chain of SAHA (linker) forms π-alkyl hydrophobic interactions with the phenylalanine residues present in all isoforms along the hydrophobic channel. Similarly, the hydroxamic acid fragment of SAHA interacts with the Zn^2+^ cofactor at the bottom of the cavity, forming a bidentate coordination interaction on HDAC6 and HDAC8 (Figure 4b,c, respectively). Nevertheless, in the theoretical HDAC1-SAHA complex (Figure 4a), a monodentate interaction was observed between the carbonyl oxygen atom of the hydroxamic acid and Zn^2+^, which underscores the necessity for hydroxamate formation to achieve the optimal chelating effect (bidentate) on the cofactor [36,37].

In addition, hydroxamic acid makes hydrogen bonds with key residues involved in the hydrolysis mechanism. These include Tyr303 in HDAC1, Tyr745 in HDAC6-CD2, and Tyr306 in HDAC8. It is known that these tyrosines are essential for the catalytic process, since they are responsible for the nucleophilic attack on the acetylcarbonyl of the natural acetyl-lysine substrate as the first part of the hydrolysis mechanism [3]. Finally, there is a cluster of catalytic histidines, which act as the acid-base throughout the hydrolysis process, including His140 and His141 in HDAC1, His573 and His574 in HDAC6-CD2, and His142 and His143 in HDAC8. This way, in complexes with HDAC6-CD2 and HDAC8, SAHA forms three hydrogen bonds with the catalytic tyrosine and histidine residues. In the complex with HDAC1, SAHA forms a hydrogen bond with His140 and Asp176 and Gly301 residues, leaving dipole–dipole interactions with Tyr303 and His141 residues.

Following molecular docking results, the obtained free energy values and binding mode were analyzed, prioritizing ligand orientations relative to the established pharmacophore for HDACis to favor binding modes directed towards the Zn^2+^ and catalytic residues. The results of the virtual screening for all compounds are detailed in Tables S4–S8 and selection criteria in Figures S2 and S3 and Tables S10 and S11 in the Supplementary Materials. For the discussion of docking results, only the binding modes of compounds **A6**, **A7**, **B6**, and **B7** are detailed. These compounds were selected as the best from a virtual screening process. They were chosen because they have the same CAP group in both families and were selected as the most promissory.

In the molecular docking analysis of the designed molecules on HDAC1, the free energy of binding, the binding mode, and the interactions formed were considered. The binding mode was further delineated by the percentage of interactions with key amino acid residues involved in the catalytic hydrolysis process, such as Tyr303, His140, and His141, as well as those residues close to Zn^2+^ such as Asp178, His180, and Asp264. Amino acids that generate hydrophobic interactions along the catalytic tunnel, such as Phe150 and Phe205, were also included. The types of intermolecular interactions formed were evaluated, and different scores were assigned based on the predicted relative strength of these interactions.

**Figure 5:**
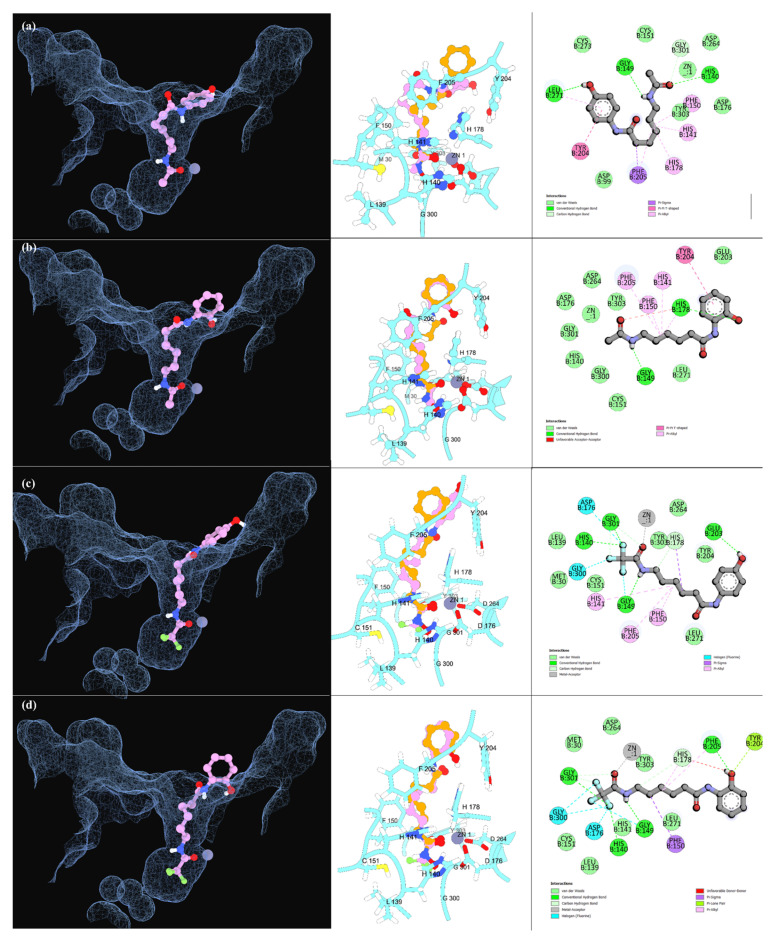
Binding mode against HDAC1 of *N*-acetylated derivatives (**a**) **A6** and (**b**) **A7**, and *N*-trifluoroacetylated derivatives (**c**) **B6** and (**d**) **B7**, represented as pink balls and sticks, with the co-crystallized ligand SAHA shown as orange balls and sticks.

The binding mode of *N*-acetylated compounds (**A1**–**A32**), was consistent with the reported pharmacophore of HDACi, showing interaction with all mentioned residues. Specifically, the *N*-acetyl group reaches the bottom of the cavity, forming a coordination interaction with the Zn^2+^. This interaction is favored by the formation of the donor hydrogen bond between the amide group and Gly149. The orientation of the six-carbon aliphatic chain is linearly positioned along the cavity, generating hydrophobic interactions with Phe150 and Phe205. In general, the aromatic CAP group exhibited π-π interactions with Tyr204. The hydroxylated derivatives **A6** and **A7** performed hydrogen bond interaction with Leu271 and His178, respectively (Figure 5a,b). The case of *N*-trifluoroacetylated compounds **B1**–**B32**, as ZBG make a higher number of intermolecular interactions at the bottom of the cavity. These interactions are carbon–hydrogen bonds and hydrogen bonds with halogens (also known as sigma holes) [38,39], as well as hydrogen bonds with residues such as Gly149, Gly301, and His140 (Figure 5c,d, for **B6** and **B7**). From a thermodynamic perspective, this contributes to enhanced stability of the inhibitor-HDAC1 complex. However, the energetic contribution manifested as affinity energy is only marginally higher than that observed for its analogs, **A6** and **A7**.

**Figure 6:**
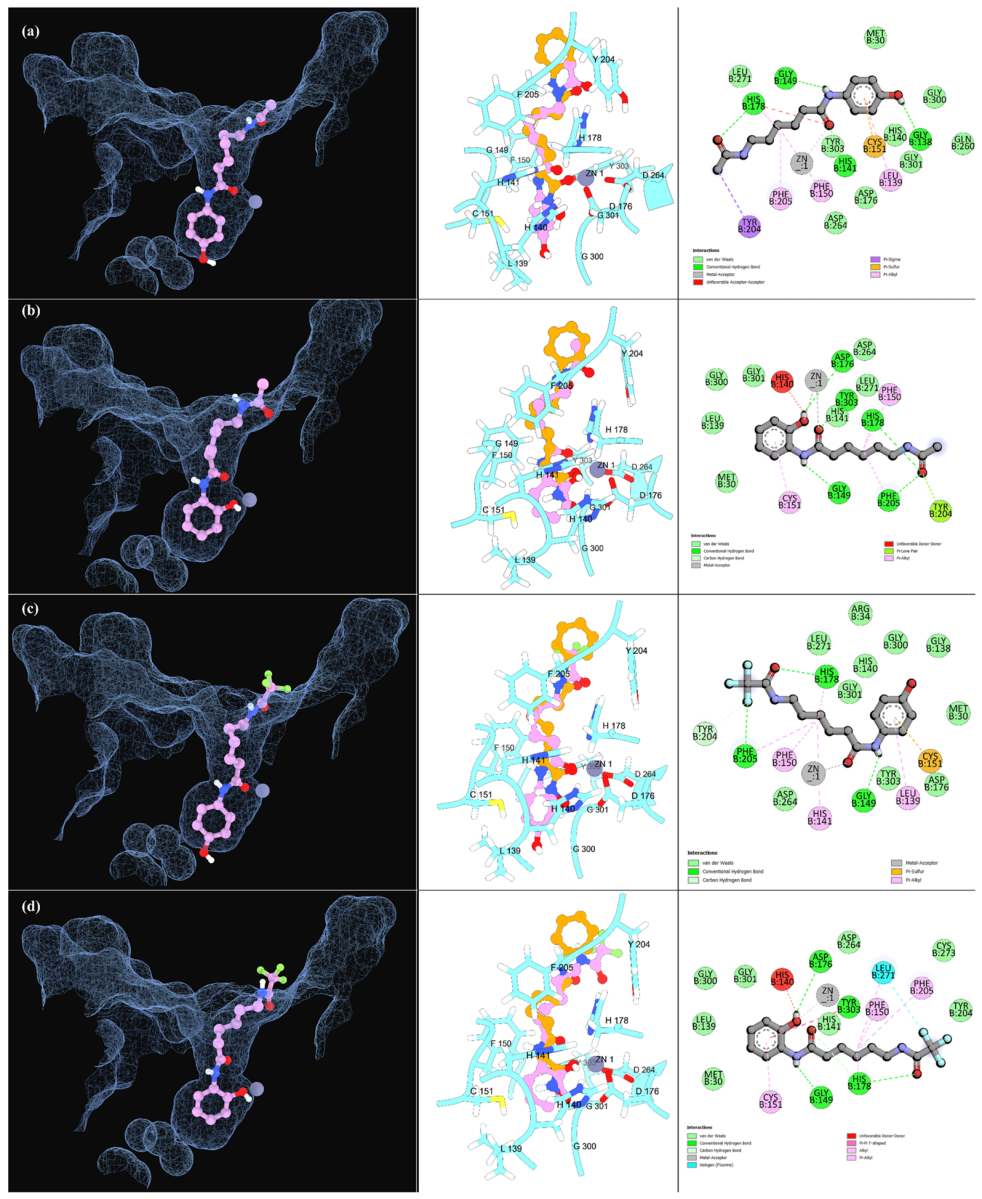
Second binding mode against HDAC1 of *N*-acetylated derivatives (**a**) **A6** and (**b**) **A7**, and *N*-trifluoroacetylated derivatives (**c**) **B6** and (**d**) **B7**, represented as pink balls and sticks, with the co-crystallized ligand SAHA shown as orange balls and sticks.

It is noteworthy that some of the compounds exhibited a second binding mode, in which the orientation was opposite to the previously discussed positions (Figure 6a–d). The presence of this second binding mode was only observed in compounds that possessed non-bulky CAP groups and substitutions capable of interacting with the Zn^2+^ [40]. This observation can be attributed to the topology of the HDAC1 active site, which features a wider catalytic channel. This extension of the catalytic cavity is present in HDAC8 but absent in HDAC6 [41]. Consequently, the existence of this second binding mode suggests that these molecules may possess heightened inhibitory potency. This finding aligns with the observations reported in studies that have demonstrated the capacity of certain molecules to engage with the active site of an enzyme through two distinct binding modes, thereby augmenting its inhibitory or modulatory activity. This strategy has proven to be effective in obtaining more efficient bioactive molecules.

This second binding mode was observed in derivatives **A6**, **A7**, **B6**, and **B7**. The *N*-(4-hydroxyphenyl)-substituted derivatives **A6** and **B6** were oriented with the CAP group at the bottom of the catalytic cavity, generating the interaction of monodentate coordination of Zn^2+^ with the carbonyl group of the benzamide (Figure 6a,c, respectively). In contrast, **A7** and **B7** (*N*-(2-hydroxyphenyl)-substituted) exhibited a binding mode where the CAP group adopted a bidentate configuration on the Zn^2+^, with the hydroxyl group at position 2 and the carbonyl group of the amide as the responsible entities (Figure 6b,d). This binding mode is analogous to that exhibited by *N*-(2-aminophenyl) benzamide derivatives (such as Chidamide, Mocetinostat and Entinostat) [42,43], and to another recently reported inhibitor characterized by a salicylamide substituent as ZBG [44]. These examples were shown to be selective towards class 1 HDACs. In addition, in this work, the *N*-acetyl derivatives **A1**–**A10**, **A12**, **A16**, and **A17**, as well as the *N*-trifluoroacetyl derivatives **B1**–**B10**, **B12**, **B16**, and **B17**, showed the same behavior as had been previously described. The binding energies of the ligands that exhibit this duality in their molecular recognition were, surprisingly, generally more exergonic in the complexes formed with the CAP groups.

**Figure 7:**
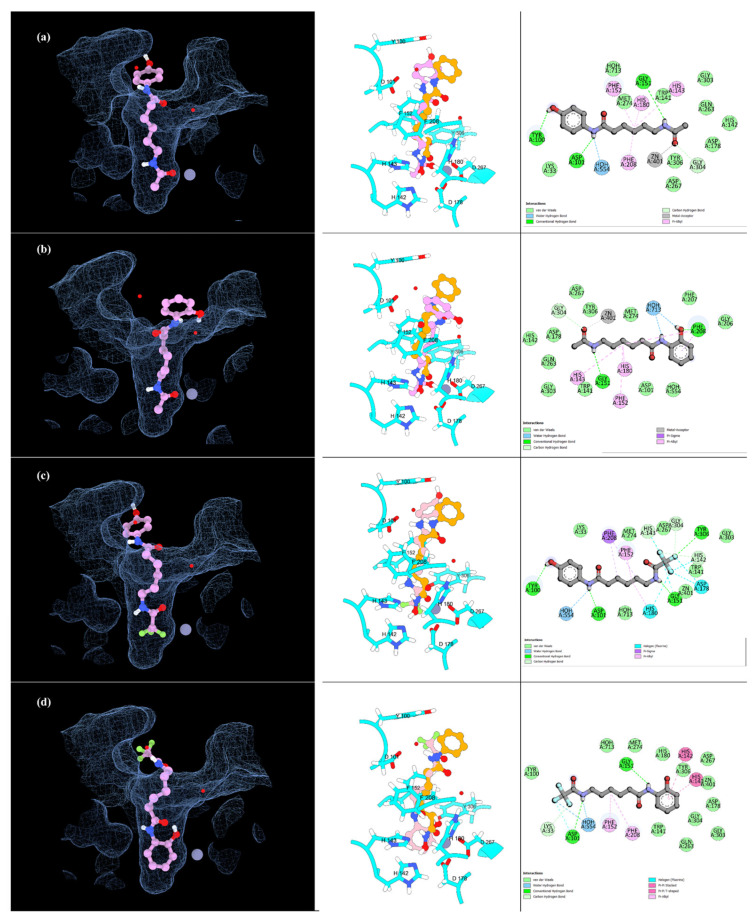
Binding mode against HDAC6-CD2 of *N*-acetylated derivatives (**a**) **A6** and (**b**) **A7**, and *N*-trifluoroacetylated derivatives (**c**) **B6** and (**d**) **B7**, represented as pink balls and sticks, with the co-crystallized ligand SAHA shown as orange balls and sticks.

In the context of the above structural analysis, molecular docking results of the target compounds on HDAC6-CD2 and HDAC8 isoforms showed similar binding modes. On HDAC6, according to our calculations, we did not identify any secondary binding modes, which is attributed to the shorter topology of the catalytic domain compared to HDAC1 and HDAC8, preventing the fitting of aromatic rings even without bulky substituents [45]. In the representative examples, the acetylated derivatives **A6** and **A7** formed a monodentate interaction with Zn^2+^ through the carbonyl group of acetamide, maintaining the hydrophobic interactions along the catalytic tunnel with the LINKER and CAP groups (Figure 7a,b). Remarkably, analogs **B6** and **B7** not only retained the binding mode but also increased the polar interactions at the bottom of the binding pocket between the fluorine atoms and Gly582, Cys584, Asp612, His614, and Try745 (Figure 7c,d). According to studies of structure–activity relationships, HDAC inhibitors that have high potency and selectivity over HDAC6 persist with hydrogen bond interactions with Gly582, His 614, and Try745 [45,46,47].

The overexpression of HDAC8 has been closely related to cancer progression, including cancer cell proliferation, metastasis, immune evasion, and drug resistance [48]. Consequently, it has been proposed as an excellent drug target to explore the affinity of our designed molecules.

**Figure 8:**
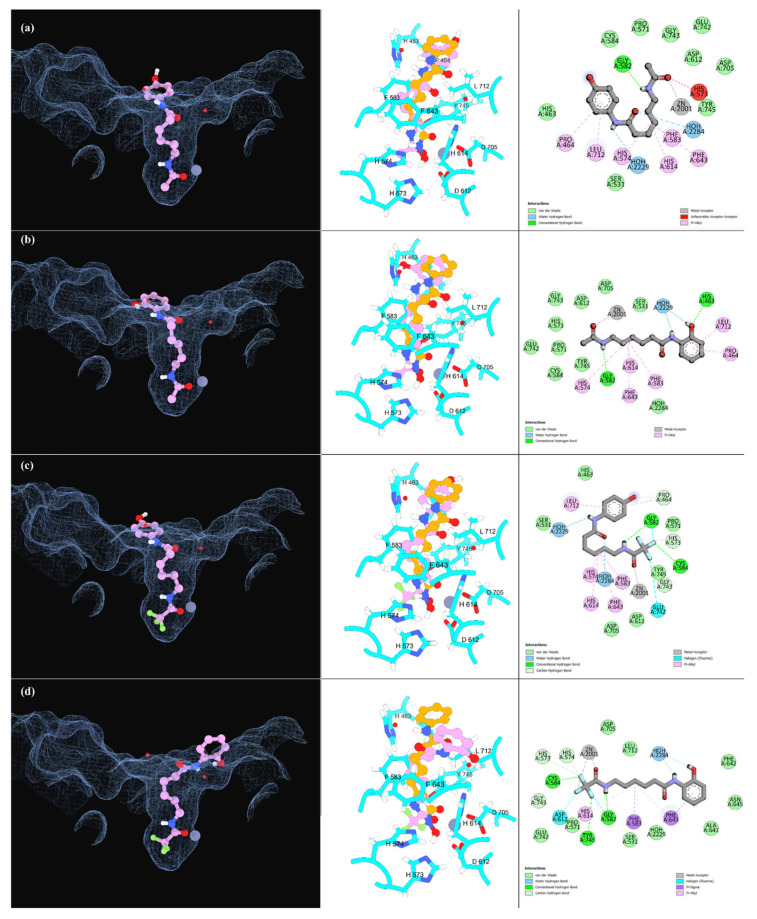
Binding mode against HDAC8 of *N*-acetylated derivatives (**a**) **A6** and (**b**) **A7**, and *N*-trifluoroacetylated derivatives (**c**) **B6** and (**d**) **B7**, represented as pink balls and sticks, with the co-crystallized ligand SAHA shown as orange balls and sticks.

In general, *N*-acetylated and *N*-trifluoroacetylated molecules adopted a binding mode consistent with the molecular recognition expected for the HDACi pharmacophore. The -NHCOCH_3_ derivatives were oriented towards the Zn^2+^ in a monodentate manner, generating exergonic clusters with a wide conformational diversity due to several protein surfaces close to active site. The calculated binding affinity values for the ligands **A6** and **A7** were −7.18 and −7.11 kcal/mol, respectively. Crucial and interesting non-bon interactions were observed in the conformational anchoring by hydrogen bonds between Tyr100, a water molecule (located at the surface of the catalytic tunnel) and the 4-OH and 2-OH groups of the CAP groups in **A6** and **A7**, respectively (Figure 8a,b). In contrast, derivatives **B6** and **B7** (-NHCOCF_3_) exhibited reduced ΔG affinity values compared to their structural analogs, with values of −6.25 and −5.90 kcal/mol, respectively. Notably, the anticipated interactions were preserved in both cases. The topology of the HDAC8 binding pocket was found to favor the emergence of a second binding mode for **A6**, **B6**, and **B7**. This binding mode exhibited a lower binding energy compared to the major binding modes. The data obtained from this study suggest a theoretical higher affinity preference of NHCOCF_3_ derivatives over HDAC1 isoform through the observed secondary binding mode (Table S3 in Supplementary Materials).

Finally, the selection of the best-ranked molecules was performed by a consensus summation of several parameters: (1) ADMET property values, (2) binding percentage, (3) interaction score, and (4) affinity free energy (kcal/mol) values. This resulted in the identification of the best molecules (considering both family **A** and **B** ligands) for each HDAC isoform, taking SAHA as a reference (∑ABCD ≥ SAHA score). The overall results are presented in detail in Tables S4–S9 in the Supplementary Materials. Analysis of the overall scores using Venn diagrams revealed that six ligands from family **A** showed in silico selectivity for HDAC1 (**A1**, **A14**, **A16**, **A20**, **A21**, **A24**, and **A28**), with one for HDAC6 (**A16**) and none for HDAC8 (Figure S2 and Table S10), whereas four ligands from family **B** showed a preference for HDAC1 (**B3**, **B9**, **B21**, and **B28**), one for HDAC6 (**B13**), and only **B11** for HDAC8 (Figure S3 and Table S11). Consequently, in the final selection of ligands with priority for chemical synthesis, we considered those that, according to the global analysis, were related to the three isoforms, as described in Table 2.

### 2.3. Chemistry

**Scheme 1:**
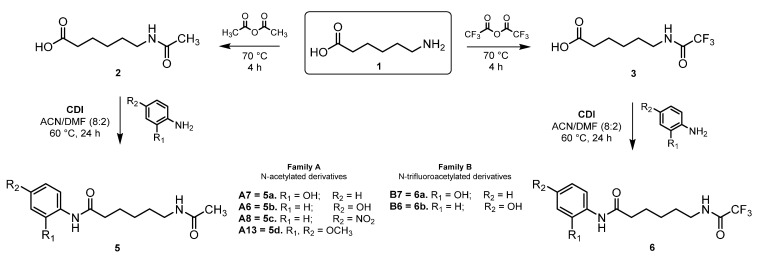
Chemical synthesis route leading to the production of compounds of family A (*N*-acetylated; **5a**–**d**) and family B (*N*-trifluoroacetylated; **6a**–**b**). The numbers for the final products (**5** and **6**) were assigned according to the order of the reagents used in the chemical synthesis.

Ligands with higher scores than SAHA in ADMET, molecular docking, and preference for all three isoforms evaluations were selected for chemical synthesis. The synthetic route for the target compounds characterized by the presence of the aliphatic chain and the *N*-acetyl group (mimetic of the natural substrate acetyl-lysine) is illustrated in Scheme 1. Acetamide **2** and trifluoroacetamide **3** were synthesized from 6-aminohexanoic acid (**1**) through an *N*-acetylation reaction with acetic anhydride and trifluoroacetic anhydride, respectively; the mixture was left at 70 °C for 48 h, obtaining good yields (70–80%). The *N*-acetylated final products **5a**–**d** and the *N*-trifluoroacetylated products **6a**–**b** were obtained from the treatment of **2** and **3** with the coupling agent CDI at 60 °C for 3 h, using a mixture of ACN/DMF (8:2) as solvent, which formed the corresponding activated acid in situ. The different anilines **4a**–**d** were then added and allowed to react for 24 to 72 h, depending on the nucleophilic character of each aniline used, to give the corresponding end products in moderate yields. The chemical structures of the final compounds were characterized by ^1^H NMR, ^13^C NMR, mass spectrometry, and IR spectroscopy (see Section 3.2 of Materials and Methods).

### 2.4. Biological Evaluation

#### 2.4.1. Assessment of HDAC Inhibitory Activity

**Figure 9:**
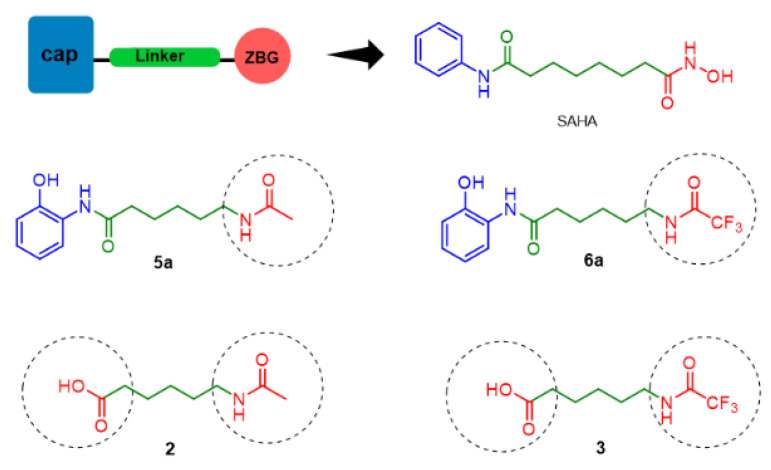
Structural features of compounds **2**, **3**, **5a**, and **6a**. ZBG portion colored red, LINKER portion colored in green, and CAP portion colored in blue.

To demonstrate the capacity of the synthesized compounds to interact with the active site of HDACs, thereby preventing the deacetylation of acetyl-lysine, we evaluated the HDAC inhibitory activity of compounds **2**, **3**, **5a**, and **6a** by means of a fluorescence in vitro assay. In this initial approach, compounds **5a** and **6a**, which possess different ZBGs and shared CAP groups, were evaluated, including their respective precursors **2** and **3**, which lack a CAP group but have two ZBG groups. Compound **2** contains an *N*-acetamide group, while compound **3** has an *N*-trifluoroacetamide moiety that acts as a ZBG, but both contain a carboxylic acid moiety (Figure 9), which has been widely reported in the literature as a ZBG in several HDAC inhibitors [49,50]. Specifically, we evaluated the effect of the absence and presence of CAP groups on HDAC inhibition, as well as the inhibitory effect of a third possible ZBG.

**Figure 10:**
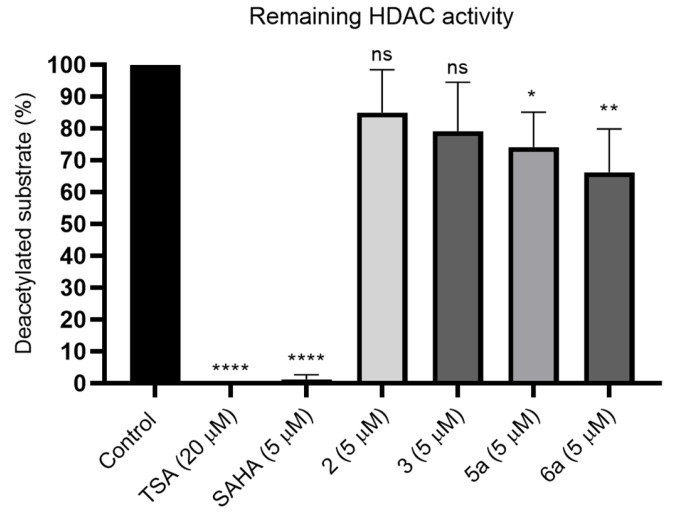
HDAC-inhibitory activity of compounds **2**, **3**, **5a**, and **6a** in nuclear extracts of HeLa cells. The enzymatic activity of the compounds is expressed as a percentage relative to the positive controls TSA (inhibition control of kit) and SAHA (drug of reference). The experiments were performed in triplicate with n = 3 for each condition. Data are presented as means ± SE. Statistical significance was determined by one-way ANOVA (F = 48.06, *p* < 0.0001) followed by Dunnett’s post hoc test. ns = no statistical significance; Asterisks indicate statistically significant differences compared to the control: * *p* < 0.05, ** *p* < 0.01, **** *p* < 0.0001.

In this in vitro assay, residual HDAC activity was measured in a nuclear extract of HeLa cells after incubation with the different compounds at 5 µM, followed by single endpoint fluorescence measurement. Fluorometry showed that the tested compounds exhibited low inhibitory potency compared to TSA and SAHA, which reduced HDAC activity almost completely. The observed discrepancy in potency can be attributed to the high affinity of the hydroxamic portion for the Zinc atom, which is consistent with its high electronic polarizability. This characteristic enables molecular cross-reactivity with other metal cofactor-dependent therapeutic targets [30]. Although compounds **2**, **3**, **5a**, and **6a** showed a slight decrease in HDACs activity, statistical significance could only be observed for compounds **5a** and **6a**, highlighting the influence of the CAP group on the inhibitory activity. These results indicate that the absence of the CAP group in compounds **2** and **3** decreases their HDAC inhibitory activity, even when presenting a carboxylic acid as second ZBG, which did not contribute significantly to the activity; previous reports have communicated their weak binding capacity to Zn^2+^, especially short-chain fatty acids, showing IC_50_ ≅ mM [20,51]. Furthermore, among the ZBGs evaluated, compounds with an *N*-trifluoroacetyl group showed a relatively higher inhibitory activity compared to those with an *N*-acetyl group (**3** > **2** and **6a** > **5a**). Compound **6a** showed the best inhibition profile, which correlates with computational predictions showing that molecules with CAP groups, such as 2-hydroxyphenylacetamide, can interact with the catalytic site in a dual manner, presenting two binding modes and therefore higher potency (Figure 10).

As was previously demonstrated, the structural modifications to the pharmacophore resulted in alterations to the HDAC inhibitory activity. Furthermore, it has been documented that the substitution of the chelating agent from SAHA with a trifluoromethylketone group (-COCF_3_) influenced the reaction mechanism, suggesting a secondary conformational change that stabilizes the ligand–enzyme complex. This effect is reflected in the slow inhibition kinetics of trifluoromethylketone derivatives and, from a technical perspective, in the pre-incubation time of the inhibitor, as a prolonged pre-incubation period corresponds to reduced IC_50_ values [52,53]. In the above context, a comparable scenario is likely to be encountered with our *N*-trifluoroacetamide and *N*-acetamide derivatives. Consequently, it will be worthwhile to characterize the enzymatic inhibition profile of **5a** and **6a**, in pursuit of a slow-binding inhibitory reversible character, as observed for trifluoromethylketone-derivative inhibitors [54].

#### 2.4.2. Cell Survival Assessment of Breast Cells Lines

The antiproliferative activity of compounds **2**, **3**, **5a**–**d**, and **6a**–**b** and SAHA (positive control) against BC cell line MDA-MB-231 was assessed using the 3-(4,5-dimethylthiazol-2-yl)-2,5-diphenyltetrazolium bromide (MTT) assay. The assay was performed after 72 h of incubation, as suggested in the literature for HDAC inhibition molecules. The mechanisms of action of HDAC inhibitors, such as apoptosis induction, cell cycle arrest, and changes in gene expression, often require a longer incubation period to fully manifest [55,56]. This effect is undoubtedly related to the mechanism of enzyme inhibition. SAHA has been found to be a reversible inhibitor of rapid binding and dissociation, independent of concentration, making it a potent *pan*-inhibitor.

Compounds **5b**, **6a**, and **6b** exhibited significant cytotoxic activity after 72 h of treatment (Figures S33 and S34, Supplementary Materials). The compounds with the highest cytotoxicity in MDA-MB-231 cells were selected for further evaluation in other cell lines.

Subsequently, the effect on cell viability of compounds **5b**, **6a**, **6b**, and SAHA was evaluated in the breast cancer cell line MCF-7 at 72 h of incubation. The results confirmed the cytotoxic activity of these compounds also in this cell line.

**Figure 11:**
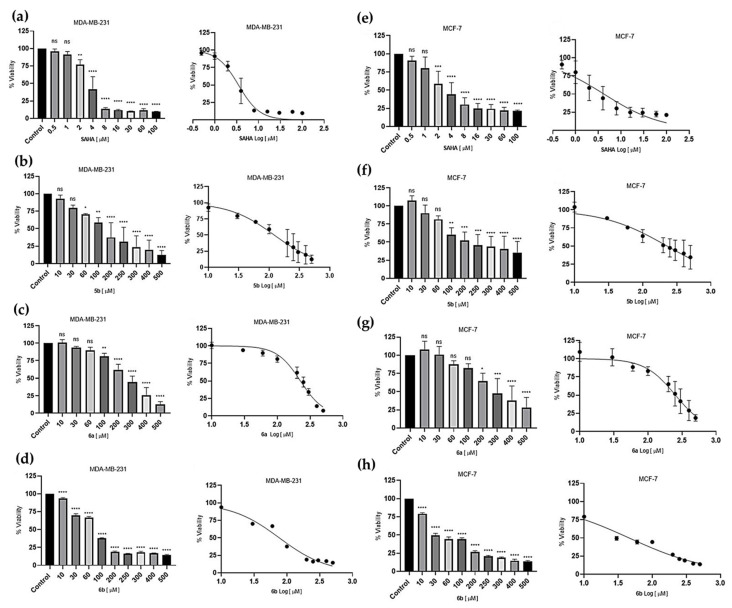
Viability of MDA–MB–231 cells treated with (**a**) SAHA, (**b**) **5b**, (**c**) **6a**, and (**d**) **6b** and MCF-7 cells treated with (**e**) SAHA, (**f**) **5b**, (**g**) **6a**, and (**h**) **6b** evaluated by MTT assay. Calculation of IC_50_ was obtained by fitting the cytotoxicity responses to a variable slope sigmoidal equation (log_inhibitor_ vs. normalized response). The experiments were performed in triplicate with n = 6 for each concentration. Data are presented as means ± SE. * *p* < 0.05 obtained from one-way ANOVA analysis and Dunnett’s post hoc test. ns = no statistical significance; (****) asterisks are used to denote statistical significance in relation to the control group.

Treatment of MDA-MB-231 cells with compounds **5b**, **6a**, and **6b** exhibited IC_50_ = 121.3 µM, 225.5 µM, and 76.7 µM at 72 h, respectively (Figure 11a–d). In MCF-7 cells, the IC_50_ = 229.9 µM for **5b**, 253.8 µM for **6a**, and 45.7 µM for **6b** (Figure 11e–h) (see Figures S35–S42 of Supplementary Materials). In contrast, SAHA exhibited a markedly cytotoxic effect in both cell lines, with IC_50_ = 3.5 µM for MDA-MB-231 and IC_50_ = 4.2 µM for MCF-7 at 72 h. These findings are consistent with those reported by other research groups [57,58]. While it is evident that the *N*-acetylated derivative (**5b**) and the *N*-trifluoroacetylated derivatives (**6a** and **6b**) exhibited reduced growth inhibitory activity compared to the reference drug, this observation can be rationalized by the bioisosteric shift in ZBG that modifies the catalytic mechanism, selectivity, and potency. This phenomenon is exemplified by some ZBGs, including benzamide [59], hydrazide [60], ethanolamine [61], 1-amino-2-propanol, and trifluoropyruvamides [62].

The results of this study indicated that among the trifluoroacetamide derivatives, **6b** (4-hydroxyphenyl CAP-substituted) exhibited the most significant cytotoxic effect in both cell lines. This finding indicates a significant contribution to the stereoelectronic environment of the CAP group, since **6a** presents a 2-hydroxyphenyl substitution and yet was less active than the *N*-acetylated derivative **5b** (4-hydroxyphenyl substituted). The stereoelectronic effect and the position of the hydroxyl group in the enhancement of cytotoxic activity were also observed in the design and synthesis of *N*-(2-hydroxyphenyl)-2-propylpentanamide derivatives [63]. Consequently, the gathered evidence lends further credence to the idea that the ZBG can determine the binding profile of HDACi (particularly hydroxamic), though non-hydroxamic inhibitors also depend on the hydrophobic character of LINKER and, naturally, on CAP [64].

**Figure 12:**
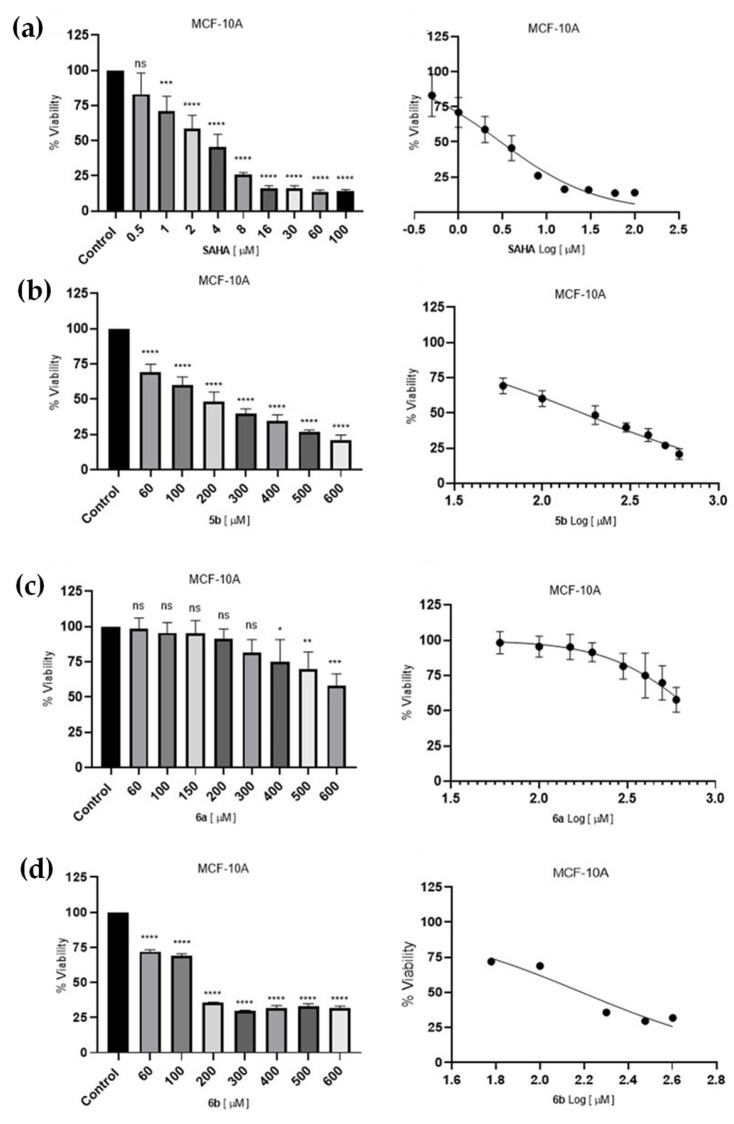
Viability of MCF–10A cells treated with (**a**) SAHA, (**b**) **5b**, (**c**) **6a**, and (**d**) **6b** evaluated by MTT assay. Calculation of IC_50_ was obtained by fitting the cytotoxicity responses to a variable slope sigmoidal equation (log_inhibitor_ vs. normalized response). The experiments were performed in triplicate with n = 6 for each concentration. Data are presented as means ± SE. * *p* < 0.05 obtained from one-way ANOVA analysis and Dunnett’s post hoc test. ns = no statistical significance; (****) asterisks are used to denote statistical significance in relation to the control group.

SAHA and other hydroxamic HDACi have shown efficacy in leukemia therapy. Since the use of these compounds is promising for solid tumors and non-malignant diseases, structural modifications that improve the pharmacokinetic profile, widen the therapeutic window, and reduce the toxic risk potential are desirable. Therefore, a non-cancerous mammary epithelial cell viability assay, MCF-10A, was used to determine the cytotoxic selectivity of the most active compounds **5b**, **6a**, and **6b** (see Figures S43–S46 of Supplementary Materials). In this regard, SAHA demonstrated high levels of toxicity in MCF-10A cells, with an IC_50_ = 3.0 µM, indicating minimal safety margins between cancerous and healthy cells. In contrast, compounds **5b** and **6a** exhibited IC_50_ = 168.6 µM and 120 µM, respectively, while **6a** reached concentrations exceeding 600 µM (Figure 12).

With these data, the selectivity index could be determined. If the value was >1, it is considered that the compound has greater activity against cancer cells and less toxicity to normal cells [65]. SAHA did not exceed the selectivity parameter because it generates high genetic instability in the cells. In a previous study, a group of hydroxamic HDACi were shown to be clastogenic and to induce DNA strand breaks in non-malignant V79 cells, as demonstrated by micronucleus and comet assays, histone H2AX (γH2AX) foci formation analysis, DNA damage response assays, and by using VC8 hamster cells defective in DNA double-strand break repair [66]. Conversely, the *N*-acetylated derivative **5b** was at the upper limit of the ratio. Despite the high IC_50_ values presented at **6a**, it exhibited a selectivity index that was twice as high. Finally, **6b** was twice as selective for healthy cells as compared to triple-negative breast cancer cells and was three times safer when treating luminal MCF-7 breast cancer cells (Table 3).

The results demonstrate that the new compounds, particularly the *N*-trifluoroacetyl derivatives **6a** and **6b**, exhibit a superior profile in terms of their cytotoxic activity. They are effective in killing cancer cells at concentrations that are significantly lower than those required to cause harm to healthy cells, thus rendering them a safer alternative.

## 3. Materials and Methods

### 3.1. Computational Design

A ligand-based design approach was employed, applying bioisosteric exchanges on the classical pharmacophore group of HDAC inhibitors, with the objective of achieving structural optimization through computational predictions, including physicochemical, pharmacokinetic, and toxicological properties, as well as a structure–affinity relationship analysis of the designed ligands against HDACs isoforms relevant in breast cancer.

#### 3.1.1. Ligands Construction

The two-dimensional structure of the ligands was constructed using ChemDraw 18.0 software (Revvity Signals Software Inc., Waltham, MA, USA) and subsequently converted to their three-dimensional projection with Chem3D 18.0. The three-dimensional structures were minimized using a molecular mechanic’s force field (MM2), and a geometric pre-optimization was subsequently performed employing the Merck Molecular Force Field (MMFF94), which integrated a dielectric constant of ε = 79.99. Finally, the AM1 semi-empirical method for conformational optimization was applied in Gaussian 09 software.

#### 3.1.2. ADMET Properties Prediction

A computational ADMET property prediction was conducted using five web servers that are commonly utilized in the field of drug design: 1. Molinspiration (https://www.molinspiration.com/, accessed on 20 June 2023. Slovensky Grob, Slovak Republic); 2. SwissADME (http://www.swissadme.ch, accessed on 19 June 2023. Swiss Institute of Bioinformatics of University of Lausanne, Lausanne, Switzerland); 3. ProTox II (https://tox-new.charite.de/protox_II/, accessed on 21 June 2023. Structural Bioinformatics Group of Charite University of Medicine, Berlin, Germany); 4. Osiris Property Explorer (https://www.organic-chemistry.org/prog/peo/, accessed on 20 June 2023. Organic Chemistry Portal, Buckten, Switzerland); and 5. SmartCyp (https://smartcyp.sund.ku.dk/mol_to_som, accessed on 25 June 2023. University of Copenhagen, København, Denmark).

Molinspiration and SwissADME web servers were employed to calculate physicochemical properties, including molecular weight, number of rotatable bonds, partition coefficient (Log P), number of hydrogen bond acceptors and donors, topological polar surface area (TPSA), and molar refractivity. Furthermore, drug-likeness properties were evaluated in accordance with the established criteria, including Lipinski’s rule of five, Ghose’s filter, Veber’s rule, Egan’s rule, and Muegge’s criteria. The potential presence of undesirable fragments, such as Pan-Assay Interference Compounds (PAINS) and Brenk’s undesirable fragments was also considered; these algorithms were run in SwissADME [67].

The toxicity profiles of the proposed ligands were predicted using the ProTox II and Osiris servers, with consideration of adverse effects including mutagenicity, tumorigenicity, irritability, reproductive effects, hepatotoxicity, immunotoxicity, and binding to potential molecular targets that may result in adverse effects. Additionally, predictions regarding metabolism and pharmacokinetics were conducted using the SMARTCyp and SwissADME platforms. The probability of biotransformation reactions, inhibition of cytochromes (CYP1A2, CYP2C19, CYP2C9, CYP2D6 and CYP3A4), ability to cross the blood–brain barrier (BBB), gastrointestinal permeability, and possible binding to P-glycoprotein (a mechanism of anticancer drug resistance through efflux) were included in the analysis [68].

#### 3.1.3. Molecular Docking Simulations

The structural information for the HDAC isoforms utilized in this study was obtained from the Protein Data Bank (PDB). For HDAC1, the PDB ID: 4BKX (3.00 Å resolution) was employed; for HDAC6-SAHA complex, the PDB ID: 5EEI was selected, (resolution of 1.32 Å). For HDACSAHA complex, the PDB ID: 4QA0 (resolution of 2.42 Å) was chosen. In all cases, ligands and surrounding water molecules on the proteins were removed, retaining only those that were structurally conserved at the catalytic site and important in the formation of the HDAC-inhibitor complex [69]. Likewise, all ions and non-functional organic molecules were removed from the crystalline complexes. Finally, the protein structures were energetically minimized using Swiss-PDB-Viewer (https://spdbv.unil.ch/energy_tut.html, accessed on 26 June 2023) and Chimera 1.17 software (https://www.rbvi.ucsf.edu/chimera/, accessed on 26 June 2023) with the AMBER ff14SB force field [70]. Any remaining structural errors were corrected using SAVES 6.0 (https://saves.mbi.ucla.edu/, accessed on 30 June 2023) and MolProbity (http://molprobity.biochem.duke.edu/, accessed on 30 June 2023) to address steric clashes.

The parameterization of proteins and ligands was conducted in MGLTools 1.5.6 (https://ccsb.scripps.edu/mgltools/1-5-6/, accessed on 26 June 2023), with the addition of Kollman charges and Gasteiger charges, respectively. The dimensions of the molecular docking grid box were maintained at 42 Å^3^ for HDAC1 (PDB ID: 4BKX), 56 Å^3^ for HDAC6 (PDB ID: 5EDU), and 42 × 56 × 42 Å for HDAC8 (PDB ID: 4QA0), with a spacing of 0.375 Å^3^ and centering in the catalytic cavity. In all cases, the modified Autodock4 (Zn) force field was employed [71]. The search algorithm employed was a Lamarckian genetic that analyzed 100 conformations with 25,000,000 energetic evaluations. The energetic results and molecular interactions generated by the reference drugs and the designed molecules were analyzed using MGLTools 1.5.6, Chimera, PyMOL 3.0 (https://www.pymol.org/, accessed on 26 June 2023) and BIOVIA Discovery Studio (https://discover.3ds.com/discovery-studio-visualizer-download, accessed on 26 June 2023, academic license).

#### 3.1.4. Hit Identification

To identify the most promising compounds as candidates, those with an ADMET score higher than SAHA were prioritized. This was followed by further prioritization based on a molecular docking free energy value close or higher than SAHA. Finally, the binding mode was analyzed, quantifying the percentage of coincidence interactions between the reference drug and the designed ligands, over the catalytic cavity of each HDAC isoform. Tables S4–S11 (Supplementary Materials) show in detail the score assigned to each calculated property, taking the SAHA inhibitor as reference.

### 3.2. Chemical Synthesis

The chemical synthesis utilized reagents and solvents procured from Sigma Aldrich^®^ (St. Louis, MO, USA). The reaction course was monitored by thin-layer chromatography (Kiessegel 60 F_254_, Sigma Aldrich, St. Louis, MO, USA), using ultraviolet radiation at 254 nm and iodine vapors as developing agents, for the detection of reaction intermediates and products. The yield percentage of each reaction was calculated after purification by column chromatography. Melting points were determined using a Stuart SMP11 (Bibby Scientific, Stone, UK) melting point apparatus. The chemical purity of the final products was evaluated by high-performance liquid chromatography (HPLC) using an Agilent 1200 Infinity Series (Agilent Technologies Inc., Waldbronn, Germany). Subsequently, electrospray ionization mass spectrometry (ESIMS) was employed for the detection of the reaction products, using a 6545XT AdvanceBio LC/Q-TOF (Agilent Technologies Inc., Singapore) quadrupole time-of-flight system (figures of MS spectra are shown in Figures S25–S32 in Supplementary Materials). Finally, the structural connectivity of the synthesized molecules was determined by nuclear magnetic resonance (NMR) spectroscopy of ^1^H and ^13^C, using a Varian Mercury 300 MHz spectrometer (Varian, Crawley, UK), with DMSO-*d*_6_ as the solvent. Chemical shift (δ) values are expressed in parts per million (ppm) and coupling constants ^n^*J* (H–H) are expressed in Hz. Signal multiplicities are identified as: *s* (singlet), *d* (doublet), *t* (triplet), *q* (quartet), or *m* (multiplet), NMR spectra are shown in Figures S11–S24 in the Supplementary Materials.

#### 3.2.1. General Procedure for the Synthesis of *N*-Acetylated and *N*-Trifluoroacetylated Analogs

A solution containing 1.0 g (7.62 mmol) of 6-aminohexanoic acid (**1**) and 3.6 mL of acetic anhydride (38.0 mmol) or 3.17 mL trifluoroacetic anhydride (22.8 mmol), was prepared in a flask equipped with a magnetic stirrer. The solution was heated at 70–72 °C for 48 h, after which the reaction was quenched by the addition of 3.0 mL of water, which was kept in stirring for about 15 min. Subsequently, the extraction process was initiated with the addition of 3 × 25 mL AcOEt. The organic phase was washed with 4 × 25 mL distilled water reaching neutral pH, dried over anhydrous Na_2_SO_4_, filtered, and evaporated to dryness. The product, 6-acetamidohexanoic acid (**2**) or 6-(2,2,2-trifluoroacetamido) hexanoic acid (**3**), was purified by recrystallization using a solvent mixture Dichloromethane and Methanol.

#### 3.2.2. General Procedure for Amide Coupling Reaction Using CDI

In a flask provided with magnetic stirring and condensation system, 0.2 g of 6-acetamidohexanoic acid (**2**, 1.17 mmol) or 6-trifluoroacetamidohexanoic acid (**3**, 0.88 mmol) were added and dissolved with 4.0 mL of a mixture of ACN/DMF (10:1) at 60 °C. To this solution, 0.22 g (1.38 mmol) of carbonyldiimidazole (CDI) dissolved in 2.0 mL of can was added and allowed to react for 3 h under nitrogen atmosphere. After this time, 1.7 mmol (1.7 eq) of the different aromatic amines (**4**) were added and allowed to react for an additional 48 to 120 h, depending on reactivity of the amine, which was verified by thin-layer chromatography. The reaction was quenched by adding 25 mL of water and the reaction mixture was extracted with ethyl acetate (3 × 25 mL) and, subsequently, the organic phase was dried over anhydrous Na_2_SO_4_ and concentrated under reduced pressure using a rotary evaporator. The crude product of the reaction was purified by column chromatography using a mixture of Hexane:AcOEt in polarity gradient as mobile phase and silica gel as the stationary phase. Finally, the reaction yield was calculated based on the weight of the purified compound.

##### 6-Acetamidohexanoic Acid (2)

White solid, yield 60%, mp: 95–97 °C, R*_f_* = 0.11 (Hexane:AcOEt = 1:1).

**IR**: υ_max_ (cm^−1^) 3347 (N-H), 2945 (C-H), 2250–3450 (O-H acid), 1702 (C=O), 1556 and 1601 (C-N), and 1197 (C-O). **^1^H NMR** (300 MHz, DMSO-*d*_6_) δ 1.30–1.35 (*m*, 2H, CH_2_), 1.45–1.52 (*m*, 2H, CH_2_), 1.55–1.67 (*m*, 2H, CH_2_), 1.78 (*s*, 3H, CO-CH_3_), 2.20–2.22 (*t*, 2H, *J* = 7.1 Hz, CH_2_), 3.00–3.02 (*t*, 2H, *J* = 7.1 Hz, CH_2_), 7.72 (*s*, 1H, NH), and 11.87 (*s*, 1H, OH). **^13^C NMR** (75.5 MHz, DMSO-*d*_6_) δ 23.3 (CO-CH_3_), 24.4 (C-3), 26.1 (C-4), 29.7 (C-5), 34.0 (C-2), 38.9 (C-6), 170.7 (NH-CO-CH_3_), and 178.4 (C-1). **HRMS**, [M + Na]^+^: experimental = 196.0900, calculated = 196.0950.

##### 6-Acetamido-*N*-(2-hydroxyphenyl) Hexanamide (**5a**)

Orange solid, yield 70%, mp: 93–95 °C, R*_f_* = 0.22 (AcOEt).

**IR**: υ_max_ (cm^−1^) 3296 (N-H), 3250–2400 (O-H), 2936 (C-H), 1671 (C-O), 1623 (Ar-C-C), 1671 (C-O), and 1564–1595 (C-N). **^1^H NMR** (300 MHz, DMSO-*d*_6_) δ 1.04–1.17 (*m*, 2H, CH_2_), 1.22 (*m*, 2H, CH_2_), 1.34–1.46 (*m*, 2H, CH_2_), 1.60 (*s*, 3H, CO-CH_3_), 2.19 (*t*, 2H, *J* = 7.4 Hz, CH_2_), 2.83 (*dd*, 2H, *J* = 12.7, 6.6 Hz, CH_2_), 6.57 (*t*, 1H, *J* = 7.6 Hz, CH_2_), 6.66 (*d*, 1H, *J* = 6.8 Hz, Ar-H), 6.75 (*t*, 1H, *J* = 7.5 Hz, Ar-H), 7.49 (*d*, 1H, *J* = 7.8 Hz, Ar-H), 7.62 (*s*, 1H, NH-CO-CH_3_), 9.06 (*s*, 1H, OH), and 9.56 (*s*, 1H, Ar-NH). **^13^C NMR** (75.5 MHz, DMSO-*d_6_*) δ 22.91 (C8), 25.34 (C3), 26.41 (C4), 29.27 (C5), 36.25 (C2), 38.75 (C6), 116.26 (Cs 18 y 19), 119.27 (C13),122.63 (C11), 124.91 (C9), 126.70 (C10), 148.16 (C14), 169.21 (C7), and 172.18 (C1). **HRMS**, [M + Na]^+^: experimental = 287.1366, calculated = 287.1372.

##### 6-Acetamido-*N*-(4-hydroxyphenyl) Hexanamide (**5b**)

Brown solid, yield 89%, mp: 135–136 °C, R*_f_* = 0.12 (AcOEt).

**IR**: υ_max_ (cm^−1^) 3329 (N-H), 3102 (C-H), 3100–2300 (O-H), 2946 (C-H), and 1729 (C=O). **^1^H NMR** (300 MHz, DMSO-*d*_6_) δ 0.80–0.92 (*m*, 2H, CH_2_), 0.93–1.04 (*m*, 2H, CH_2_), 1.07–1.20 (*m*, 2H, CH_2_), 1.36 (*s*, 3H, CH_3_), 1.81 (*t*, 2H, *J* = 7.4 Hz, CH_2_), 2.60 (*dd*, 2H, *J* = 12.7, 6.6 Hz, CH_2_), 6.25 (*d*, 2H, *J* = 8.8 Hz, Ar-H), 6.94 (*d*, 2H, *J* = 8.8 Hz, Ar-H), 7.39 (s, 1H, NH-CO-CH_3_), 8.72 (*s*, 1H, OH), and 9.17 (*s*, 1H, Ar-NH). **^13^C NMR** (75.5 MHz, DMSO-*d*_6_) δ 22.50 (C8), 25.09 (C3), 26.27 (C4), 28.46 (C5), 36.28 (C2), 38.52 (C6), 115.05 (Cs 11 and 13), 120.94 (C10 and 14), 131.12 (C9), 153.17 (C12), 168.99 (C7), and 170.51 (C1). **HRMS**, [M + Na]^+^: experimental = 287.1366, calculated = 287.1372.

##### 6-Acetamido-*N*-(4-nitrophenyl) Hexanamide (**5c**)

Yellow solid, yield 45%, mp: 183–186 °C, R*_f_* = 0.24 (AcOEt).

**IR**: υ_max_ (cm^−1^) 3341 (N-H), 3100–3000 (Ar-C-H), 2930 (C-H), 1697 (C-O), 1619–1597 (AR-C-C), 1560–1541 (C-N), 1503–1493 (N-O), and 1175 (C-O). **^1^H NMR** (300 MHz, DMSO-*d*_6_) δ 0.90–1.15 (*m*, 2H, CH2), 1.16–1.48 (*m*, 2H, CH2), 1.5–1.7 (*m*, 2H, CH2), 1.75 (*s*, 3H, CH3), 2.33 (*t*, 2H, J = 7.39 Hz, CH_2_), 3.04 (*dd*, 2H, *J* = 12.7, 6.6 Hz, CH_2_), 7.75 (*m*, 1H, NH-CO-CH_3_), 7.8 (*d*, 2H, J = 9.2 Hz, Ar-H), 8.15 (*d*, 2H, *J* = 9.2 Hz, Ar-H), and 10.1 (*s*, 1H, NH, Ar-NH). **^13^C NMR** (75.5 MHz, DMSO-*d*_6_) δ 22.50 (C8), 25.09 (C3), 26.27 (C4), 28.46 (C5), 36.28 (C2), 38.52 (C6), 119.05 (C10 and C14), 125.94 (C11 and C13), 142.12 (C9), 146.17 (C12), 169.89 (C7), and 173.51 (C1). **HRMS**, [M + Na]^+^: experimental = 316.1268, calculated = 316.1274.

##### 6-Acetamido-*N*-(2,4-dimethoxyphenyl) Hexanamide (**5d**)

Pink-colored solid, yield 85%, mp: 79–81 °C, R*_f_* = 0.17 (AcOEt).

**IR**: υ_max_ (cm^−1^) 3295 (N-H), 3100–3000 (Ar-C-H), 2932 (C-H), and 1633 (C=O). **^1^H NMR** (300 MHz, DMSO-*d*_6_) *δ* 0.97–1.14 (*m*, 2H, CH_2_), 1.13–1.25 (*m*, 2H, CH_2_), 1.26–1.44 (*m*, 2H, CH_2_), 1.57 (*s*, 3H, CH_3_), 2.10 (*t*, 2H, *J* = 7.3 Hz, CH_2_), 2.81 (*dd*, 2H, *J* = 12.6, 6.4 Hz, CH_2_), 3.53 (*s*, 3H, CH_3_), 3.58 (*s*, 3H, CH_3_), 6.25 (*dd*, 1H, *J* = 8.8, 2.3 Hz, Ar-H), 6.38 (*d*, 1H, *J* = 2.3 Hz, Ar-H), 7.45 (*d*, 1H, *J* = 8.7 Hz, Ar-H), 7.53 (*s*, 1H, NH-CO-CH_3_), 8.70 (*s*, 1H, Ar-NH); ^**13**^**C NMR** (75.5 MHz, DMSO-*d*_6_) δ 22.93 (C8), 25.39 (C3), 26.46 (C4), 29.32 (C5), 36.12 (C2), 38.76 (C6), 55.57–55.91 (Cs 17 and 18), 99.03 (C13), 104.24 (C11), 120.75 (C9), 124.27 (C10), 151.84 (C14), 156.99 (C12), 169.19 (C7), and 171.35 (C1). **HRMS**, [M + Na]^+^: experimental = 331.1628, calculated = 331.1634.

##### 6-(2,2,2-trifluoroacetamido) Hexanoic Acid (**3**)

White solid, yield 80%, mp: 79–82 °C, R*_f_* = 0.23 (Hexane:AcOEt = 1:1).

**IR**: υ_max_ (cm^−1^) 3300 (N-H), 3100–2300 (O-H), 2948 (C-H), 1696 (C=O), 1567 (C-N), and 1148 (C=O). ^**1**^**H NMR** (300 MHz, DMSO-*d*_6_) δ 1.25 (*m*, 2H, CH_2_), 1.45–1.55 (*m*, 4H, CH_2_, CH_2_), 2.20–2.22 (*t*, 2H, CH_2_), 3.19 (*t*, 2H, CH_2_), 9.35 (*s*, 1H, NH), and 12.05 (*s*, 1H, OH). **^13^C NMR** (75.5 MHz, DMSO-*d*_6_) δ 24.5 (C3), 26.1 (C4), 29.7 (C5), 34.0 (C2), 38.9 (C6), 116.5 (C8), 156.7 (C7), and 175.2 (C1). **HRMS**, [M + Na]^+^: experimental = 250.0661, calculated = 250.0667.

##### *N*-(2-hydroxyphenyl)-6-(2,2,2-trifluoroacetamido) Hexanamide (**6a**)

Yellow oil, yield 65%, R*_f_* = 0.57 (Hexane:AcOEt = 4:6).

**IR**: υ_max_ (cm^−1^) 3306 (N-H), 3200–3000 (O-H), 2949 (C-H), 1706 (C=O), and 1663 (Ar-C-C). **^1^H NMR** (300 MHz, DMSO-*d*_6_) δ 1.15–1.26 (*m*, 2H, CH_2_), 1.55 (*m*, 2H, CH_2_), 1.65 (*m*, 2H, CH_2_), 2.19 (*t*, 2H, *J* = 7.4 Hz, CH_2_), 2.83 (*dd*, 2H, *J* = 12.7, 6.6 Hz, CH_2_), 6.57 (*t*, 1H, *J* = 7.6 Hz, Ar-H), 6.66 (*d*, 1H, *J* = 6.8 Hz, Ar-H), 6.75 (*t*, 1H, *J* = 7.5 Hz, Ar-H), 7.49 (*d*, 1H, *J* = 7.8 Hz, Ar-H), 7.62 (*s*, 1H, NH-CO-CH_3_), 9.06 (*s*, 1H, -OH), and 9.56 (*s*, 1H, Ar-NH). **^13^C NMR** (75.5 MHz, DMSO-*d*_6_) δ 25.21 (C3), 25.92 (C4), 28.30 (C5), 35.52 (C2), 39.50 (C6), 116.5 (C8), 119.90 (C12), 120.27 (C11), 122.63 (C13), 124.91 (C10), 126.70 (C9), 148.16 (C14), 158.21 (C7), and 173.19 (C1). **HRMS**, [M + Na]^+^: experimental = 341.1083, calculated = 341.1089.

##### *N*-(4-hydroxyphenyl)-6-(2,2,2-trifluoroacetamido) Hexanamide (**6b**)

Brown solid, yield 66%, mp: 135-138 °C, R*_f_* = 0.18 (Hexane:AcOEt = 4:6).

**IR**: υ_max_ (cm^−1^) 3306 (N-H), 3000–3200 (O-H), 2949 (C-H), 1705 (C=O), and 1663 (Ar-C-C). **^1^H NMR** (300 MHz, DMSO-*d*_6_) δ 0.80–0.92 (*m*, 2H, CH_2_), 0.93–1.04 (*m*, 2H, CH_2_), 1.07–1.20 (*m*, 2H, CH_2_), 1.81 (*t*, 2H, *J* = 7.4 Hz, CH_2_), 2.60 (*dd*, 2H, *J* = 12.7, 6.6 Hz, CH_2_), 6.25 (*d*, 2H, *J* = 8.8 Hz, Ar-H), 6.94 (*d*, 2H, *J* = 8.8 Hz, Ar-H), 7.39 (*s*, 1H, NH-CO-CH_3_), 8.72 (*s*, 1H, OH), and 9.17 (*s*, 1H, Ar-NH). **^13^C NMR** (75.5 MHz, DMSO-*d*_6_) δ 25.21 (C3), 25.93 (C4), 28.42 (C5), 31.52 (C2), 39.50 (C6), 115.2 (C8), 116.10 (C12 and C11),122.25 (C10 and C14), 132.00 (C9), 154.31 (C12), 148.16 (C14), 157.21 (C7), and 172.85. **HRMS**, [M + Na]^+^: experimental = 341.1083, calculated = 341.1089.

### 3.3. Biological Evaluation

#### 3.3.1. Assessment of HDAC Inhibitory Activity in HeLa Nuclear Extracts

HDAC activity was measured with a kit (ab1438, ABCAM, Milpitas, CA, USA) to test whether the compounds inhibited the target HDACs. To obtain nuclear extracts, HeLa cell cultures were used. The HeLa cell line was cultured in high-glucose DMEM medium with phenol red, supplemented with 8% fetal bovine serum (Gibco, Waltham, MA, USA), 1% Penicillin/Streptomycin (10,000 U/mL, Gibco, Waltham, MA, USA), and 1% non-essential amino acids (Gibco, Waltham, MA, USA). Cells were maintained in an incubator at 37 °C and 5% CO_2_ atmosphere. To obtain the required number of cells for nuclear extraction, two 150 cm^2^ flasks were cultured, resulting in approximately 2 × 10^7^ cells. The cells were trypsinized with a 10% *V*/*V* trypsin solution (Gibco, Waltham, MA, USA) and subsequently centrifuged at 3000 rpm for 10 min for counting with trypan blue using an automatic counter (Mart-Counter 3000, Corning, NY, USA). Solutions for nuclear material extraction were prepared following the protocol of the commercial kit Abcam Ab156064 (ABCAM, Milpitas, CA, USA). For the lysis buffer (solution A), 10 mL was prepared with Tris-HCl (10 mM) pH 7.5, NaCl (10 mM), MgCl_2_ (15 mM), sucrose (250 mM), EGTA (0.1 mM), and NP-40 (0.5%). For the sucrose buffer (solution B), 15 mL was prepared with Tris-HCl (10 mM) pH 7.5, NaCl (10 mM), MgCl_2_ (3 mM), and sucrose (30%). For the extraction buffer (solution C), 10 mL was prepared with NaCl (420 mM), EGTA (0.1 mM), HEPES-KOH (50 mM, pH 7.5), EDTA Na₂ (0.5 mM), and glycerol (10%). For every 1 × 10^7^ of cells, 1 mL of solution A (lysis buffer) was added and vortexed for 10 s, followed by a 15 min rest in an ice bath. Then, 4 mL of solution B (sucrose buffer) was added, and the mixture was centrifuged at 1300× *g* for 10 min at 4 °C. The supernatant was discarded, and the nuclear pellet was washed with a solution of NaCl (10 mM) and Tris-HCl (10 mM) at pH 7.5. The nuclear pellet was then resuspended in 100 µL of solution C (extraction buffer), sonicated for 30 s, and placed in an ice bath for 30 min. The nuclear solution was centrifuged at 20,000× *g* for 10 min and the supernatant, containing the nuclear extract, was collected. The nuclear extracts were stored at −80 °C. The Lowry colorimetric assay was used to standardize the protein concentration of the obtained nuclear extracts. A sample of the nuclear extract and a sample of bovine serum albumin as a control were each added to 1 mL of Lowry reagent (sodium tartrate 0.1 M, sodium bicarbonate 0.1 M, CuSO_4_ 0.01 M, and 50% Folin reagent). After adding the Lowry reagent, the mixture was left to stand at room temperature for 45 min, followed by absorbance measurement at 550 nm using a spectrophotometer (Perkin Elmer model 1040, Hopkinton, MA, USA).

Prior to the experiment, a calibration curve was performed to ensure linearity. The experiment maintained an acceptable linearity with a Pearson correlation coefficient of 0.9931, yielding the linear equation Y = 18.41x + 14.48. Using concentrations of deacetylated substrate, ranging from 20 μM to 1.25 μM, while the y-axis corresponded to the fluorescence units measured in AFU.

Reactions were prepared in a 96-well plate following the specified volumes and conditions. Initially, the appropriate volume of deionized water (ddH_2_O) and 10 μL of buffer were added to each well. Subsequently, 5 μL of either acetylated or deacetylated substrate was added, depending on the experimental group.

The test compounds were prepared in solutions containing 1.06% DMSO, ensuring a final concentration of 5 μM in the wells with 0.05% DMSO. The reference inhibitor SAHA was also prepared under the same conditions, reaching a final concentration of 5 μM (DMSO 0.05%). Inhibition controls included Trichostatin A (TSA) at 1 mM, with 2 μL added to achieve a final concentration of 20 μM. The reaction was initiated by adding 4 μL of HeLa nuclear extract to each well, except for the developer control wells. The plate was incubated at 37 °C for 30 min, followed by the addition of 10 μL of developer solution. After a second incubation at 37 °C for another 30 min, fluorescence was measured using a plate reader with an excitation/emission of 350–380 nm/440–460 nm.

##### Calculation of HDAC Residual Activity

Remaining HDAC activity was estimated using the calibration curve, where the fluorescence units (AFU) obtained for each condition were converted into deacetylated substrate concentration (μM). The *AFU* value (fluorescence-test–fluorescence-blank) was substituted into the calibration equation: [Deacetylated Substrate (μM)] = 18.41∗*AFU* − 14.48, expressed by means of % of [Deacetylated Substrate (μM)]. This approach provided a direct and quantitative measure of HDAC residual activity.

#### 3.3.2. Cell Survival Assessment of Breast Cancer Cells Lines

The BC cell lines MCF-7 and MDA-MB-231 were cultured in high-glucose DMEM medium with phenol red, supplemented with 7% and 10% fetal bovine serum (Gibco, Waltham, MA, USA), respectively, 1% Penicillin/Streptomycin (10,000 U/mL, Gibco, Waltham, MA, USA), and 1% non-essential amino acids (Gibco, Waltham, MA, USA). Cell cultures were maintained in a temperature-controlled incubator at 37 °C and 5% CO_2_ atmosphere. For the 72 h assays with MDA-MB-231, 1.8 x 10³ cells/well were seeded in 100 µL of medium. After 24 h of adhesion, the cells were treated with 0, 50, 100, 250, and 500 µM of each compound and 0, 1, 8, 60, and 100 µM of SAHA, then incubated for 72 h. For subsequent IC_50_ determination assays with MDA-MB-231 and MCF-7, 1.5 × 10^3^ and 1.2 × 10^3^ cells/well were seeded, respectively. After 24 h of adhesion, the cells were treated with 0, 10, 30, 60, 100, 200, 300, 400, and 500 µM of each compound and 0, 0.5, 1, 2, 4, 8, 16, 30, 60, and 100 µM of SAHA, then incubated for 72 h.

#### 3.3.3. Cell Survival Assessment of Non-Tumorigenic Epithelial Breast Cell Line

For the culture of normal breast epithelial cells MCF-10A, DMEM-F12 medium without phenol red was used, supplemented with 5% fetal bovine serum (Gibco, Waltham, MA, USA), 1% Penicillin/Streptomycin (10,000 U/mL, Gibco, Waltham, MA, USA), 1% non-essential amino acids (Gibco, Waltham, MA, USA), 0.33% hydrocortisone, 0.2% insulin (100 U/mL), and 0.002% EGF (1 µg/µL). For the assays with MCF-10A cells, 2.5 × 10^3^ cells/well were seeded in 100 µL of medium. After 24 h of adhesion, the cells were treated with 0, 60, 100, 200, 300, 400, 500, and 600 µM of each compound (the best evaluated in cancer cells) and 0, 0.5, 1, 2, 4, 8, 16, 30, 60, and 100 µM of SAHA, and then incubated for 72 h.

#### 3.3.4. MTT Assay

The 3-(4,5-dimethylthiazol-2-yl)-2,5-diphenyl tetrazolium bromide assay was used to evaluate the effect of the designed compounds on cell viability. These compounds were initially dissolved in DMSO (dimethyl sulfoxide) as stock solutions at 150 mM. Cells were seeded in 96-well plates (NEST Biotechnology Co., Wuxi, China) at different densities, depending on the cell line used and the incubation time with the treatments, and left in 100 µL of the corresponding culture medium for 24 h to allow for adhesion. Then, the cells were treated with different concentrations of each compound, maintaining a maximum of 0.46% DMSO. After 48 or 72 h of treatment, 20 µL of MTT (5 mg/mL) in phosphate-buffered saline (PBS; Sigma) solution was added to each well and incubated again for 3 h. Subsequently, the blue formazan compound derived from the MTT was dissolved in 100 µL of DMSO. The optical density of the wells was measured at 550 nm using an ELISA reader (MultiSkan, Thermo Scientific, Waltham, MA, USA). Finally, cell viability was expressed as a percentage relative to the negative control (untreated and 0.46% DMSO).

#### 3.3.5. Statistical Analysis

Cytotoxicity data were analyzed using one-way ANOVA to evaluate differences between the treated groups and the negative control (DMSO). Multiple comparisons with the negative control were performed using Dunnett’s post hoc test. Statistical analysis was performed using GraphPad Prism 10 software (free trial, https://www.graphpad.com/demos/, accessed on 26 June 2024). For the calculation of the IC_50_s of the compounds, cytotoxicity responses were fitted to a variable slope sigmoidal equation (log(inhibitor) vs. normalized response). A *p* < 0.05 value was considered statistically significant.

## 4. Conclusions

Bioisosteric modifications of the classical pharmacophore of HDACi remain relevant in the optimization physicochemical properties, selectivity, and potency against solid tumors. Structural modifications of ZBG are those that have achieved significant benefits. In this regard, the *N*-trifluoroacetamide moiety of **6b** was found to be 1.5-fold more potent than its substituted *N*-acetamide structural analog (**5b**) in triple-negative breast cancer cells, while it was 5-fold more active in ER-positive cells. Furthermore, **6a** was ≈200-fold and **6b** was more than 50-fold less toxic than SAHA in MCF-10A cells. Consequently, these findings suggest that *N*-trifluoroacetamide derivatives are a safer option at high concentrations. Therefore, further exploration of the genomic effects related to HDAC activity is necessary for future in vivo evaluations.

## Figures and Tables

**Table 1:** ADMET values for **SAHA**, **A6**, **A7**, **B6**, and **B7**.

Comp	ADMET Properties Predictions									Score
SAHA	MW	miLogP	HBA	HBD	TPSA	nRotB	Sol	GI abs	Gp-P	80
	264.32	2.47	5	3	78.42	8	Good	High	No	
	* Lipinski	* Ghose	* Veber	* Egan	* Muegge	** PAINs	** Brenk	Mutagenic	Tumorogenic	
	0	0	0	0	0	0	2	Yes	Yes	
	Cytotoxic	Irritant	R. effects	ImmunoTox	HepatoTox	OTP	Inhibition of metabolism CYPs?			
	Yes	No	No	No	No	Yes	No			
A6	MW	miLogP	HBA	HBD	TPSA	nRotB	Sol	GI abs	Gp-P	92
	264.32	1.33	5	2	78.42	7	Good	High	No	
	* Lipinski	* Ghose	* Veber	* Egan	* Muegge	** PAINs	** Brenk	Mutagenic	Tumorogenic	
	0	0	0	0	0	0	0	No	No	
	Cytotoxic	Irritant	R. effects	ImmunoTox	HepatoTox	OTP	Inhibition of metabolism CYPs?			
	No	No	No	No	No	No	No			
A7	MW	miLogP	HBA	HBD	TPSA	nRotB	Sol	GI abs	Gp-P	89
	264.32	1.55	5	2	78.42	7	Good	High	No	
	* Lipinski	* Ghose	* Veber	* Egan	* Muegge	** PAINs	** Brenk	Mutagenic	Tumorogenic	
	0	0	0	0	0	0	1	No	No	
	Cytotoxic	Irritant	R. effects	ImmunoTox	HepatoTox	OTP	Inhibition of metabolism CYPs?			
	No	No	No	No	No	No	No			
B6	MW	miLogP	HBA	HBD	TPSA	nRotB	Sol	GI abs	Gp-P	90
	318.30	2.22	5	2	78.42	8	Good	High	No	
	* Lipinski	* Ghose	* Veber	* Egan	* Muegge	** PAINs	** Brenk	Mutagenic	Tumorogenic	
	0	0	0	0	0	0	0	No	No	
	Cytotoxic	Irritant	R. effects	ImmunoTox	HepatoTox	OTP	Inhibition of metabolism CYPs?			
	No	No	No	No	No	No	No			
B7	MW	miLogP	HBA	HBD	TPSA	nRotB	Sol	GI abs	Gp-P	88
	318.30	2.43	5	3	78.42	8	Good	High	No	
	* Lipinski	* Ghose	* Veber	* Egan	* Muegge	** PAINs	** Brenk	Mutagenic	Tumorogenic	
	0	0	0	0	0	0	1	No	No	
	Cytotoxic	Irritant	R. effects	ImmunoTox	HepatoTox	OTP	Inhibition of metabolism CYPs?			
	No	No	No	No	No	No	No			

Abbreviations: Comp (compound), MW (molecular weight), HBA (number of hydrogen bonding acceptors), HBD (number of hydrogen bonding donors), TPSA (topological polar surface area), nRotB (number of rotable bonds), Sol (solubility g/mL), GI abs (gastrointestinal absorption), Gp-P (glycoprotein P efflux), R. effects (reproductive effects), and OTP (other toxicity points). * Drug-like properties. ** Structural alerts.

**Table 2:** Results of virtual screening of best compounds selected.

		HDAC1 ^a^			HDAC6-DC2 ^b^			HDAC8 ^c^		
Ligands	ADMET Score	ΔG (Kcal/mol)	Interactions Score	% Binding	ΔG (Kcal/mol)	Interactions Score	% Binding	ΔG (Kcal/mol)	Interactions Score	% Binding
**SAHA**	80	−7.20	25.0	100	−7.51	38.4	92.9	−7.30	27.6	84.6
**A2**	91	−6.98	20.0	100	−7.57	30.2	100	−6.85	30.8	100
**A3**	91	−7.64	24.6	100	−7.86	31.6	100	−6.92	28.8	92.3
**A6**	92	−7.37	33.4	100	−7.35	30.2	92.9	−7.18	32.2	100
**A7**	89	−6.80	23.8	100	−7.67	35.2	100	−7.11	35.2	92.3
**A8**	93	−8.69	22.6	100	−12.18	18.0	78.6	−10.26	27.0	92.3
**A10**	93	−8.04	25.4	100	−7.42	26.5	85.1	−8.06	33.2	92.3
**A11**	90	−7.57	32.0	100	−7.50	34.4	100	−7.13	33.2	92.3
**A12**	88	−7.16	20.4	100	−11.24	33.4	78.6	−9.39	26.3	92.3
**A13**	85	−7.30	22.2	100	−7.52	28.8	100	−7.12	33.0	92.3
**A23**	83	−7.95	22.3	100	−8.28	31.0	100	−7.30	31.8	92.3
**B2**	92	−7.44	27.2	100	−6.66	36.8	100	−6.21	34.6	100
**B6**	90	−7.45	33.4	100	−6.63	35.8	100	−6.25	39.0	100
**B7**	88	−7.26	34.6	100	−7.17	43.1	85.7	−5.90	34.6	100
**B14**	85	−6.01	35.0	100	−6.55	39.2	100	−6.35	37.0	100
**B17**	91	−7.31	31.0	100	−6.59	35.4	100	−6.05	37.4	100
**B20**	88	−7.64	29.0	100	−7.19	38.6	100	−6.31	40.0|	92.3

For the % binding, the following residues were considered: ^a^ His140, His141, Gly149, Phe150, Asp176, His178, Phe205, Asp264, Gly301, Tyr303, and Zinc, ^b^ His463, Pro464, His573, His574, Gly582, Phe583, Asp612, His614, Phe643, Asp705, Leu712, Gly743, Tyr745, Zinc, ^c^ Tyr100, Asp101, His142, His143, Gly121, Phe152, Asp178, His180, Phe208, Asp267, Gly304, Tyr306, and Zinc.

**Table 3:** In vitro antiproliferative activity of compounds **5b**, **6a**, **6b**, and SAHA (Vorinostat) against breast cancer and healthy breast cell lines.

Cytotoxic Activity IC_50_ (µM) and Selectivity Index					
Compound	MDA-MB-231	MCF-7	MCF-10A	MCF-10A/ MDA-MB-231	MCF-10A/ MCF-7
**SAHA**	3.50 ± 0.56	4.23 ± 1.59	3.02 ± 0.32	0.86	0.71
**5b**	121.3 ± 26.65	227.7 ± 52.85	168.6 ± 20.55	1.39	0.74
**6a**	225.1 ± 17.60	253.8 ± 34.4	>600	>2.66	>2.36
**6b**	76.74 ± 8.52	45.70 ± 6.91	154.6 ± 21.6	2.01	3.38

## Data Availability

Data is contained in the paper and Supplementary Materials.

## Supplementary Materials

The following Supplementary Materials can be downloaded at: https://www.mdpi.com/article/10.3390/ph18030351/s1. Table S1. N-acetyl A and N-trifluoroacetyl B derivatives. Table S2. ADMET properties. Table S3. First and second binding mode of A6, A7, B6 and B7. Table S4. Results virtual screening of family A with HDAC1. Table S5. Results virtual screening of family A with HDAC6. Table S6. Results virtual screening of family A with HDAC8. Table S7. Results virtual screening of family B with HDAC1. Table S8. Results virtual screening of family B with HDAC6. Table S9. Results virtual screening of family B with HDAC8. Table S10. Grouping from Venn diagram of docking results HDAC1, 6 and 8 with acetylated derivatives A1–A32. Table S11. Grouping from Venn diagram of docking results HDAC1, 6 and 8 with trifluoroacetylated derivatives B1–B32. Figure S1. Re-docking result with (a) HDAC6-DC2 and (b) HDAC8 in complex with the co-crystallized ligand SAHA (cyan blue) and the pose obtained from the molecular docking of SAHA (orange). Figure S2. Venn diagram of docking results HDAC1, 6 and 8 with acetylated derivatives A1–A32. Figure S3. Venn diagram of docking results HDAC1, 6 and 8 with trifluoroacetylated derivatives B1–B32. Figures S4–S10. IR spectrum compounds **2**, **5a**, **5b**, **5c**, **5d**, **3** and **6b**. Figures S11–S24. 1H NMR spectrum and 13C NMR spectrum of compounds **2**, **5a**, **5b**, **5c**, **5d**, **3**, **6a** and **6b**. Figures S25–S32. High resolution mass spectrometry of compounds **2**, **5a**, **5b**, **5c**, **5d**, **3**, **6a** and **6b**. Figure S33. Initial viability experiment in MDA-MB-231 cells treated with (a) SAHA at 1, 8, 60 and 100 µM and (b) **2**, (c) **3**, (d) **5a**, (d) **5b**, (e) **5d**, (f) **6a**, and (g) **6b** at 50, 100, 200, and 500 µM by MTT assay. Figure S34. Optical microscopy (10×) of MDA-MB-231 cancer cells at different concentrations of (a) **2**, (b) **3**, (c) **5a**, (d) **5b**, (e) **5d**, (f) **6a**, (g) **6b** and (h) SAHA. Figure S35. Optical microscopy (10×) of MDA-MB-231 cancer cells at different concentrations of **5b**. Figure S36. Optical microscopy (10×) of MDA-MB-231 cancer cells at different concentrations of **6a**. Figure S37. Optical microscopy (10×) of MDA-MB-231 cancer cells at different concentrations of **6b**. Figure S38. Optical microscopy (10×) of MDA-MB-231 cancer cells at different concentrations of SAHA. Figure S39. Optical microscopy (10×) of MCF-7 cancer cells at different concentrations of **5b**. Figure S40. Optical microscopy (10×) of MCF-7 cancer cells at different concentrations of **6a**. Figure S41. Optical microscopy (10×) of MCF-7 cancer cells at different concentrations of **6b**. Figure S42. Optical microscopy (10×) of MCF-7 cancer cells at different concentrations of SAHA. Figure S43. Optical microscopy (10×) of MCF-10A cells at different concentrations of **5b**. Figure S44. Optical microscopy (10×) of MCF-10A cells at different concentrations of **6a**. Figure S45. Optical microscopy (10×) of MCF-10A cells at different concentrations of **6b**. Figure S46. Optical microscopy (10×) of MCF-10A cells at different concentrations of SAHA.

## Abbreviations

The following abbreviations are used in this manuscript:

ACN	acetonitrile
AcOEt	ethyl acetate
ADMET	absorption, distribution, metabolism, excretion, and toxicity
CDI	carbonyldiimidazole
DNA	deoxyribonucleic acid
DMF	dimethylformamide
HDACs	histone deacetylases
HRMS	high-resolution mass spectrometry
HDACis	histone deacetylase inhibitors
NMR	nuclear magnetic resonance
PAINs	pan-assay interference compounds
PDB	Protein Data Bank
SAHA	suberoylanilide hydroxamic acid
SAR	structure–activity relationship
TI	Therapeutic Index
ZBG	zinc-binding group
