# Supplementary material for: Computationally Guided Design, Synthesis, and Evaluation of Novel Non-Hydroxamic Histone Deacetylase Inhibitors, Based on N-Trifluoroacetamide as a Zinc-Binding Group, Against Breast Cancer
Source: Pharmaceuticals (Basel). 2025 Feb 28;18(3):351. doi: 10.3390/ph18030351 (PMC11944851; doi:10.3390/ph18030351)
Supplement: Supplementary file 1 [file pharmaceuticals-18-00351-s001.zip › pharmaceuticals-3494565-supplementary.pdf]

pharmaceuticals

Computationally Guided Design, Synthesis, and Evaluation of Novel Non-Hydroxamic Histone Deacetylase Inhibitors, Based on N-Trifluoroacetamide as a Zinc-Binding Group, Against Breast Cancer.

Gerardo Morales-Herrejón[1], Juan Benjamín García Vázquez[1,2], Cynthia Fernández Pomares[1], Norbert Bakalara[3], José Correa-Basurto[1,]* and Humberto L. Mendoza-Figueroa[1]*

[1] Laboratorio de Diseño y Desarrollo de Nuevos Fármacos e Innovación Biotecnológica (Laboratory for the Design and Development of New Drugs and Biotechnological Innovation), Escuela Superior de Medicina del Instituto Politécnico Nacional (ESM-IPN), Plan de San Luis y Salvador Díaz Mirón S/N, Casco de Santo Tomás, Ciudad de México, 11340, Mexico.

[2] Investigadoras e Investigadores por México CONAHCyT-Sección de Estudios de Posgrado e Investigación de la Escuela Superior de Medicina, Instituto Politécnico Nacional, Plan de San Luis y Salvador Díaz Mirón S/N, Casco de Santo Tomás, Ciudad de México, 11340, Mexico.

[3] Ecole Nationale Supérieure de Technologie des Biomolécules de Bordeaux (ENSTBB), Université de Bordeaux, CNRS, Bordeaux INP, CBMN, UMR 5248, F-33615 Pessac, France.

pharmaceuticals

MDPI

Article

Supporting information:

Computational section

Table S1. *N*-acetyl **A** and *N*-trifluoroacetyl **B** derivatives.

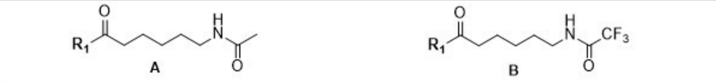

1	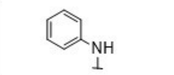	17	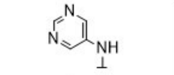
2	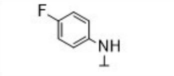	18	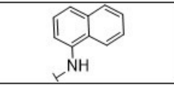
3	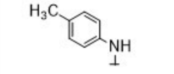	19	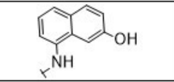
4	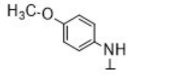	20	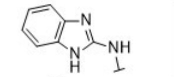
5	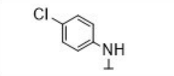	21	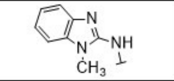
6	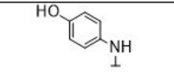	22	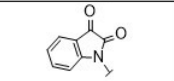
7	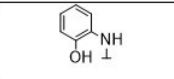	23	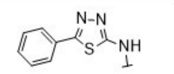
8	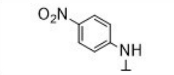	24	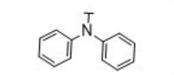
9	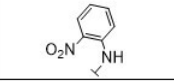	25	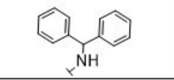
10	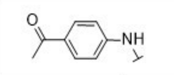	26	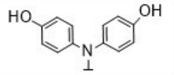
11	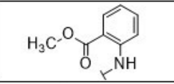	27	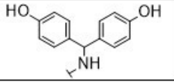
12	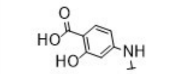	28	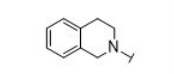
13	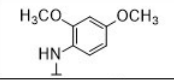	29	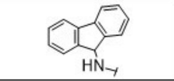
14	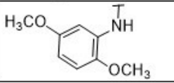	30	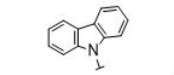
15	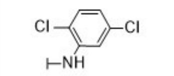	31	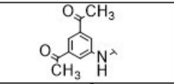
16	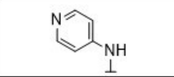	32	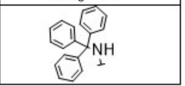

Table S2. ADMET properties, red: high probability and yellow: Medium probability.

Comp	ADME and toxicity properties									Score
**SAHA**	MW	miLogP	nON	nOHNH	TPSA	nRotB	Sol	GI abs	Gp-P	80
	264.32	2.47	5	3	78.42	8	Good	High	No	
	*Lipinski	*Ghose	*Veber	*Egan	*Muegge	**PAINs	**Brenk	Mutagenic	Tumorogenic	
	0	0	0	0	0	0	2	Yes	Yes	
	Cytotoxic	Irritant	R. effects	Immunotoxicity	Hepatotoxicity	OTP				
	Yes	No	No	No	No	Yes				
					CYP1A2	CYP2C19	CYP2C9	CYP2D6	CYP34A	
	Inhibition of metabolism CYP?				No	No	No	No	No	
**A1**	MW	miLogP	nON	nOHNH	TPSA	nRotB	Sol	GI abs	Gp-P	91
	264.32	2.47	5	3	78.42	8	Good	High	No	
	*Lipinski	*Ghose	*Veber	*Egan	*Muegge	**PAINs	**Brenk	Mutagenic	Tumorogenic	
	0	0	0	0	0	0	0	No	No	
	Cytotoxic	Irritant	R. effects	Immunotoxicity	Hepatotoxicity	OTP				
	No	No	No	No	No	No				
					CYP1A2	CYP2C19	CYP2C9	CYP2D6	CYP34A	
	Inhibition of metabolism CYP?				No	No	No	No	No	
**A2**	MW	miLogP	nON	nOHNH	TPSA	nRotB	Sol	GI abs	Gp-P	91
	266.32	1.98	4	2	58.20	7	Good	High	No	
	*Lipinski	*Ghose	*Veber	*Egan	*Muegge	**PAINs	**Brenk	Mutagenic	Tumorogenic	
	0	0	0	0	0	0	0	No	No	
	Cytotoxic	Irritant	R. effects	Immunotoxicity	Hepatotoxicity	OTP				
	No	No	No	No	No	No				
					CYP1A2	CYP2C19	CYP2C9	CYP2D6	CYP34A	
	Inhibition of metabolism CYP?				No	No	No	No	No	
**A3**	MW	miLogP	nON	nOHNH	TPSA	nRotB	Sol	GI abs	Gp-P	91
	262.35	2.26	4	2	58.20	7	Good	High	No	
	*Lipinski	*Ghose	*Veber	*Egan	*Muegge	**PAINs	**Brenk	Mutagenic	Tumorogenic	
	0	0	0	0	0	0	0	No	No	
	Cytotoxic	Irritant	R. effects	Immunotoxicity	Hepatotoxicity	OTP				
	No	No	No	No	No	No				
					CYP1A2	CYP2C19	CYP2C9	CYP2D6	CYP34A	
	Inhibition of metabolism CYP?				No	No	No	No	No	
**A4**	MW	miLogP	nON	nOHNH	TPSA	nRotB	Sol	GI abs	Gp-P	90
	278.35	1.87	5	2	67.43	7	Good	High	No	
	*Lipinski	*Ghose	*Veber	*Egan	*Muegge	**PAINs	**Brenk	Mutagenic	Tumorogenic	
	0	0	0	0	0	0	0	No	No	
	Cytotoxic	Irritant	R. effects	Immunotoxicity	Hepatotoxicity	OTP				
	No	No	No	No	No	No				
					CYP1A2	CYP2C19	CYP2C9	CYP2D6	CYP34A	
	Inhibition of metabolism CYP?				No	No	No	No	No	
	MW	miLogP	nON	nOHNH	TPSA	nRotB	Sol	GI abs	Gp-P	

A5	282.77	2.49	4	2	58.20	7	Good	High	No	87
	*Lipinski	*Ghose	*Veber	*Egan	*Muegge	**PAINs	**Brenk	Mutagenic	Tumorogenic	
	0	0	0	0	0	0	2	No	No	
	Cytotoxic	Irritant	R. effects	Immunotoxicity	Hepatotoxicity	OTP				
	No	No	No	No	No	No				
	Inhibition of metabolism CYP?				CYP1A2	CYP2C19	CYP2C9	CYP2D6	CYP34A	
					Yes	Yes	No	No	No	
A6	MW	miLogP	nON	nOHNH	TPSA	nRotB	Sol	GI abs	Gp-P	92
	264.32	1.33	5	2	78.42	7	Good	High	No	
	*Lipinski	*Ghose	*Veber	*Egan	*Muegge	**PAINs	**Brenk	Mutagenic	Tumorogenic	
	0	0	0	0	0	0	0	No	No	
	Cytotoxic	Irritant	R. effects	Immunotoxicity	Hepatotoxicity	OTP				
	No	No	No	No	No	No				
	Inhibition of metabolism CYP?				CYP1A2	CYP2C19	CYP2C9	CYP2D6	CYP34A	
					No	No	No	No	No	
A7	MW	miLogP	nON	nOHNH	TPSA	nRotB	Sol	GI abs	Gp-P	89
	264.32	1.55	5	2	78.42	7	Good	High	No	
	*Lipinski	*Ghose	*Veber	*Egan	*Muegge	**PAINs	**Brenk	Mutagenic	Tumorogenic	
	0	0	0	0	0	0	1	No	No	
	Cytotoxic	Irritant	R. effects	Immunotoxicity	Hepatotoxicity	OTP				
	No	No	No	No	No	No				
	Inhibition of metabolism CYP?				CYP1A2	CYP2C19	CYP2C9	CYP2D6	CYP34A	
					No	No	No	No	No	
A8	MW	miLogP	nON	nOHNH	TPSA	nRotB	Sol	GI abs	Gp-P	93
	293.32	1.77	7	2	104.02	8	Good	High	No	
	*Lipinski	*Ghose	*Veber	*Egan	*Muegge	**PAINs	**Brenk	Mutagenic	Tumorogenic	
	0	0	0	0	0	0	1	No	No	
	Cytotoxic	Irritant	R. effects	Immunotoxicity	Hepatotoxicity	OTP				
	No	No	No	No	No	No				
	Inhibition of metabolism CYP?				CYP1A2	CYP2C19	CYP2C9	CYP2D6	CYP34A	
					No	No	No	No	No	
A9	MW	miLogP	nON	nOHNH	TPSA	nRotB	Sol	GI abs	Gp-P	82
	293.32	1.73	7	2	104.02	8	Good	High	No	
	*Lipinski	*Ghose	*Veber	*Egan	*Muegge	**PAINs	**Brenk	Mutagenic	Tumorogenic	
	0	0	0	0	0	0	2	Yes	No	
	Cytotoxic	Irritant	R. effects	Immunotoxicity	Hepatotoxicity	OTP				
	No	No	No	No	No	No				
	Inhibition of metabolism CYP?				CYP1A2	CYP2C19	CYP2C9	CYP2D6	CYP34A	
					No	No	No	No	No	
	MW	miLogP	nON	nOHNH	TPSA	nRotB	Sol	GI abs	Gp-P	93
	276.34	1.60	5	3	75.27	8	Good	High	No	
	*Lipinski	*Ghose	*Veber	*Egan	*Muegge	**PAINs	**Brenk	Mutagenic	Tumorogenic	
	0	0	0	0	0	0	2	No	No	
	Cytotoxic	Irritant	R. effects	Immunotoxicity	Hepatotoxicity	OTP				

A10	No	No	No	No	No	No				
	Inhibition of metabolism CYP?				CYP1A2	CYP2C19	CYP2C9	CYP2D6	CYP34A	
					No	No	No	No	No	
A11	MW	miLogP	nON	nOHNH	TPSA	nRotB	Sol	GI abs	Gp-P	90
	306.36	2.12	6	2	84.50	9	Good	High	No	
	*Lipinski	*Ghose	*Veber	*Egan	*Muegge	**PAINs	**Brenk	Mutagenic	Tumorogenic	
	0	0	0	0	0	0	0	No	No	
	Cytotoxic	Irritant	R. effects	Immunotoxicity	Hepatotoxicity	OTP				
	No	No	No	No	No	No				
	Inhibition of metabolism CYP?				CYP1A2	CYP2C19	CYP2C9	CYP2D6	CYP34A	
					No	No	No	No	No	
A12	MW	miLogP	nON	nOHNH	TPSA	nRotB	Sol	GI abs	Gp-P	88
	308.33	1.72	7	4	115.72	8	Good	High	No	
	*Lipinski	*Ghose	*Veber	*Egan	*Muegge	**PAINs	**Brenk	Mutagenic	Tumorogenic	
	0	0	0	0	0	0	0	No	No	
	Cytotoxic	Irritant	R. effects	Immunotoxicity	Hepatotoxicity	OTP				
	No	No	No	No	No	No				
	Inhibition of metabolism CYP?				CYP1A2	CYP2C19	CYP2C9	CYP2D6	CYP34A	
					No	No	No	No	No	
A13	MW	miLogP	nON	nOHNH	TPSA	nRotB	Sol	GI abs	Gp-P	85
	308.38	1.85	6	2	76.66	9	Good	High	No	
	*Lipinski	*Ghose	*Veber	*Egan	*Muegge	**PAINs	**Brenk	Mutagenic	Tumorogenic	
	0	0	1	0	0	0	0	No	No	
	Cytotoxic	Irritant	R. effects	Immunotoxicity	Hepatotoxicity	OTP				
	No	No	No	Yes	No	No				
	Inhibition of metabolism CYP?				CYP1A2	CYP2C19	CYP2C9	CYP2D6	CYP34A	
					No	No	No	No	No	
A14	MW	miLogP	nON	nOHNH	TPSA	nRotB	Sol	GI abs	Gp-P	85
	308.38	1.85	6	2	76.66	9	Good	High	No	
	*Lipinski	*Ghose	*Veber	*Egan	*Muegge	**PAINs	**Brenk	Mutagenic	Tumorogenic	
	0	0	0	0	0	0	0	No	No	
	Cytotoxic	Irritant	R. effects	Immunotoxicity	Hepatotoxicity	OTP				
	No	No	No	Yes	No	No				
	Inhibition of metabolism CYP?				CYP1A2	CYP2C19	CYP2C9	CYP2D6	CYP34A	
					No	No	No	No	No	
A15	MW	miLogP	nON	nOHNH	TPSA	nRotB	Sol	GI abs	Gp-P	71
	317.22	3.10	4	2	58.20	7	Good	High	No	
	*Lipinski	*Ghose	*Veber	*Egan	*Muegge	**PAINs	**Brenk	Mutagenic	Tumorogenic	
	0	0	0	0	0	0	2	No	yes	
	Cytotoxic	Irritant	R. effects	Immunotoxicity	Hepatotoxicity	OTP				
	No	Yes	Yes	No	No	No				
	Inhibition of metabolism CYP?				CYP1A2	CYP2C19	CYP2C9	CYP2D6	CYP34A	
					Yes	Yes	Yes	No	No	

**A16**	MW	miLogP	nON	nOHNH	TPSA	nRotB	Sol	GI abs	Gp-P	86
	250.30	0.04	6	2	83.98	7	Good	High	Yes	
	*Lipinski	*Ghose	*Veber	*Egan	*Muegge	**PAINs	**Brenk	Mutagenic	Tumorogenic	
	0	0	0	0	0	0	0	No	No	
	Cytotoxic	Irritant	R. effects	Immunotoxicity	Hepatotoxicity	OTP				
	No	No	No	No	No	No				
	Inhibition of metabolism CYP?				CYP1A2	CYP2C19	CYP2C9	CYP2D6	CYP34A	
					No	No	No	No	No	
**A17**	MW	miLogP	nON	nOHNH	TPSA	nRotB	Sol	GI abs	Gp-P	92
	249.31	2.47	5	2	71.09	7	Good	High	No	
	*Lipinski	*Ghose	*Veber	*Egan	*Muegge	**PAINs	**Brenk	Mutagenic	Tumorogenic	
	0	0	0	0	0	0	1	No	No	
	Cytotoxic	Irritant	R. effects	Immunotoxicity	Hepatotoxicity	OTP				
	No	No	No	No	No	No				
	Inhibition of metabolism CYP?				CYP1A2	CYP2C19	CYP2C9	CYP2D6	CYP34A	
					No	No	No	No	No	
**A18**	MW	miLogP	nON	nOHNH	TPSA	nRotB	Sol	GI abs	Gp-P	73
	298.39	2.97	4	2	58.20	7	Good	High	No	
	*Lipinski	*Ghose	*Veber	*Egan	*Muegge	**PAINs	**Brenk	Mutagenic	Tumorogenic	
	0	0	0	0	0	0	0	Yes	Yes	
	Cytotoxic	Irritant	R. effects	Immunotoxicity	Hepatotoxicity	OTP				
	No	No	No	No	No	No				
	Inhibition of metabolism CYP?				CYP1A2	CYP2C19	CYP2C9	CYP2D6	CYP34A	
					Yes	Yes	No	Yes	Yes	
**A19**	MW	miLogP	nON	nOHNH	TPSA	nRotB	Sol	GI abs	Gp-P	75
	314.38	2.47	5	3	78.42	7	Good	High	No	
	*Lipinski	*Ghose	*Veber	*Egan	*Muegge	**PAINs	**Brenk	Mutagenic	Tumorogenic	
	0	0	0	0	0	0	2	Yes	Yes	
	Cytotoxic	Irritant	R. effects	Immunotoxicity	Hepatotoxicity	OTP				
	No	No	No	No	No	No				
	Inhibition of metabolism CYP?				CYP1A2	CYP2C19	CYP2C9	CYP2D6	CYP34A	
					No	No	No	Yes	Yes	
**A20**	MW	miLogP	nON	nOHNH	TPSA	nRotB	Sol	GI abs	Gp-P	84
	288.35	1.63	6	3	86.88	7	Good	High	Yes	
	*Lipinski	*Ghose	*Veber	*Egan	*Muegge	**PAINs	**Brenk	Mutagenic	Tumorogenic	
	0	0	0	0	0	0	2	No	No	
	Cytotoxic	Irritant	R. effects	Immunotoxicity	Hepatotoxicity	OTP				
	No	No	Yes	No	No	No				
	Inhibition of metabolism CYP?				CYP1A2	CYP2C19	CYP2C9	CYP2D6	CYP34A	
					No	No	No	No	No	
	MW	miLogP	nON	nOHNH	TPSA	nRotB	Sol	GI abs	Gp-P	
	259.35	1.97	4	1	46.92	6	Good	High	No	

A21	*Lipinski	*Ghose	*Veber	*Egan	*Muegge	**PAINs	**Brenk	Mutagenic	Tumorogenic	86
	0	0	0	0	0	0	0	No	No	
	Cytotoxic	Irritant	R. effects	Immunotoxicity	Hepatotoxicity	OTP				
	No	No	No	No	No	No				
	Inhibition of metabolism CYP?				CYP1A2	CYP2C19	CYP2C9	CYP2D6	CYP34A	
					Yes	Yes	No	Yes	No	
A22	MW	miLogP	nON	nOHNH	TPSA	nRotB	Sol	GI abs	Gp-P	85
	302.33	1.47	6	1	85.25	6	Good	High	No	
	*Lipinski	*Ghose	*Veber	*Egan	*Muegge	**PAINs	**Brenk	Mutagenic	Tumorogenic	
	0	0	0	0	0	0	1	No	No	
	Cytotoxic	Irritant	R. effects	Immunotoxicity	Hepatotoxicity	OTP				
	No	No	No	No	No	No				
	Inhibition of metabolism CYP?				CYP1A2	CYP2C19	CYP2C9	CYP2D6	CYP34A	
					No	Yes	No	No	Yes	
A23	MW	miLogP	nON	nOHNH	TPSA	nRotB	Sol	GI abs	Gp-P	83
	332.43	2.45	6	2	83.98	8	Good	High	Yes	
	*Lipinski	*Ghose	*Veber	*Egan	*Muegge	**PAINs	**Brenk	Mutagenic	Tumorogenic	
	0	0	0	0	0	0	1	No	No	
	Cytotoxic	Irritant	R. effects	Immunotoxicity	Hepatotoxicity	OTP				
	No	No	No	No	No	No				
	Inhibition of metabolism CYP?				CYP1A2	CYP2C19	CYP2C9	CYP2D6	CYP34A	
					No	Yes	No	No	Yes	
A24	MW	miLogP	nON	nOHNH	TPSA	nRotB	Sol	GI abs	Gp-P	81
	324.42	3.76	4	1	49.41	8	Good	High	No	
	*Lipinski	*Ghose	*Veber	*Egan	*Muegge	**PAINs	**Brenk	Mutagenic	Tumorogenic	
	0	0	0	0	0	0	0	No	No	
	Cytotoxic	Irritant	R. effects	Immunotoxicity	Hepatotoxicity	OTP				
	No	No	No	No	No	No				
	Inhibition of metabolism CYP?				CYP1A2	CYP2C19	CYP2C9	CYP2D6	CYP34A	
					Yes	Yes	Yes	Yes	Yes	
A25	MW	miLogP	nON	nOHNH	TPSA	nRotB	Sol	GI abs	Gp-P	82
	338.44	3.01	4	2	58.20	9	Good	High	Yes	
	*Lipinski	*Ghose	*Veber	*Egan	*Muegge	**PAINs	**Brenk	Mutagenic	Tumorogenic	
	0	0	1	0	0	0	0	No	No	
	Cytotoxic	Irritant	R. effects	Immunotoxicity	Hepatotoxicity	OTP				
	No	No	No	No	No	No				
	Inhibition of metabolism CYP?				CYP1A2	CYP2C19	CYP2C9	CYP2D6	CYP34A	
					Yes	Yes	Yes	Yes	Yes	
A26	MW	miLogP	nON	nOHNH	TPSA	nRotB	Sol	GI abs	Gp-P	77
	264.32	1.33	5	2	78.42	7	Good	Low	No	
	*Lipinski	*Ghose	*Veber	*Egan	*Muegge	**PAINs	**Brenk	Mutagenic	Tumorogenic	
	0	0	1	0	0	0	0	No	No	
	Cytotoxic	Irritant	R. effects	Immunotoxicity	Hepatotoxicity	OTP				
	No	No	No	No	No	No				

	Inhibition of metabolism CYP?				CYP1A2	CYP2C19	CYP2C9	CYP2D6	CYP34A	
					Yes	Yes	Yes	Yes	Yes	
A27	MW	miLogP	nON	nOHNH	TPSA	nRotB	Sol	GI abs	Gp-P	74
	398.50	3.41	6	2	76.66	11	Good	Hight	No	
	*Lipinski	*Ghose	*Veber	*Egan	*Muegge	**PAINs	**Brenk	Mutagenic	Tumorogenic	
	0	0	1	0	0	0	0	No	No	
	Cytotoxic	Irritant	R. effects	Immunotoxicity	Hepatotoxicity	OTP				
	No	No	Yes	No	No	No				
	Inhibition of metabolism CYP?				CYP1A2	CYP2C19	CYP2C9	CYP2D6	CYP34A	
					No	Yes	Yes	Yes	Yes	
A28	MW	miLogP	nON	nOHNH	TPSA	nRotB	Sol	GI abs	Gp-P	85
	288.39	49.41	4	1	49.41	6	Good	High	Yes	
	*Lipinski	*Ghose	*Veber	*Egan	*Muegge	**PAINs	**Brenk	Mutagenic	Tumorogenic	
	0	0	0	0	0	0	0	No	No	
	Cytotoxic	Irritant	R. effects	Immunotoxicity	Hepatotoxicity	OTP				
	No	No	No	No	No	No				
	Inhibition of metabolism CYP?				CYP1A2	CYP2C19	CYP2C9	CYP2D6	CYP34A	
					No	No	No	Yes	No	
A29	MW	miLogP	nON	nOHNH	TPSA	nRotB	Sol	GI abs	Gp-P	71
	336.44	3.23	4	2	58.20	7	Good	High	Yes	
	*Lipinski	*Ghose	*Veber	*Egan	*Muegge	**PAINs	**Brenk	Mutagenic	Tumorogenic	
	0	0	0	0	0	0	0	Yes	Yes	
	Cytotoxic	Irritant	R. effects	Immunotoxicity	Hepatotoxicity	OTP				
	No	No	No	No	No	No				
	Inhibition of metabolism CYP?				CYP1A2	CYP2C19	CYP2C9	CYP2D6	CYP34A	
					No	Yes	Yes	Yes	Yes	
A30	MW	miLogP	nON	nOHNH	TPSA	nRotB	Sol	GI abs	Gp-P	78
	322.41	4.11	4	1	51.10	6	Good	High	No	
	*Lipinski	*Ghose	*Veber	*Egan	*Muegge	**PAINs	**Brenk	Mutagenic	Tumorogenic	
	0	0	0	0	0	0	2	No	No	
	Cytotoxic	Irritant	R. effects	Immunotoxicity	Hepatotoxicity	OTP				
	No	No	No	No	No	No				
	Inhibition of metabolism CYP?				CYP1A2	CYP2C19	CYP2C9	CYP2D6	CYP34A	
					Yes	Yes	Yes	Yes	Yes	
A31	MW	miLogP	nON	nOHNH	TPSA	nRotB	Sol	GI abs	Gp-P	81
	364.40	2.08	8	2	110.81	11	Good	High	No	
	*Lipinski	*Ghose	*Veber	*Egan	*Muegge	**PAINs	**Brenk	Mutagenic	Tumorogenic	
	0	0	1	0	0	0	1	No	No	
	Cytotoxic	Irritant	R. effects	Immunotoxicity	Hepatotoxicity	OTP				
	No	Yes	No	No	No	No				
	Inhibition of metabolism CYP?				CYP1A2	CYP2C19	CYP2C9	CYP2D6	CYP34A	
					No	No	No	No	No	

**A32**	MW	miLogP	nON	nOHNH	TPSA	nRotB	Sol	GI abs	Gp-P	78
	414.55	4.96	4	2	58.20	10	Good	High	Yes	
	*Lipinski	*Ghose	*Veber	*Egan	*Muegge	**PAINs	**Brenk	Mutagenic	Tumorogenic	
	0	0	1	0	0	0	0	No	No	
	Cytotoxic	Irritant	R. effects	Immunotoxicity	Hepatotoxicity	OTP				
	No	No	No	No	No	No				
	Inhibition of metabolism CYP?				CYP1A2	CYP2C19	CYP2C9	CYP2D6	CYP34A	
					No	Yes	Yes	Yes	Yes	
**B1**	MW	miLogP	nON	nOHNH	TPSA	nRotB	Sol	GI abs	Gp-P	91
	264.32	2.47	5	3	78.42	8	Good	High	No	
	*Lipinski	*Ghose	*Veber	*Egan	*Muegge	**PAINs	**Brenk	Mutagenic	Tumorogenic	
	0	0	1	0	0	0	0	No	No	
	Cytotoxic	Irritant	R. effects	Immunotoxicity	Hepatotoxicity	OTP				
	No	No	No	Y	No	No				
	Inhibition of metabolism CYP?				CYP1A2	CYP2C19	CYP2C9	CYP2D6	CYP34A	
					No	No	No	No	No	
**B2**	MW	miLogP	nON	nOHNH	TPSA	nRotB	Sol	GI abs	Gp-P	92
	320.29	2.86	6	2	58.20	8	Good	High	No	
	*Lipinski	*Ghose	*Veber	*Egan	*Muegge	**PAINs	**Brenk	Mutagenic	Tumorogenic	
	0	0	0	0	0	0	0	No	No	
	Cytotoxic	Irritant	R. effects	Immunotoxicity	Hepatotoxicity	OTP				
	No	No	No	No	No	No				
	Inhibition of metabolism CYP?				CYP1A2	CYP2C19	CYP2C9	CYP2D6	CYP34A	
					Yes	No	No	No	No	
**B3**	MW	miLogP	nON	nOHNH	TPSA	nRotB	Sol	GI abs	Gp-P	84
	316.32	3.15	4	2	58.20	8	Good	High	No	
	*Lipinski	*Ghose	*Veber	*Egan	*Muegge	**PAINs	**Brenk	Mutagenic	Tumorogenic	
	0	0	0	0	0	0	2	No	yes	
	Cytotoxic	Irritant	R. effects	Immunotoxicity	Hepatotoxicity	OTP				
	No	No	No	No	No	No				
	Inhibition of metabolism CYP?				CYP1A2	CYP2C19	CYP2C9	CYP2D6	CYP34A	
					No	Yes	No	No	No	
**B4**	MW	miLogP	nON	nOHNH	TPSA	nRotB	Sol	GI abs	Gp-P	86
	320.29	2.86	4	2	58.20	8	Good	High	Yes	
	*Lipinski	*Ghose	*Veber	*Egan	*Muegge	**PAINs	**Brenk	Mutagenic	Tumorogenic	
	0	0	1	0	0	0	0	No	No	
	Cytotoxic	Irritant	R. effects	Immunotoxicity	Hepatotoxicity	OTP				
	No	No	No	No	No	No				
	Inhibition of metabolism CYP?				CYP1A2	CYP2C19	CYP2C9	CYP2D6	CYP34A	
					No	No	No	No	No	
	MW	miLogP	nON	nOHNH	TPSA	nRotB	Sol	GI abs	Gp-P	86
	336.74	3.38	4	2	58.20	8	Good	High	No	
	*Lipinski	*Ghose	*Veber	*Egan	*Muegge	**PAINs	**Brenk	Mutagenic	Tumorogenic	
	0	0	0	0	0	0	1	No	No	

**B5**	Cytotoxic	Irritant	R. effects	Immunotoxicity	Hepatotoxicity	OTP				
	No	No	No	No	No	No				
					CYP1A2	CYP2C19	CYP2C9	CYP2D6	CYP34A	
	Inhibition of metabolism CYP?				Yes	Yes	Yes	No	No	
**B6**	MW	miLogP	nON	nOHNH	TPSA	nRotB	Sol	GI abs	Gp-P	90
	318.30	2.22	5	2	78.42	8	Good	High	No	
	*Lipinski	*Ghose	*Veber	*Egan	*Muegge	**PAINs	**Brenk	Mutagenic	Tumorogenic	
	0	0	0	0	0	0	0	No	No	
	Cytotoxic	Irritant	R. effects	Immunotoxicity	Hepatotoxicity	OTP				
	No	No	No	No	No	No				
					CYP1A2	CYP2C19	CYP2C9	CYP2D6	CYP34A	
	Inhibition of metabolism CYP?				No	No	No	No	No	
**B7**	MW	miLogP	nON	nOHNH	TPSA	nRotB	Sol	GI abs	Gp-P	88
	318.30	2.43	5	3	78.42	8	Good	High	No	
	*Lipinski	*Ghose	*Veber	*Egan	*Muegge	**PAINs	**Brenk	Mutagenic	Tumorogenic	
	0	0	0	0	0	0	1	No	No	
	Cytotoxic	Irritant	R. effects	Immunotoxicity	Hepatotoxicity	OTP				
	No	No	No	No	No	No				
					CYP1A2	CYP2C19	CYP2C9	CYP2D6	CYP34A	
	Inhibition of metabolism CYP?				No	No	No	No	No	
**B8**	MW	miLogP	nON	nOHNH	TPSA	nRotB	Sol	GI abs	Gp-P	81
	347.29	2.66	7	2	104.02	9	Good	High	No	
	*Lipinski	*Ghose	*Veber	*Egan	*Muegge	**PAINs	**Brenk	Mutagenic	Tumorogenic	
	0	0	1	0	0	0	1	Yes	No	
	Cytotoxic	Irritant	R. effects	Immunotoxicity	Hepatotoxicity	OTP				
	No	No	No	No	No	No				
					CYP1A2	CYP2C19	CYP2C9	CYP2D6	CYP34A	
	Inhibition of metabolism CYP?				No	Yes	No	No	No	
**B9**	MW	miLogP	nON	nOHNH	TPSA	nRotB	Sol	GI abs	Gp-P	82
	347.29	2.61	7	2	104.02	9	Good	High	No	
	*Lipinski	*Ghose	*Veber	*Egan	*Muegge	**PAINs	**Brenk	Mutagenic	Tumorogenic	
	0	0	1	0	0	0	1	Yes	No	
	Cytotoxic	Irritant	R. effects	Immunotoxicity	Hepatotoxicity	OTP				
	No	No	No	No	No	No				
					CYP1A2	CYP2C19	CYP2C9	CYP2D6	CYP34A	
	Inhibition of metabolism CYP?				No	Yes	No	No	No	
**B10**	MW	miLogP	nON	nOHNH	TPSA	nRotB	Sol	GI abs	Gp-P	84
	344.33	2.60	5	2	75.27	9	Good	High	No	
	*Lipinski	*Ghose	*Veber	*Egan	*Muegge	**PAINs	**Brenk	Mutagenic	Tumorogenic	
	0	0	1	0	0	0	0	No	Yes	
	Cytotoxic	Irritant	R. effects	Immunotoxicity	Hepatotoxicity	OTP				
	No	No	No	No	No	No				
					CYP1A2	CYP2C19	CYP2C9	CYP2D6	CYP34A	
	Inhibition of metabolism CYP?				No	No	No	No	No	

**B11**	MW	miLogP	nON	nOHNH	TPSA	nRotB	Sol	GI abs	Gp-P	85
	360.33	2.07	7	2	84.50	10	Good	Low	No	
	*Lipinski	*Ghose	*Veber	*Egan	*Muegge	**PAINs	**Brenk	Mutagenic	Tumorogenic	
	0	0	1	0	0	0	0	No	No	
	Cytotoxic	Irritant	R. effects	Immunotoxicity	Hepatotoxicity	OTP				
	No	No	No	No	No	No				
	Inhibition of metabolism CYP?				CYP1A2	CYP2C19	CYP2C9	CYP2D6	CYP34A	
					No	No	No	No	No	
**B12**	MW	miLogP	nON	nOHNH	TPSA	nRotB	Sol	GI abs	Gp-P	87
	362.30	2.61	7	4	115.72	9	Good	Low	No	
	*Lipinski	*Ghose	*Veber	*Egan	*Muegge	**PAINs	**Brenk	Mutagenic	Tumorogenic	
	0	0	1	0	0	0	0	No	No	
	Cytotoxic	Irritant	R. effects	Immunotoxicity	Hepatotoxicity	OTP				
	No	No	No	No	No	No				
	Inhibition of metabolism CYP?				CYP1A2	CYP2C19	CYP2C9	CYP2D6	CYP34A	
					No	No	No	No	No	
**B13**	MW	miLogP	nON	nOHNH	TPSA	nRotB	Sol	GI abs	Gp-P	85
	362.35	2.74	6	2	76.66	10	Good	Low	No	
	*Lipinski	*Ghose	*Veber	*Egan	*Muegge	**PAINs	**Brenk	Mutagenic	Tumorogenic	
	0	0	1	0	0	0	0	No	No	
	Cytotoxic	Irritant	R. effects	Immunotoxicity	Hepatotoxicity	OTP				
	No	No	No	Yes	No	No				
	Inhibition of metabolism CYP?				CYP1A2	CYP2C19	CYP2C9	CYP2D6	CYP34A	
					No	No	No	No	No	
**B14**	MW	miLogP	nON	nOHNH	TPSA	nRotB	Sol	GI abs	Gp-P	85
	362.35	2.74	6	2	76.66	10	Good	Low	No	
	*Lipinski	*Ghose	*Veber	*Egan	*Muegge	**PAINs	**Brenk	Mutagenic	Tumorogenic	
	0	0	0	0	0	0	0	No	No	
	Cytotoxic	Irritant	R. effects	Immunotoxicity	Hepatotoxicity	OTP				
	No	No	No	Yes	No	No				
	Inhibition of metabolism CYP?				CYP1A2	CYP2C19	CYP2C9	CYP2D6	CYP34A	
					No	No	No	No	No	
**B15**	MW	miLogP	nON	nOHNH	TPSA	nRotB	Sol	GI abs	Gp-P	71
	278.35	1.87	5	2	67.43	7	Good	Low	No	
	*Lipinski	*Ghose	*Veber	*Egan	*Muegge	**PAINs	**Brenk	Mutagenic	Tumorogenic	
	0	0	0	0	0	0	0	No	Yes	
	Cytotoxic	Irritant	R. effects	Immunotoxicity	Hepatotoxicity	OTP				
	No	Yes	Yes	No	No	No				
	Inhibition of metabolism CYP?				CYP1A2	CYP2C19	CYP2C9	CYP2D6	CYP34A	
					Yes	Yes	Yes	No	No	
	MW	miLogP	nON	nOHNH	TPSA	nRotB	Sol	GI abs	Gp-P	
	304.27	0.92	6	2	83.98	8	Good	Low	Yes	

**B16**	*Lipinski	*Ghose	*Veber	*Egan	*Muegge	**PAINs	**Brenk	Mutagenic	Tumorogenic	87
	0	0	0	0	0	0	2	No	No	
	Cytotoxic	Irritant	R. effects	Immunotoxicity	Hepatotoxicity	OTP				
	No	No	No	No	No	No				
	Inhibition of metabolism CYP?				CYP1A2	CYP2C19	CYP2C9	CYP2D6	CYP34A	
					No	No	No	No	No	
**B17**	MW	miLogP	nON	nOHNH	TPSA	nRotB	Sol	GI abs	Gp-P	91
	303.28	1.41	5	2	71.09	8	Good	Low	No	
	*Lipinski	*Ghose	*Veber	*Egan	*Muegge	**PAINs	**Brenk	Mutagenic	Tumorogenic	
	0	0	0	0	0	0	0	No	No	
	Cytotoxic	Irritant	R. effects	Immunotoxicity	Hepatotoxicity	OTP				
	No	No	No	No	No	No				
	Inhibition of metabolism CYP?				CYP1A2	CYP2C19	CYP2C9	CYP2D6	CYP34A	
					No	No	No	No	No	
**B18**	MW	miLogP	nON	nOHNH	TPSA	nRotB	Sol	GI abs	Gp-P	72
	352.26	3.86	4	2	58.20	8	Good	Low	No	
	*Lipinski	*Ghose	*Veber	*Egan	*Muegge	**PAINs	**Brenk	Mutagenic	Tumorogenic	
	0	0	0	0	0	0	0	No	No	
	Cytotoxic	Irritant	R. effects	Immunotoxicity	Hepatotoxicity	OTP				
	No	No	No	No	No	No				
	Inhibition of metabolism CYP?				CYP1A2	CYP2C19	CYP2C9	CYP2D6	CYP34A	
					Yes	Yes	Yes	Yes	Yes	
**B19**	MW	miLogP	nON	nOHNH	TPSA	nRotB	Sol	GI abs	Gp-P	88
	368.33	3.36	5	3	78.42	8	Good	Low	No	
	*Lipinski	*Ghose	*Veber	*Egan	*Muegge	**PAINs	**Brenk	Mutagenic	Tumorogenic	
	0	0	0	0	0	0	0	No	No	
	Cytotoxic	Irritant	R. effects	Immunotoxicity	Hepatotoxicity	OTP				
	No	No	No	No	No	No				
	Inhibition of metabolism CYP?				CYP1A2	CYP2C19	CYP2C9	CYP2D6	CYP34A	
					No	Yes	Yes	Yes	Yes	
**B20**	MW	miLogP	nON	nOHNH	TPSA	nRotB	Sol	GI abs	Gp-P	88
	342.32	2.51	6	3	86.88	8	Good	Low	Yes	
	*Lipinski	*Ghose	*Veber	*Egan	*Muegge	**PAINs	**Brenk	Mutagenic	Tumorogenic	
	0	0	0	0	0	0	0	Yes	No	
	Cytotoxic	Irritant	R. effects	Immunotoxicity	Hepatotoxicity	OTP				
	No	No	No	No	No	No				
	Inhibition of metabolism CYP?				CYP1A2	CYP2C19	CYP2C9	CYP2D6	CYP34A	
					Yes	No	No	No	No	
**B21**	MW	miLogP	nON	nOHNH	TPSA	nRotB	Sol	GI abs	Gp-P	86
	313.32	2.86	4	1	46.92	8	Good	Good	No	
	*Lipinski	*Ghose	*Veber	*Egan	*Muegge	**PAINs	**Brenk	Mutagenic	Tumorogenic	
	0	0	0	0	0	0	0	No	No	
	Cytotoxic	Irritant	R. effects	Immunotoxicity	Hepatotoxicity	OTP				
	No	No	No	No	No	No				

	Inhibition of metabolism CYP?				CYP1A2	CYP2C19	CYP2C9	CYP2D6	CYP34A	
					Yes	Yes	No	Yes	No	
**B22**	MW	miLogP	nON	nOHNH	TPSA	nRotB	Sol	GI abs	Gp-P	84
	356.30	2.36	6	1	85.25	7	Good	Good	No	
	*Lipinski	*Ghose	*Veber	*Egan	*Muegge	**PAINs	**Brenk	Mutagenic	Tumorogenic	
	0	0	0	0	0	0	0	No	No	
	Cytotoxic	Irritant	R. effects	Immunotoxicity	Hepatotoxicity	OTP				
	No	No	No	No	No	No				
	Inhibition of metabolism CYP?				CYP1A2	CYP2C19	CYP2C9	CYP2D6	CYP34A	
					No	No	No	No	No	
**B23**	MW	miLogP	nON	nOHNH	TPSA	nRotB	Sol	GI abs	Gp-P	82
	386.40	3.33	6	2	83.98	9	Good	High	No	
	*Lipinski	*Ghose	*Veber	*Egan	*Muegge	**PAINs	**Brenk	Mutagenic	Tumorogenic	
	0	0	1	0	0	0	0	No	No	
	Cytotoxic	Irritant	R. effects	Immunotoxicity	Hepatotoxicity	OTP				
	No	No	No	No	No	No				
	Inhibition of metabolism CYP?				CYP1A2	CYP2C19	CYP2C9	CYP2D6	CYP34A	
					Yes	Yes	Yeas	No	Yes	
**B24**	MW	miLogP	nON	nOHNH	TPSA	nRotB	Sol	GI abs	Gp-P	75
	378.39	4.64	4	1	49.41	9	Good	Low	No	
	*Lipinski	*Ghose	*Veber	*Egan	*Muegge	**PAINs	**Brenk	Mutagenic	Tumorogenic	
	0	1	1	0	0	0	0	No	No	
	Cytotoxic	Irritant	R. effects	Immunotoxicity	Hepatotoxicity	OTP				
	No	No	No	Yes	No	No				
	Inhibition of metabolism CYP?				CYP1A2	CYP2C19	CYP2C9	CYP2D6	CYP34A	
					No	No	No	No	No	
**B25**	MW	miLogP	nON	nOHNH	TPSA	nRotB	Sol	GI abs	Gp-P	80
	392.42	4.18	4	2	58.20	10	Good	Low	Yes	
	*Lipinski	*Ghose	*Veber	*Egan	*Muegge	**PAINs	**Brenk	Mutagenic	Tumorogenic	
	0	0	1	0	0	0	0	No	No	
	Cytotoxic	Irritant	R. effects	Immunotoxicity	Hepatotoxicity	OTP				
	No	No	No	Yes	No	No				
	Inhibition of metabolism CYP?				CYP1A2	CYP2C19	CYP2C9	CYP2D6	CYP34A	
					Yes	Yes	Yes	Yes	Yes	
**B26**	MW	miLogP	nON	nOHNH	TPSA	nRotB	Sol	GI abs	Gp-P	73
	438.45	4.75	6	1	67.88	11	Good	Low	No	
	*Lipinski	*Ghose	*Veber	*Egan	*Muegge	**PAINs	**Brenk	Mutagenic	Tumorogenic	
	1	0	1	0	0	0	2	No	No	
	Cytotoxic	Irritant	R. effects	Immunotoxicity	Hepatotoxicity	OTP				
	No	No	No	No	No	No				
	Inhibition of metabolism CYP?				CYP1A2	CYP2C19	CYP2C9	CYP2D6	CYP34A	
					No	Yes	Yes	Yes	Yes	

B27	MW	miLogP	nON	nOHNH	TPSA	nRotB	Sol	GI abs	Gp-P	74
	452.47	4.29	6	2	76.66	12	Good	Low	Yes	
	*Lipinski	*Ghose	*Veber	*Egan	*Muegge	**PAINs	**Brenk	Mutagenic	Tumorogenic	
	1	0	1	0	0	0	0	No	No	
	Cytotoxic	Irritant	R. effects	Immunotoxicity	Hepatotoxicity	OTP				
	No	No	Yes	No	No	No				
	Inhibition of metabolism CYP?				CYP1A2	CYP2C19	CYP2C9	CYP2D6	CYP34A	
					No	Yes	Yes	Yes	Yes	
B28	MW	miLogP	nON	nOHNH	TPSA	nRotB	Sol	GI abs	Gp-P	85
	342.36	2.64	4	1	49.41	7	Good	High	No	
	*Lipinski	*Ghose	*Veber	*Egan	*Muegge	**PAINs	**Brenk	Mutagenic	Tumorogenic	
	0	0	0	0	0	0	0	No	No	
	Cytotoxic	Irritant	R. effects	Immunotoxicity	Hepatotoxicity	OTP				
	No	No	No	No	No	No				
	Inhibition of metabolism CYP?				CYP1A2	CYP2C19	CYP2C9	CYP2D6	CYP34A	
					No	Yes	No	Yes	No	
B29	MW	miLogP	nON	nOHNH	TPSA	nRotB	Sol	GI abs	Gp-P	72
	390.40	4.11	4	2	58.20	8	Good	Low	Yes	
	*Lipinski	*Ghose	*Veber	*Egan	*Muegge	**PAINs	**Brenk	Mutagenic	Tumorogenic	
	0	0	0	0	0	0	0	No	No	
	Cytotoxic	Irritant	R. effects	Immunotoxicity	Hepatotoxicity	OTP				
	No	No	No	No	No	No				
	Inhibition of metabolism CYP?				CYP1A2	CYP2C19	CYP2C9	CYP2D6	CYP34A	
					No	Yes	Yes	Yes	Yes	
B30	MW	miLogP	nON	nOHNH	TPSA	nRotB	Sol	GI abs	Gp-P	76
	376.38	5	4	1	51.10	7	Little	Very Low	No	
	*Lipinski	*Ghose	*Veber	*Egan	*Muegge	**PAINs	**Brenk	Mutagenic	Tumorogenic	
	0	1	0	1	0	0	0	No	No	
	Cytotoxic	Irritant	R. effects	Immunotoxicity	Hepatotoxicity	OTP				
	No	No	No	No	No	No				
	Inhibition of metabolism CYP?				CYP1A2	CYP2C19	CYP2C9	CYP2D6	CYP34A	
					Yes	Yes	Yes	Yes	Yes	
B31	MW	miLogP	nON	nOHNH	TPSA	nRotB	Sol	GI abs	Gp-P	73
	418.37	2.97	8	2	110.81	12	Little	Very low	No	
	*Lipinski	*Ghose	*Veber	*Egan	*Muegge	**PAINs	**Brenk	Mutagenic	Tumorogenic	
	0	0	0	0	0	0	2	No	No	
	Cytotoxic	Irritant	R. effects	Immunotoxicity	Hepatotoxicity	OTP				
	No	No	No	No	No	No				
	Inhibition of metabolism CYP?				CYP1A2	CYP2C19	CYP2C9	CYP2D6	CYP34A	
					No	Yes	No	No	No	

Abbreviations: Comp (Compound), MW (molecular weight), nON (Number of Hydrogen bonding acceptors), nOHNH (number of Hydrogen bonding donors), TPSA (topological polar surface area), nRotB (number of rotable bonds), Sol (solubility g/mL), GI abs (Gastrointestinal absorption),Gp-P (Glycoprotein P efflux), R. effects (Reproductive effects) and OTP (Other toxicity points). * Drug like properties. ** Structural alerts.

Table S3. First and second binding mode of **A6, A7**, **B6** and **B7**.

Compounds	HDAC1	HDAC8
	ΔG (Kcal/mol)	ΔG (Kcal/mol)
A6-1	-7.37	-6.64
A6-2	-7.02	-5.97
A7-1	-6.80	-6.80
A7-2	-8.05	No fount
B6-1	-6.38	-6.25
B6-2	-7.45	-5.51
B7-1	-6.08	-5.87
B7-2	-7.26	-5.90

Table S4. Results virtual screening of family A with HDAC1.

Compounds	ADMET*100	ΔG (kcal/mol)	10*(-ΔG)	Interactions	% Binding	∑ Docking	*Global
SAHA	80	-7.20	72	25	100	197	**277**
A1	91	-7.22	72.2	20.2	100	192.4	**283.4**
A2	91	-6.98	69.8	20	100	189.8	**280.8**
A3	91	-7.64	76.4	24.6	100	201	**292**
A4	90	-6.96	69.6	24	100	193.6	**283.6**
A5	87	-7.34	73.4	25.6	100	199	**286**
A6	92	-7.37	73.7	33.4	100	207.1	**299.1**
A7	89	-6.8	68	23.8	100	191.8	**280.8**
A8	93	-8.69	86.9	22.6	100	209.5	**302.5**
A9	82	-7	70	28.4	100	198.4	**280.4**
A10	93	-8.04	80.4	25.4	100	205.8	**298.8**
A11	90	-7.57	75.7	32	100	207.7	**297.7**
A12	88	-7.16	71.6	20.45	100	192.0	**280.0**
A13	85	-7.30	73	22.2	100	195.2	**280.2**
A14	85	-7.31	73.1	25.8	100	198.9	**283.9**
A15	71	X	X	X	X	X	**X**
A16	86	-6.96	69.6	22.8	100	192.4	**278.4**
A17	92	-7.18	71.8	23.6	100	195.4	**287.4**
A18	73	--	--	--	--	--	**0**
A19	75	--	--	--	--	--	**--**
A20	84	-8.84	88.4	27.8	100	216.2	**300.2**
A21	86	-7.6	76	20.2	100	196.2	**282.2**

A22	85	-8.25	82.5	23.6	100	206.1	**291.1**
A23	83	-7.95	79.5	22.2	100	201.7	**284.7**
A24	81	-7.58	75.8	30.4	100	206.2	**287.2**
A25	82	-7.16	71.6	28	100	199.6	**281.6**
A26	77	--	--	--	--	--	**0**
A27	74	--	--	--	--	--	**0**
A28	85	-8.02	80.2	22.2	100	202.4	**287.4**
A29	71	--	--	--	--	--	**0**
A30	78	--	--	--	--	--	**0**
A31	79	--	--	--	--	--	**0**
A32	78	--	--	--	--	--	**0**

Global Score = ADMET Score + [∑ Docking Score = (% Binding) + (Interactions) + (10(-ΔG) Docking)].

Table S5. Results virtual screening of family A with HDAC6.

Compounds	ADMET*100	ΔG (kcal/mol)	10*(-ΔG)	Interactions	% Binding	∑ Docking	*Global
SAHA	80	-7.51	75.1	38.4	92.85	206.35	**286.35**
A1	91	-6.54	65.4	29.2	100	194.60	**285.60**
A2	91	-7.57	75.7	30.2	100	205.90	**296.90**
A3	91	-7.86	78.6	31.6	100	210.20	**301.20**
A4	90	-7.55	75.5	27.8	100	203.30	**293.30**
A5	87	-7.69	76.9	30.2	100	207.10	**294.10**
A6	92	-7.35	73.5	30.2	92.85	196.55	**288.55**
A7	89	-7.67	76.7	35.2	100	211.90	**300.90**
A8	93	-12.18	121.8	18	78.57	218.37	**311.37**
A9	82	-7.42	74.2	34.4	100	208.60	**290.60**
A10	93	-8.41	84.1	26.4	85.71	196.26	**289.26**
A11	90	-7.50	75	34.4	100	209.40	**299.40**
A12	88	-11.24	112.4	33.4	78.57	224.37	**312.37**
A13	85	-7.52	75.2	28.8	100	204	**289**
A14	85	-7.50	75	31.2	92.85	199.05	**284.05**
A15	71	--	--	--	--	--	**0**
A16	86	-7.22	72.2	30	100	202.20	**288.2**
A17	92	-7.42	74.2	28.2	92.85	195.25	**287.25**

A18	73	--	--	--	--	--	**0**
A19	75	--	--	--	--	--	**0**
A20	84	-7.81	78.1	32.6	85.71	196.41	**280.41**
A21	86	-7.52	75.2	29.6	92.85	197.65	**283.65**
A22	85	-7.95	79.5	32.2	100	211.70	**296.70**
A23	83	-8.28	82.8	31	100	213.8	**296.80**
A24	81	-7.95	79.5	23.4	92.85	195.75	**276.75**
A25	82	-7.81	78.1	23.6	100	201.7	**283.70**
A26	77	--	--	--	--	--	**0**
A27	74	--	--	--	--	--	**0**
A28	85	-8.74	87.4	29.7	85.71	202.81	**287.81**
A29	71	--	--	--	--	--	**0**
A30	78	--	--	--	--	--	**0**
A31	81	-8.28	82.8	36.2	85.71	204.71	**285.71**
A32	78	--	--	--	--	--	**0**

Global Score = ADMET Score + [∑Docking Score = (% Binding) + (Interactions) + (10(-ΔG) Docking)].

Table S6. Results virtual screening of family A with HDAC8.

Compounds	ADMET*100	ΔG (kcal/mol)	10*(-ΔG)	Interactions	% Bindimg	∑ Docking	*Global
SAHA	80	-7.30	73	27.6	84.61	185.21	**265.21**
A1	91	-6.97	69.7	26.7	92.3	188.70	**279.70**
A2	91	-6.85	68.5	30.8	100	199.30	**290.30**
A3	91	-6.92	69.2	28.8	92.30	190.30	**281.30**
A4	90	-6.53	65.3	26.2	92.30	183.80	**273.80**
A5	87	-6.84	68.4	27.4	92.30	188.10	**275.10**
A6	92	-7.18	71.8	32.2	100	204	**296**
A7	89	-7.11	71.1	35.2	92.30	198.60	**287.60**
A8	93	-10.26	102.6	27	92.30	221.90	**314.90**
A9	82	-7.33	73.3	34	84.61	191.91	**273.91**
A10	93	-8.06	80.6	33.2	92.30	206.10	**299.10**
A11	90	-7.13	71.3	33.2	92.30	196.80	**286.80**
A12	88	-9.39	93.9	26.25	92.30	212.45	**300.45**
A13	85	-7.12	71.2	33	92.30	196.50	**281.50**
A14	85	-7.17	71.7	37.8	84.61	194.11	**279.11**

A15	71	--	--	--	--	--	**0**
A16	86	-6.93	69.3	29.4	92.30	191.00	**277.00**
A17	92	-6.90	69	30.8	100	199.80	**291.80**
A18	73	--	--	--	--	--	**0**
A19	75	--	--	--	--	--	**0**
A20	84	-7.06	70.6	31	84.61	186.21	**270.21**
A21	86	-6.67	66.7	21.2	92.30	180.20	**266.20**
A22	85	-6.90	69	23	84.61	176.61	**261.61**
A23	83	-7.30	73	31.8	92.30	197.10	**280.10**
A24	81	-7.18	71.8	18.8	92.30	182.90	**263.90**
A25	82	-7.29	72.9	33.2	100	206.10	**288.10**
A26	77	--	--	--	--	--	**0**
A27	74	--	--	--	--	--	**0**
A28	85	-7.38	73.8	18.8	92.30	184.90	**269.90**
A29	71	--	--	--	--	--	**0**
A30	78	--	--	--	--	--	**0**
A31	81	-6.93	69.3	21.2	92.30	182.80	**263.80**
A32	78	--	--	--	--	--	**0**

Global Score = ADMET Score + [∑Docking Score = (% Binding) + (Interactions) + (10(-ΔG) Docking)].

Table S7. Results virtual screening of family B with HDAC1.

Compounds	ADMET*100	ΔG (kcal/mol)	10*(-ΔG)	Interactions	% Binding	∑ Docking	*Global
SAHA	80	-7.22	72.2	25	80	177.2	**257.2**
B1	91	-6.40	64	33.8	100	197.8	**288.8**
B2	92	-7.44	74.4	27.2	100	201.6	**293.6**
B3	84	-6.76	67.6	35.4	100	203	**287**
B4	86	-6.61	66.1	30	100	196.1	**282.1**
B5	86	-7	70	30	100	200	**286**
B6	90	-7.45	74.5	33.4	100	207.9	**297.9**
B7	88	-7.26	72.6	34.6	100	207.2	**295.2**
B8	81	-8.08	80.8	29.6	73.3	183.7	**264.7**
B9	82	-11.67	116.7	37.6	100	254.3	**336.3**
B10	84	-7.35	73.5	35	100	208.5	**292.5**
B11	85	-6.50	65	28	100	193	**278**

B12	87	-6.85	68.5	20.4	73.3	162.2	**249.2**
B13	85	-5.85	58.5	33.8	100	192.3	**277.3**
B14	85	-6.01	60.1	35	100	195.1	**280.1**
B15	71	--	--	--	--	--	**0**
B16	87	-7.01	70.1	42.8	100	212.9	**299.9**
B17	91	-7.31	73.1	31	100	204.1	**295.1**
B18	72	--	--	--	--	--	**0**
B19	88	-6.90	69	45.6	100	214.6	**302.6**
B20	88	-7.64	76.4	29	100	205.4	**293.4**
B21	86	-6.94	69.4	28.6	100	198	**284**
B22	84	-7.22	72.2	45.6	100	217.8	**301.8**
B23	82	-7.77	77.7	37.05	100	214.7	**296.75**
B24	75	-6.93	69.3	33.4	100	202.7	**277.7**
B25	80	-6.84	68.4	29.4	100	197.8	**277.8**
B26	73	--	--	--	--	--	**0**
B27	74	--	--	--	--	--	**0**
B28	85	-7.33	73.3	31.4	100	204.7	**289.7**
B29	72	--	--	--	--	--	**0**
B30	76	--	--	--	--	--	**0**
B31	73	--	--	--	--	--	**0**
B32	71	--	--	--	--	--	**0**

Global Score = ADMET Score + [∑Docking Score = (% Binding) + (Interactions) + (10(-ΔG) Docking)].

Table S8. Results virtual screening of family B with HDAC6.

Compounds	ADMET*100	ΔG (kcal/mol)	10*(-ΔG)	Interactions	% Binding	∑ Docking	*Global
SAHA	80	-7.51	75.1	38.4	92.85	206.35	**286.35**
B1	91	-6.77	67.7	28.4	100	196.10	**287.10**
B2	92	-6.66	66.6	36.8	100	203.40	**295.40**
B3	84	-6.94	69.4	34	100	203.40	**287.40**
B4	86	-6.52	65.2	38.4	100	203.60	**289.60**
B5	86	-6.75	67.5	34.9	85.71	188.11	**274.11**
B6	90	-6.63	66.3	35.8	100	202.10	**292.10**
B7	88	-7.17	71.7	43.05	85.71	200.46	**288.46**
B8	81	-12.03	120.3	25.4	64.28	209.98	**290.98**
B9	82	-7.43	74.3	32.6	92.85	199.75	**281.75**

B10	84	-7.61	76.1	27	100	203.10	**287.10**
B11	85	-6.64	66.4	35.2	85.71	187.31	**272.31**
B12	87	-10.42	104.2	25.8	85.71	215.71	**302.71**
B13	85	-6.33	63.3	41.4	100	204.70	**289.70**
B14	85	-6.55	65.5	39.2	100	204.70	**289.70**
B15	71	--	--	--	--	--	**0**
B16	87	-6.38	63.8	34.6	92.85	191.25	**278.25**
B17	91	-6.59	65.9	35.4	100	201.30	**292.3**
B18	72	--	--	--	--	--	**0**
B19	88	--	--	--	--	--	**0**
B20	88	-7.19	71.9	38.6	100	210.50	**298.5**
B21	86	-6.86	68.6	36	92.85	197.45	**283.45**
B22	84	-7.02	70.2	41.4	92.85	204.45	**288.45**
B23	82	-7.19	71.9	35.4	100	207.3	**289.30**
B24	75	-7.21	72.1	33.8	85.71	191.61	**266.61**
B25	80	-7.43	74.3	35.7	85.71	195.71	**275.71**
B26	73		--	--	--	--	**0**
B27	74	--	--	--	--	--	**0**
B28	85	-6.82	68.2	28.2	85.71	182.11	**267.11**
B29	72	--	--	--	--	--	**0**
B30	76	--	--	--	--	--	**0**
B31	73	-6.58	65.8	45	85.71	196.51	**269.5143**
B32	71	--	--	--	--	--	**0**

Global Score = ADMET Score + [∑Docking Score = (% Binding) + (Interactions) + (10(-ΔG) Docking)].

Table S9. Results virtual screening of family B with HDAC8.

Compounds	ADMET*100	ΔG (kcal/mol)	10*(-ΔG)	Interactions	% Binding	∑ docking	*Global
SAHA	80	-7.3	73	27.6	84.6	185.21	**265.21**
B1	91	-6.19	61.9	38.2	92.3	192.40	**283.40**
B2	92	-6.21	62.1	34.6	100	196.70	**288.70**
B3	84	-6.26	62.6	25.2	92.30	180.10	**264.10**
B4	86	-6.3	63	34.4	92.30	189.70	**275.70**
B5	86	-6.5	65	37.4	100	202.40	**288.40**
B6	90	-6.25	62.5	39	100	201.50	**291.50**
B7	88	-5.9	59	34.6	100	193.60	**281.60**

B8	81	-9.73	97.3	31	92.30	220.60	**301.60**
B9	82	-6.02	60.2	40	84.61	184.81	**266.81**
B10	84	-7.57	75.7	30.2	92.30	198.20	**282.20**
B11	85	-6.29	62.9	40.2	92.30	195.40	**280.40**
B12	87	-9.1	91	31.1	92.30	214.40	**301.40**
B13	85	-6.01	60.1	38.2	92.30	190.60	**275.60**
B14	85	-6.35	63.5	37	100	200.50	**285.50**
B15	71	--	--	--	--	--	**0**
B16	87	-6.14	61.4	38.2	100	199.60	**286.60**
B17	91	-6.05	60.5	37.4	100	197.90	**288.90**
B18	72	--	--	--	--	--	**0**
B19	88	-6.88	68.8	42.8	92.30	203.90	**291.90**
B20	88	-6.31	63.1	40	92.30	195.40	**283.40**
B21	86	-5.97	59.7	35.2	92.30	187.20	**273.20**
B22	84	-6.27	62.7	30.6	84.61	177.91	**261.91**
B23	82	-7.1	71	29.8	92.30	193.10	**275.10**
B24	75	--	--	--	--	--	**0**
B25	80	-5.94	59.4	38	92.30	189.70	**269.70**
B26	73	--	--	--	--	--	**0**
B27	74	--	--	--	--	--	**0**
B28	85	-6.5	65	26.0	92.30	183.35	**268.35**
B29	72	--	--	--	--	--	**0**
B30	76	--	--	--	--	--	**0**
B31	73	--	--	--	--	--	**0**
B32	71	--	--	--	--	--	**0**

Global Score = ADMET Score + [∑Docking Score = (% Binding) + (Interactions) + (10(-ΔG) Docking)].

Table S10. Grouping from Venn diagram of docking results HDAC1, 6 and 8 with acetylated derivatives A1 - A32.

no.	Groups	Compounds
1	HDAC1	A1, A14, A16, A20, A21, A24 and A28
2	HDAC6	A16
3	HDAC8	None
4	HDAC1 *U* HDAC6	A4, A5, A9 and A22
5	HDAC1 *U* HDAC8	A17 and A25
6	HDAC6 *U* HDAC8	None
7	**HDAC1 *U* HDAC6 *U* HDAC8**	A2, A3, A6, A7, A8, A10, A11, A12, A13 and A23

Table S11. Grouping from Venn diagram of docking results HDAC1, 6 and 8 with trifluoroacetylated derivatives B1 - B32.

no.	Groups	Compounds
1	HDAC1	B3, B9, B21 and B28
2	HDAC6	B13
3	HDAC8	B11
4	HDAC1 *U* HDAC6	B4, B22 and B23
5	HDAC1 *U* HDAC8	B1, B5, B10, B16 and B19
6	HDAC6 *U* HDAC8	B8 and B12
7	**HDAC1 *U* HDAC6 *U* HDAC8**	B2, B6, B7, B14, B17 and B20

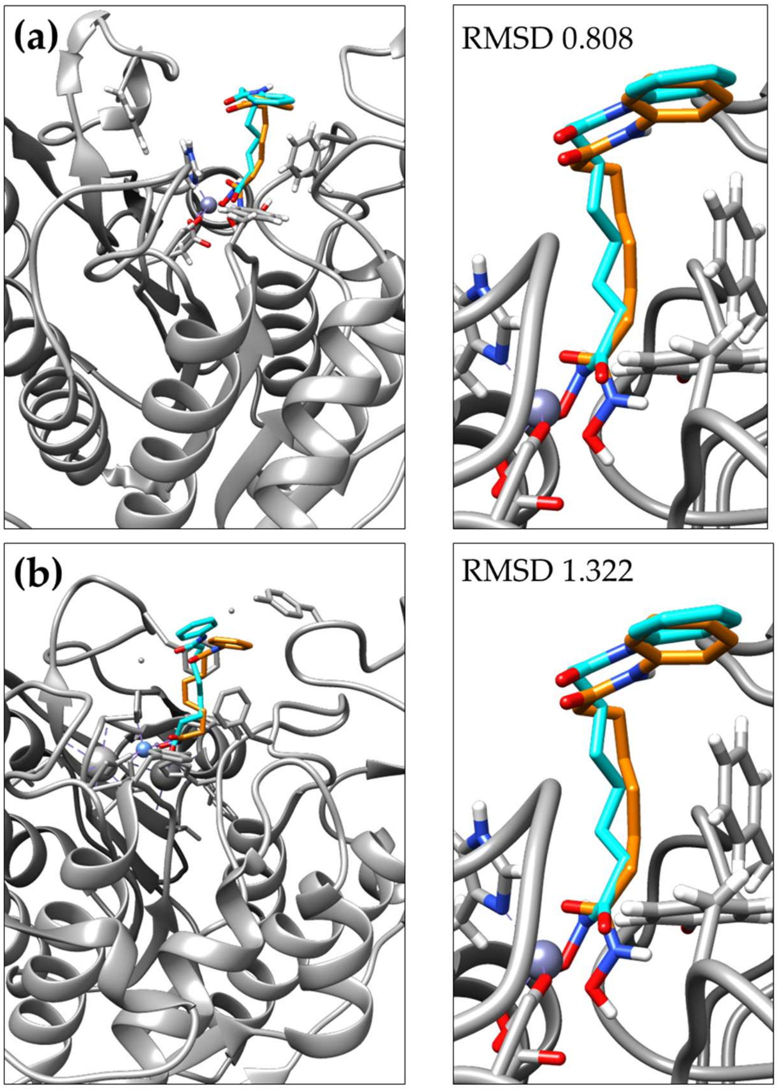

Figure S1. Redocking result with (a) HDAC6-DC2 and (b) HDAC8 in complex with the co-crystallized ligand SAHA (cyan blue) and the pose obtained from the molecular docking of SAHA (orange).

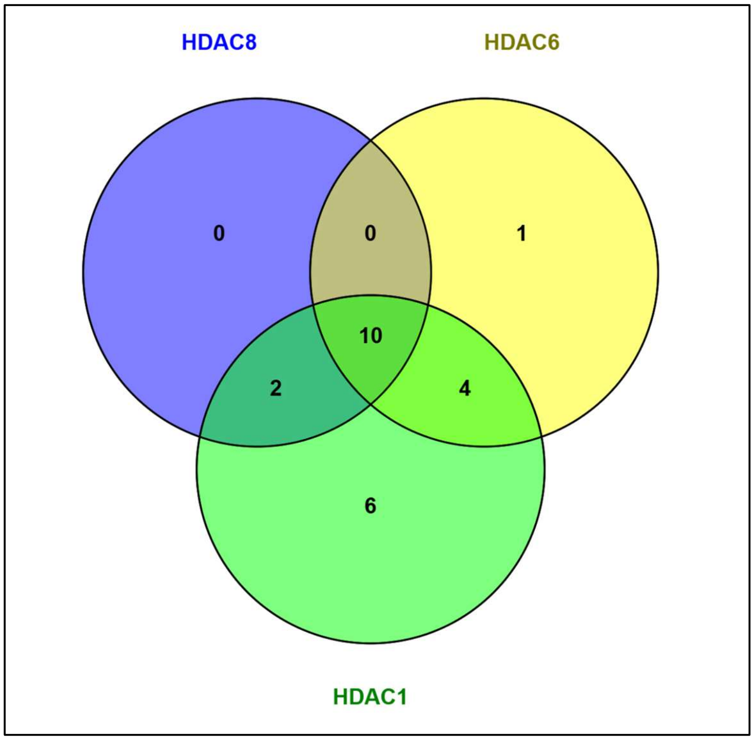

Figure S2. Venn diagram of docking results HDAC1, 6 and 8 with acetylated derivatives A1 - A32.

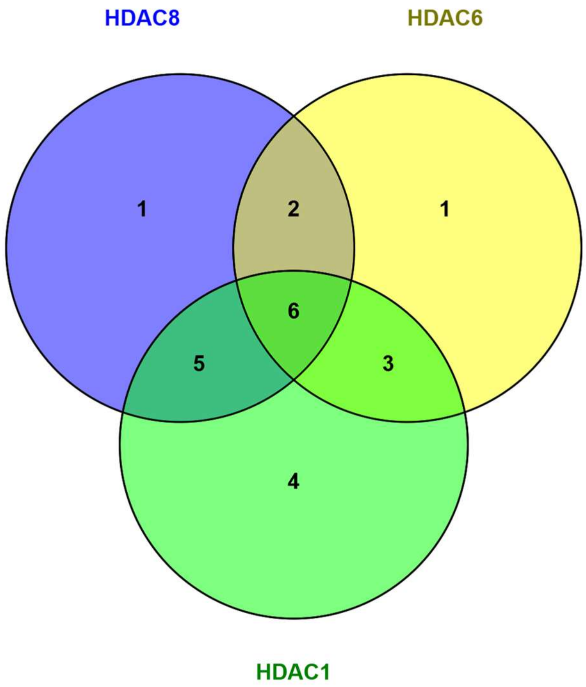

Figure S3. Venn diagram of docking results HDAC1, 6 and 8 with trifluoroacetylated derivatives B1 - B32.

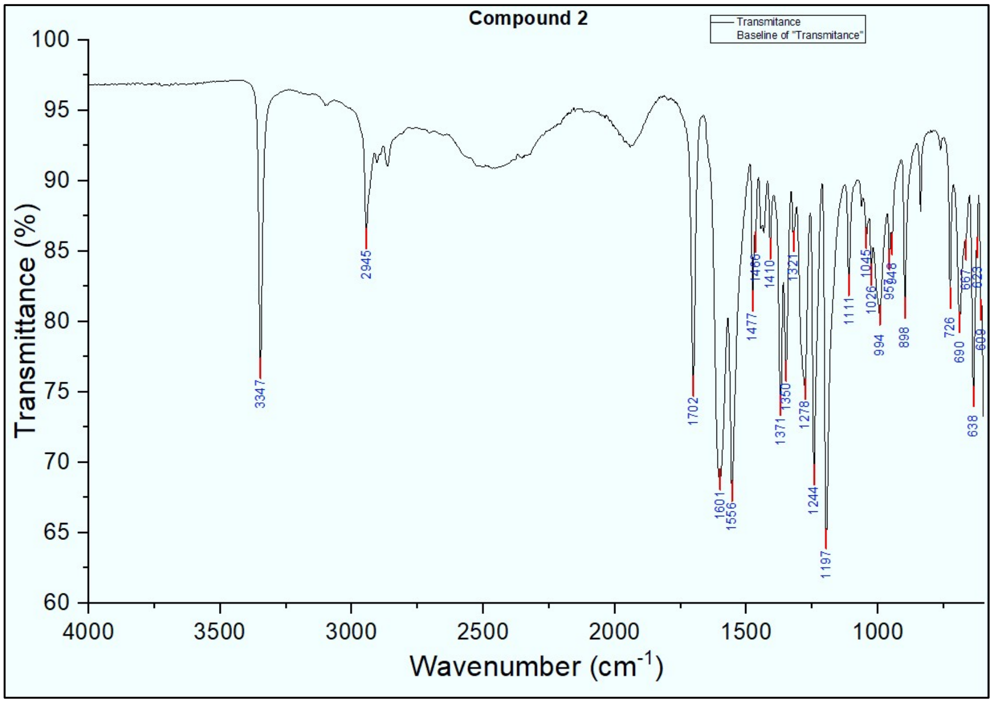

Figure S4. IR spectrum compound **2**.

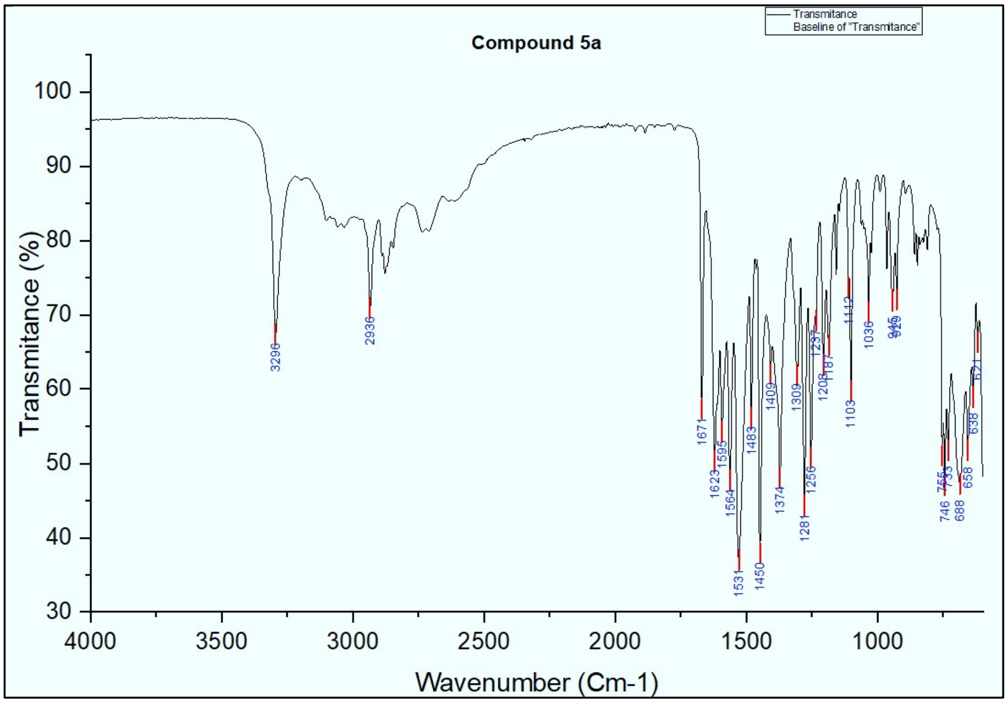

Figure S5. IR Spectrum compound **5a**.

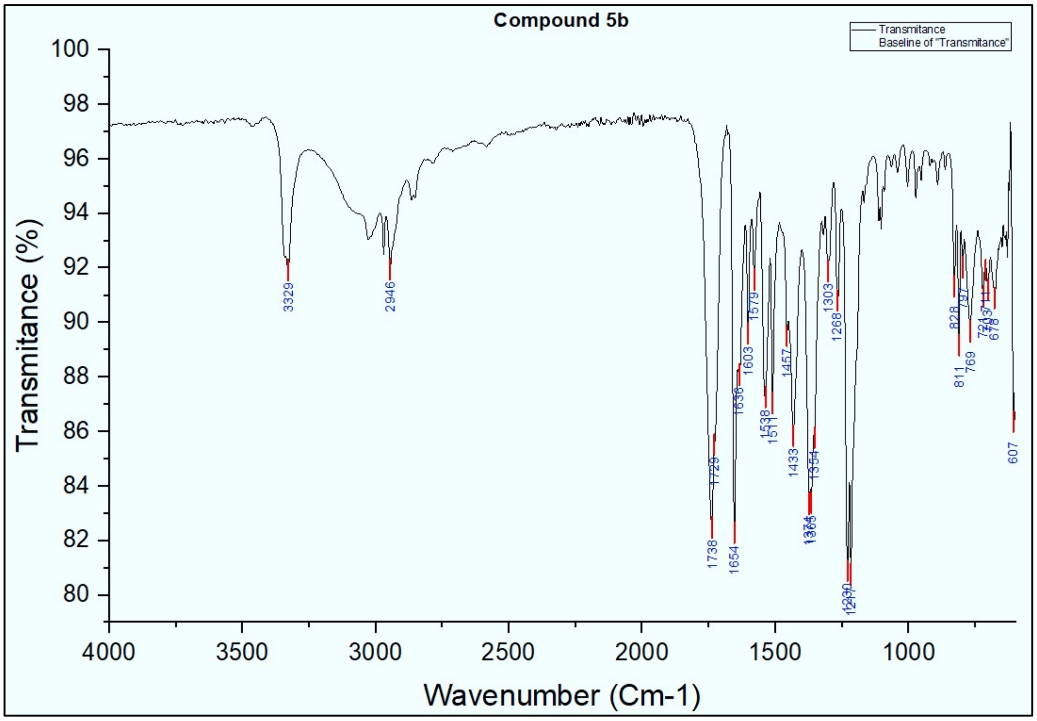

Figure S6. IR spectrum compound **5b**.

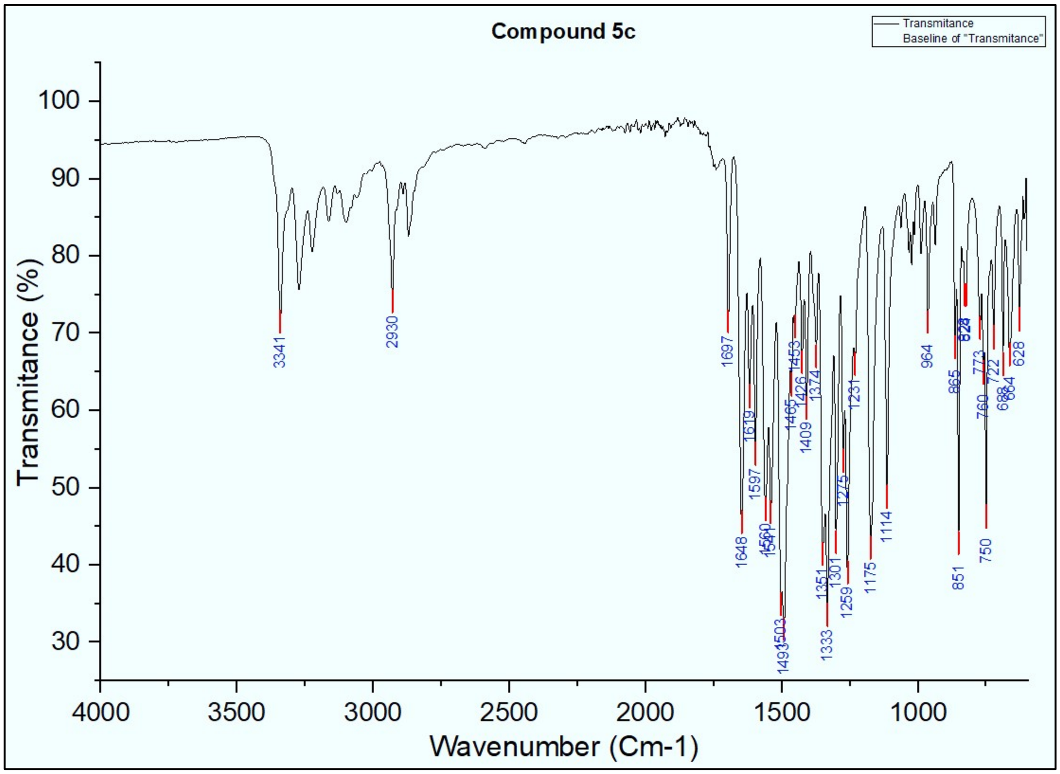

Figure S7. IR spectrum compound **5c**.

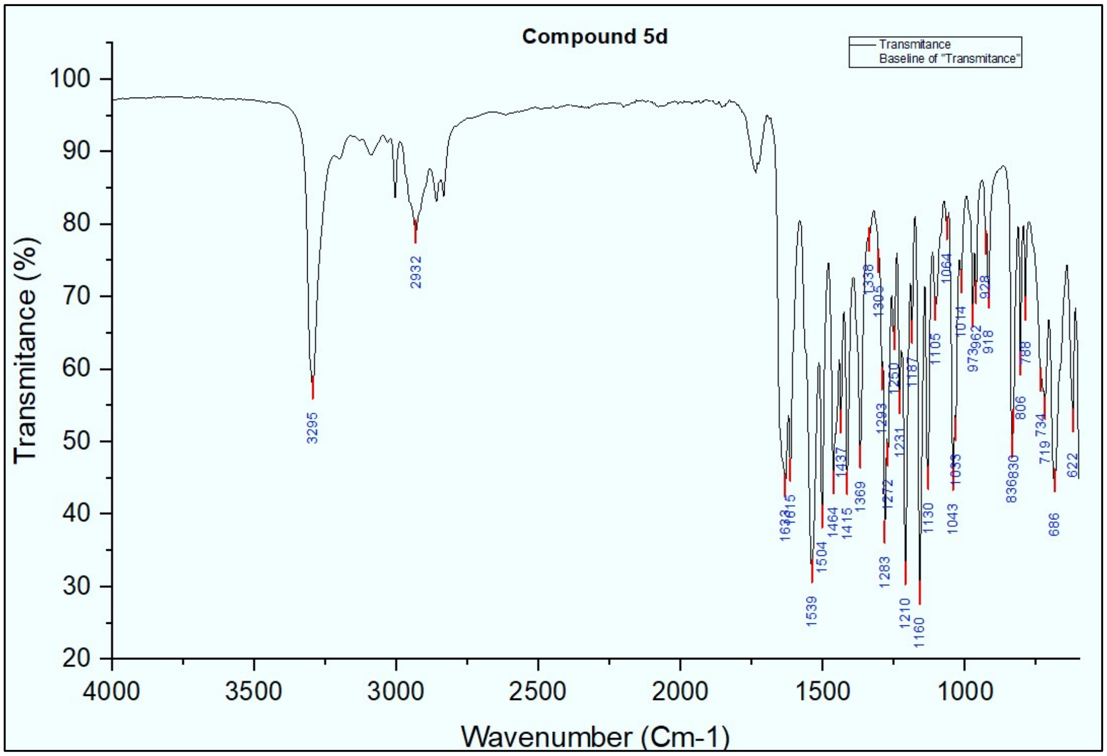

Figure S8. IR spectrum compound **5d**.

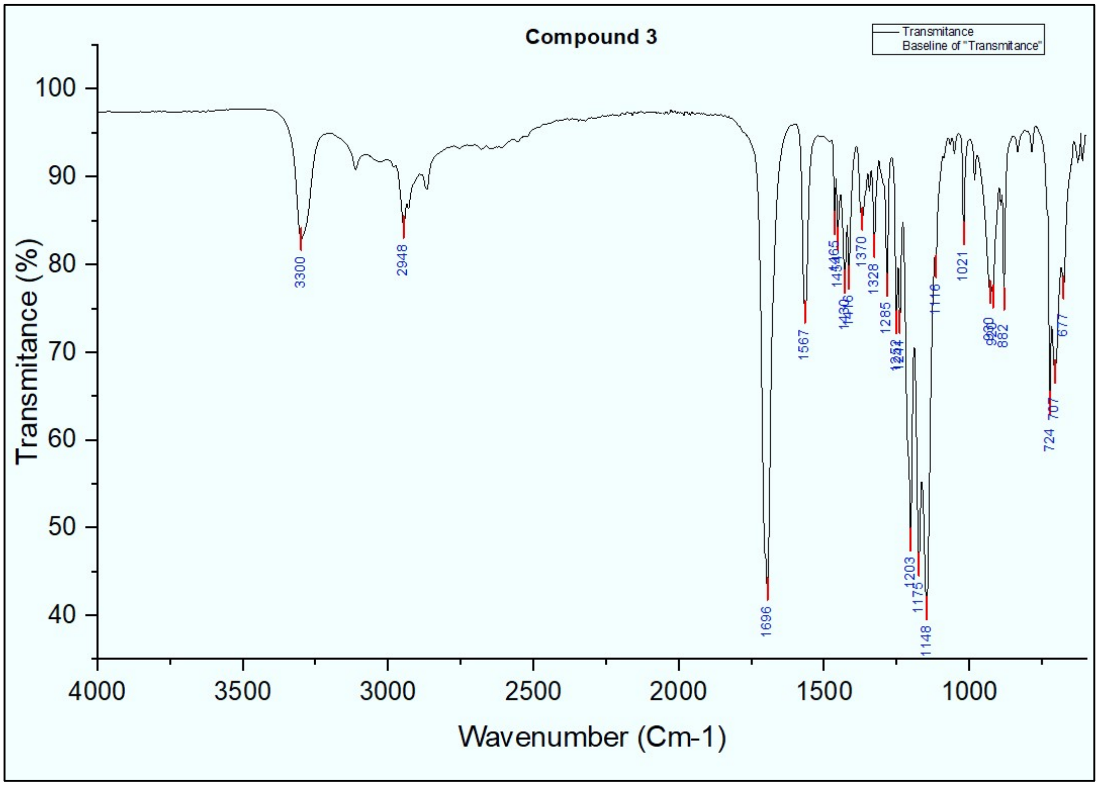

Figure S9. IR spectrum compound **3.**

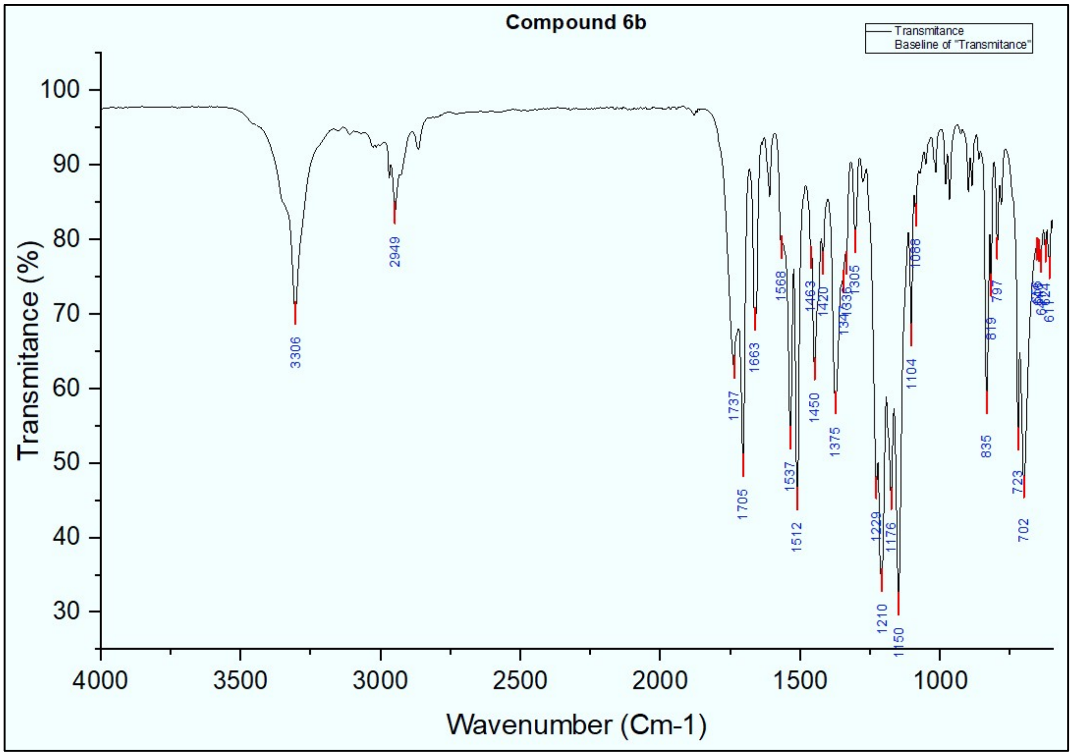

Figure S10. IR spectrum compound **6b**.

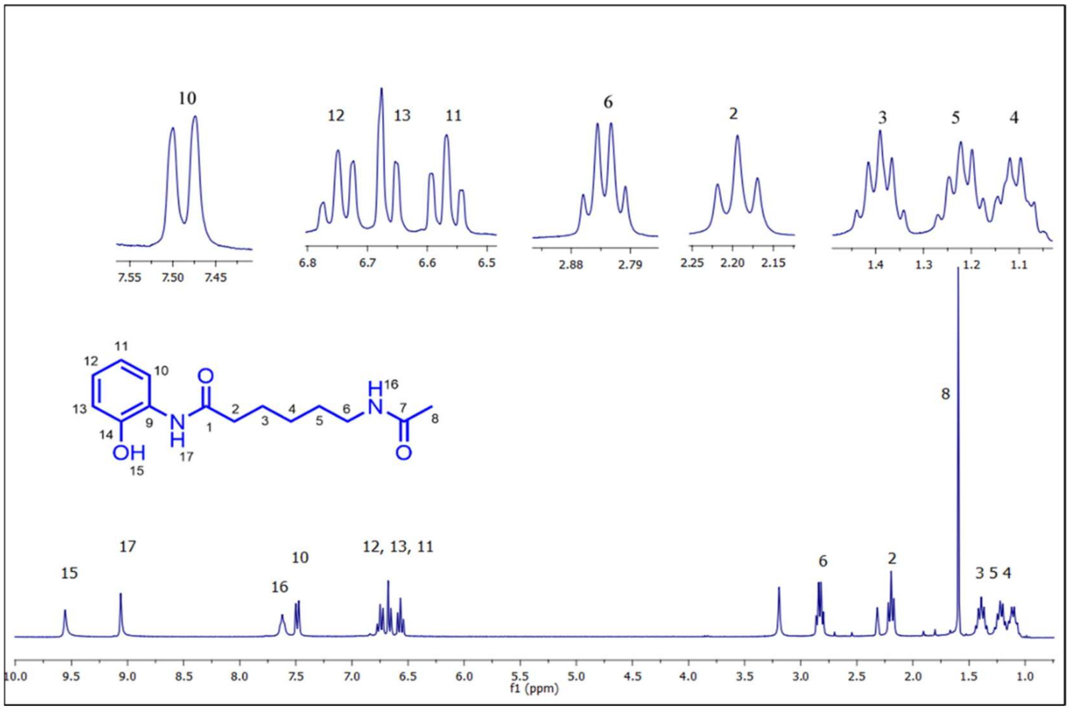

Figure S11. ^{1}H NMR spectrum, 300 MHz in DMSO-d_6 of compound **5a**.

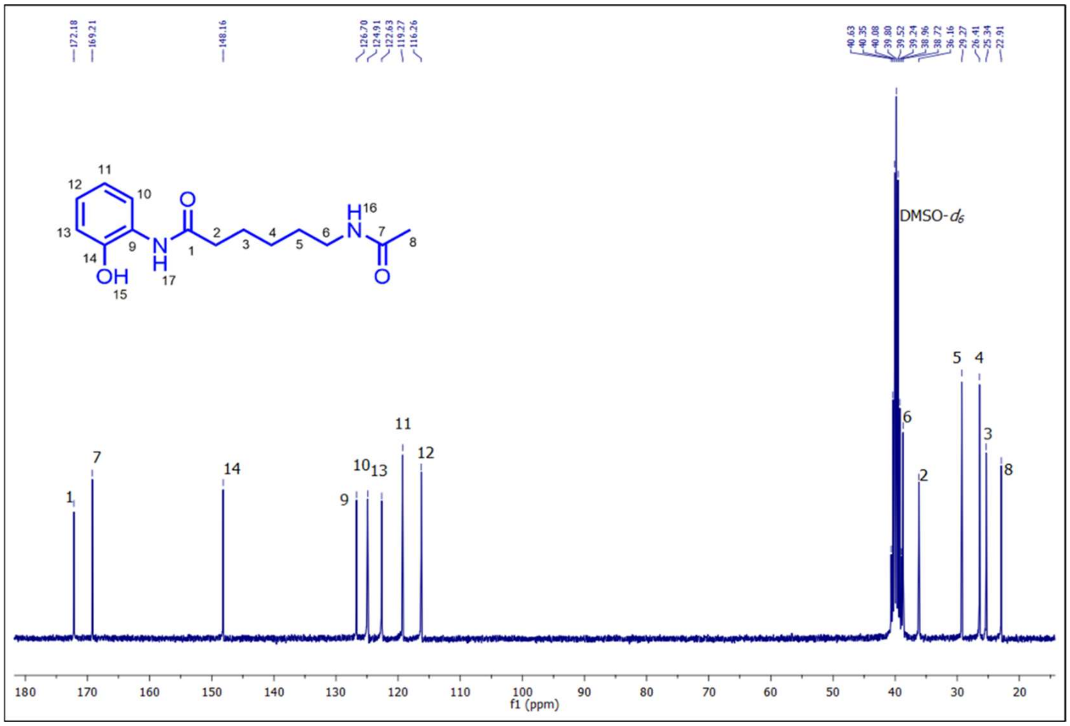

Figure S12. ^{13}C NMR spectrum, 75.5 MHz in DMSO-d_6 of compound **5a**.

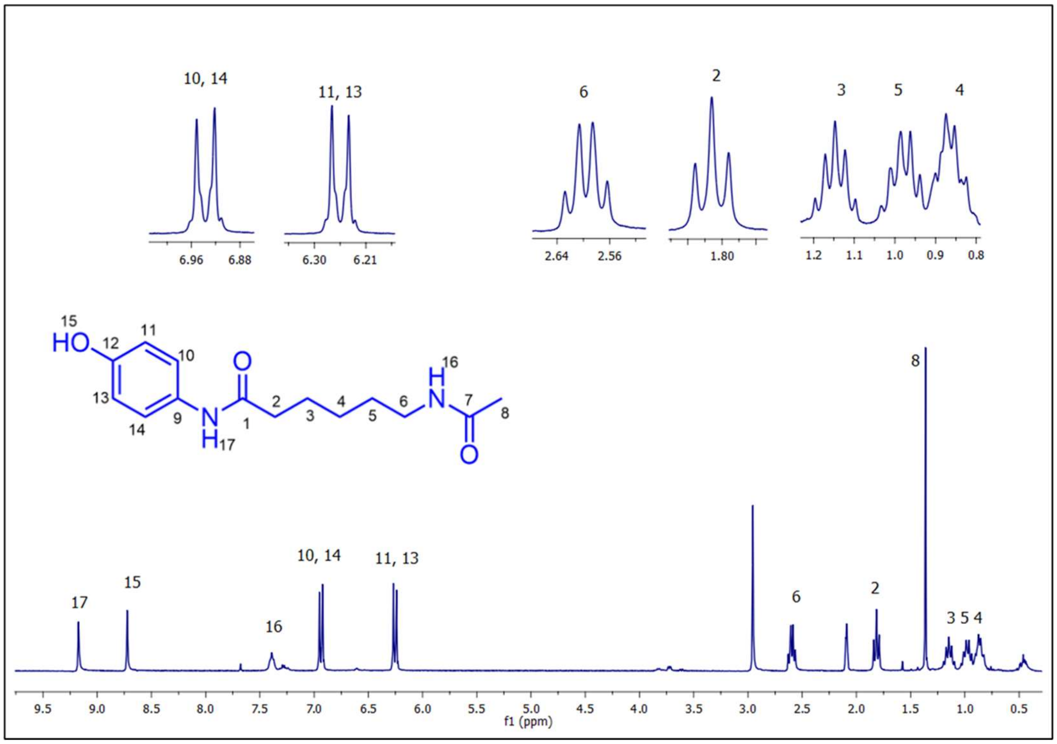

Figure S13. ^{1}H NMR spectrum, 300 MHz in DMSO-*d*6 of compound **5b**.

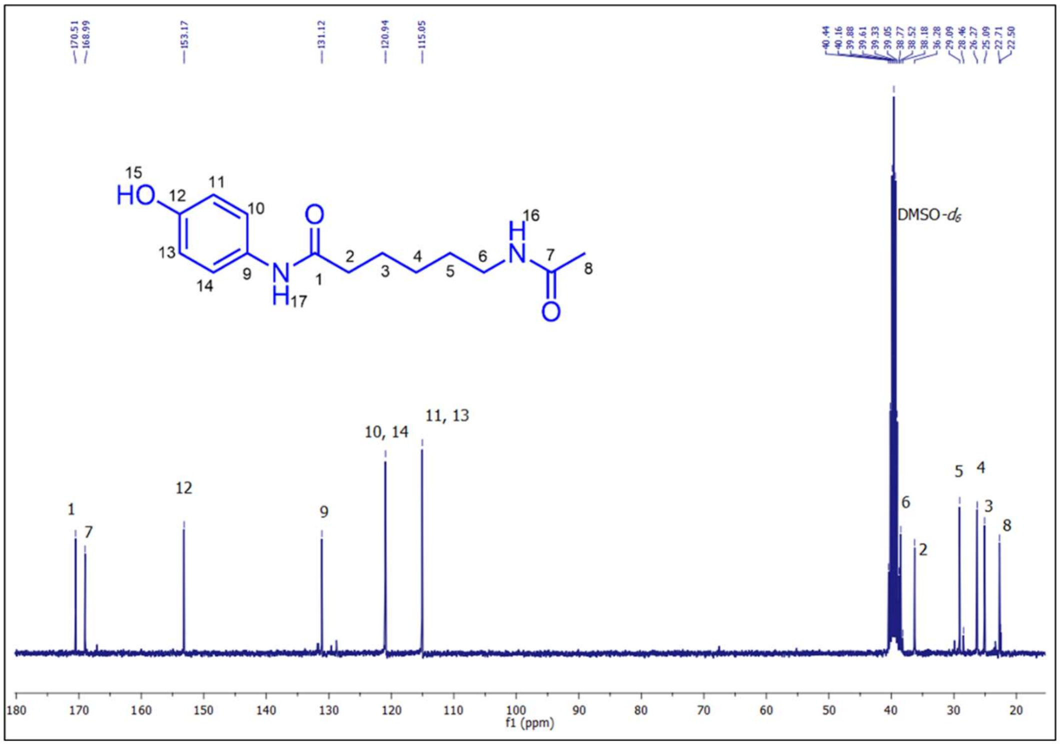

Figure S14. ^{13}C NMR spectrum, 75.5 MHz in DMSO-*d*6 of compound **5b**.

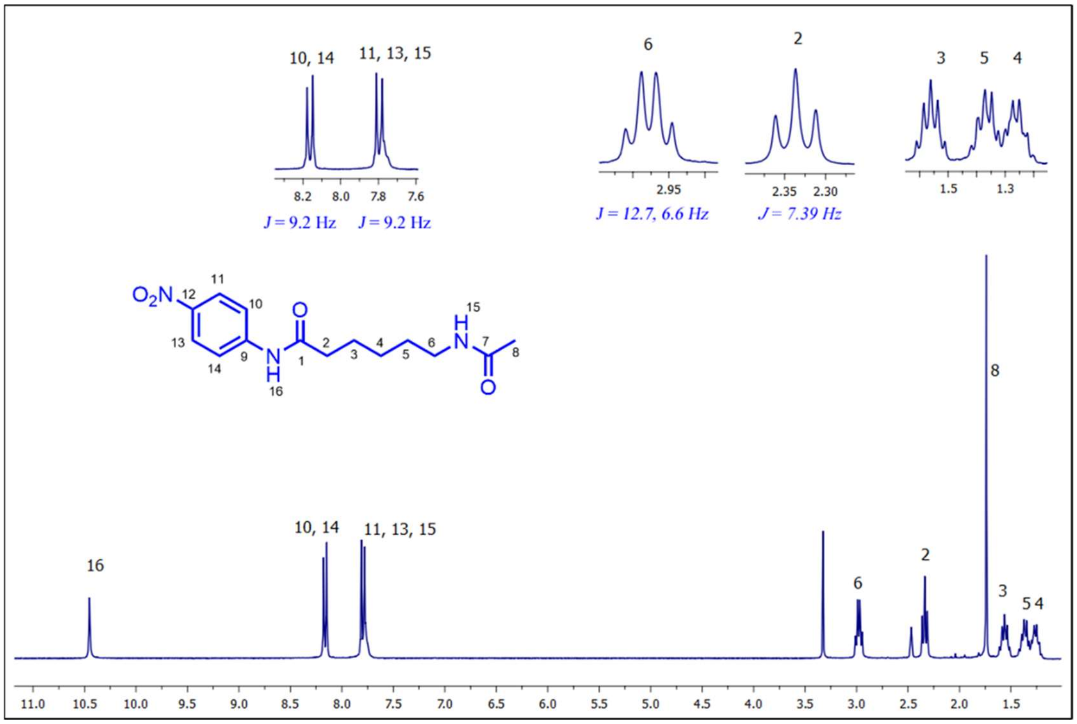

Figure S15. ^{1}H NMR spectrum, 300 MHz in DMSO-d_6 of compound **5c**.

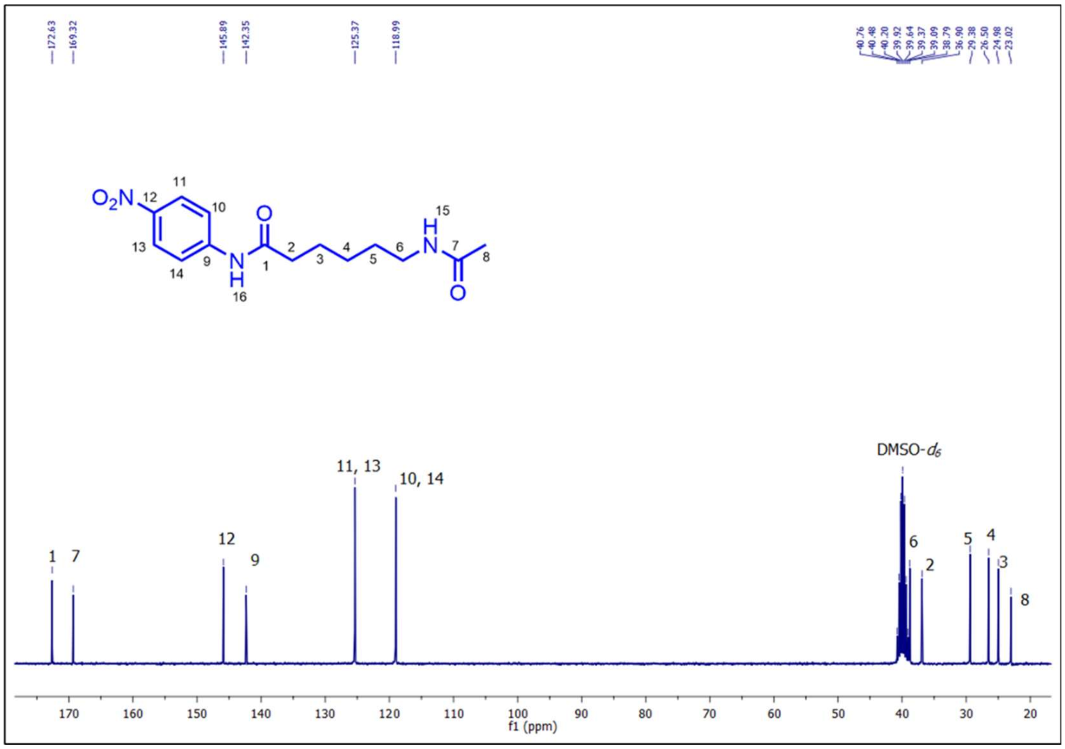

Figure S16. ^{13}C NMR spectrum, 75.5 MHz in DMSO-d_6 of compound **5c**.

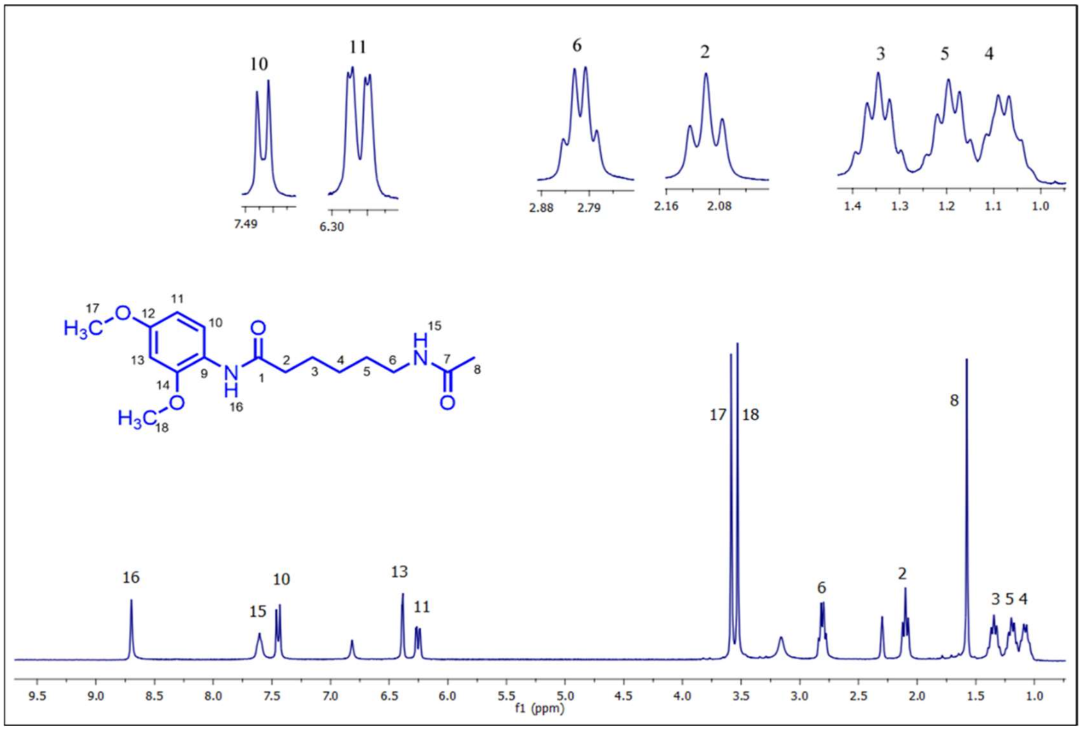

Figure S17. ^{1}H NMR spectrum, 300 MHz in DMSO-d_6 of compound **5d**.

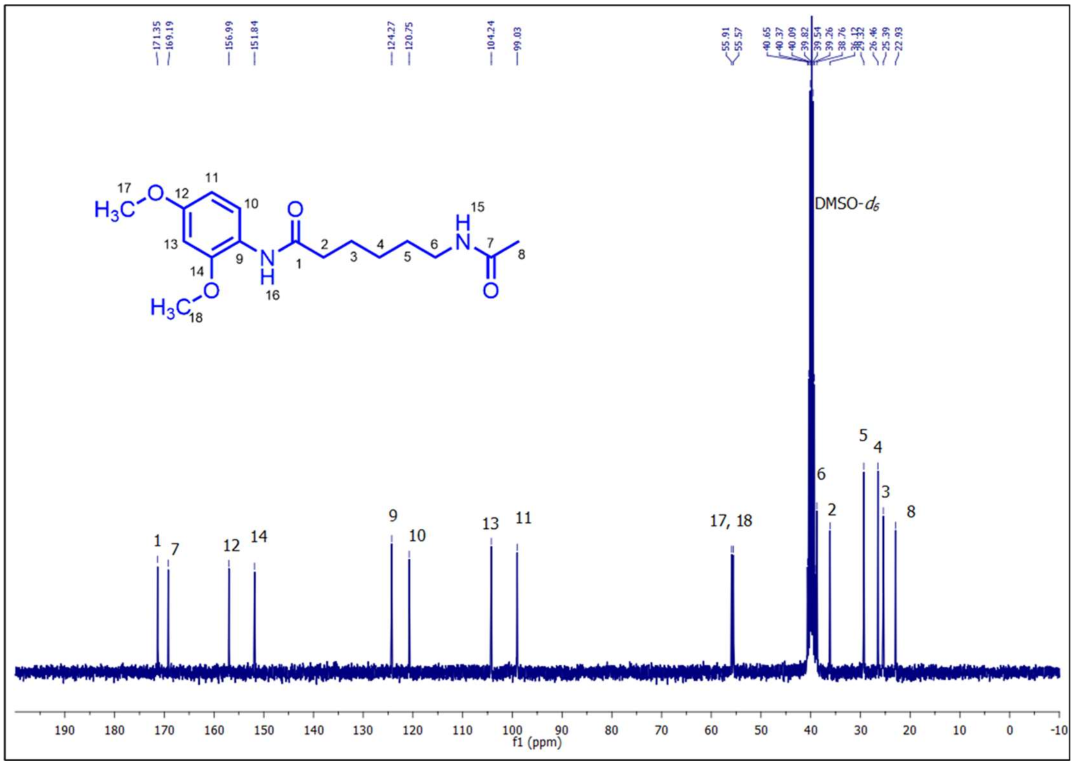

Figure S18. ^{13}C NMR spectrum, 75.5 MHz in DMSO-d_6 of compound **5d**.

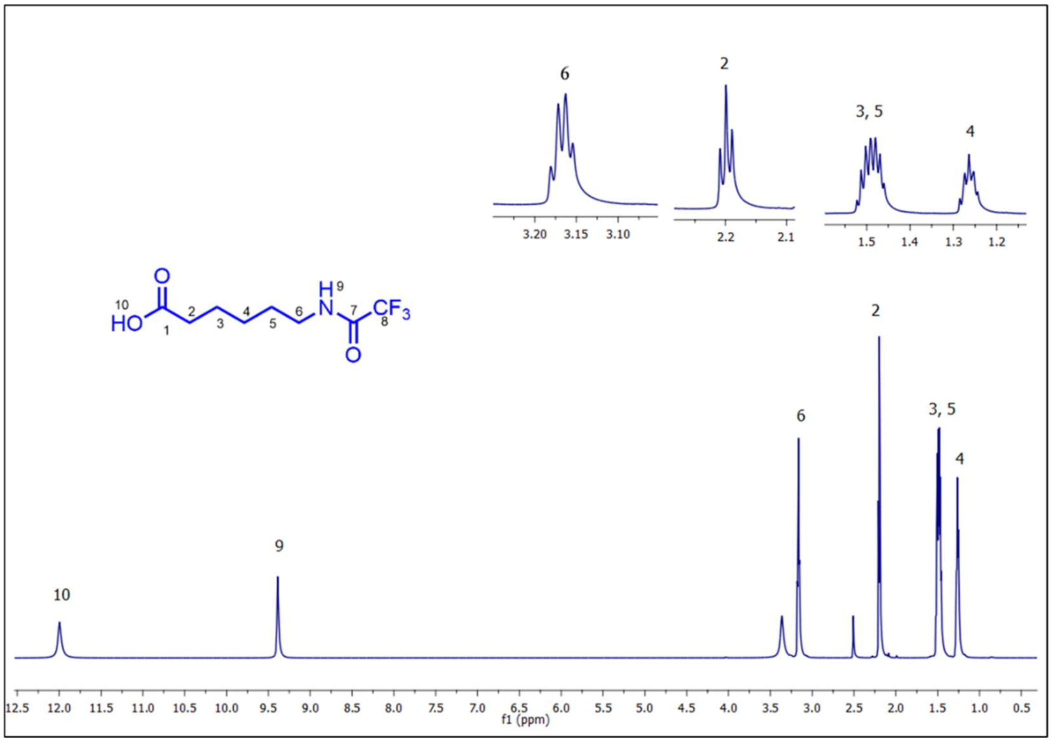

Figure S19. ^{1}H NMR spectrum, 300 MHz in DMSO-d_6 of compound **3**.

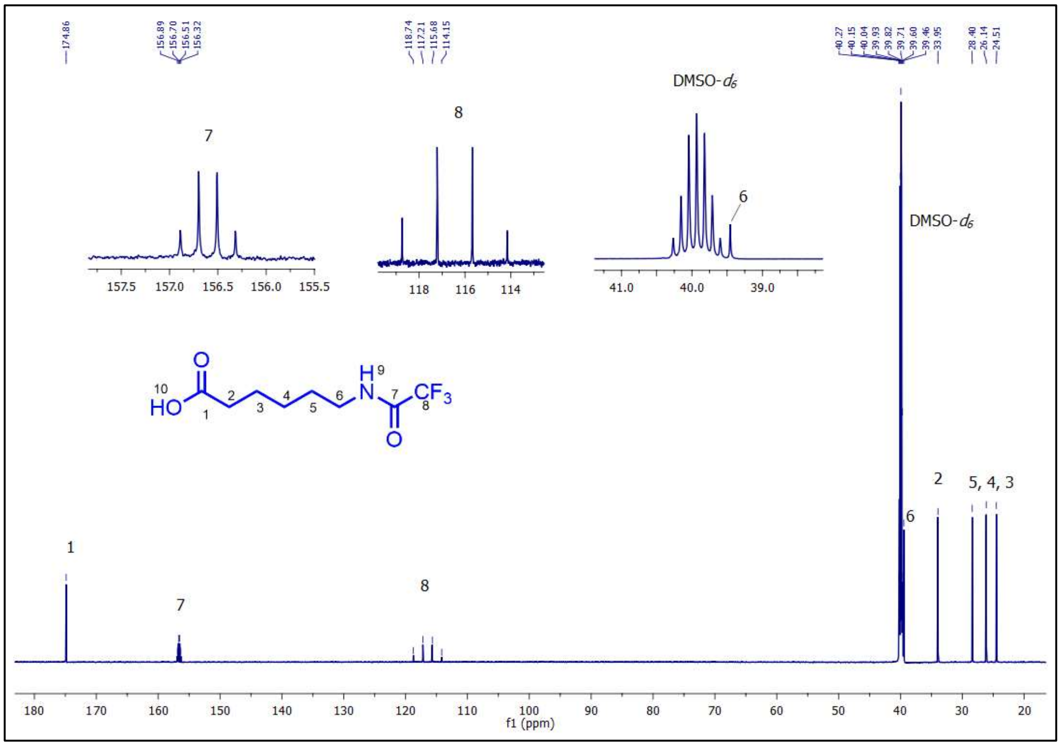

Figure S20. ^{13}C NMR spectrum, 75.5 MHz in DMSO-d_6 of compound **3**.

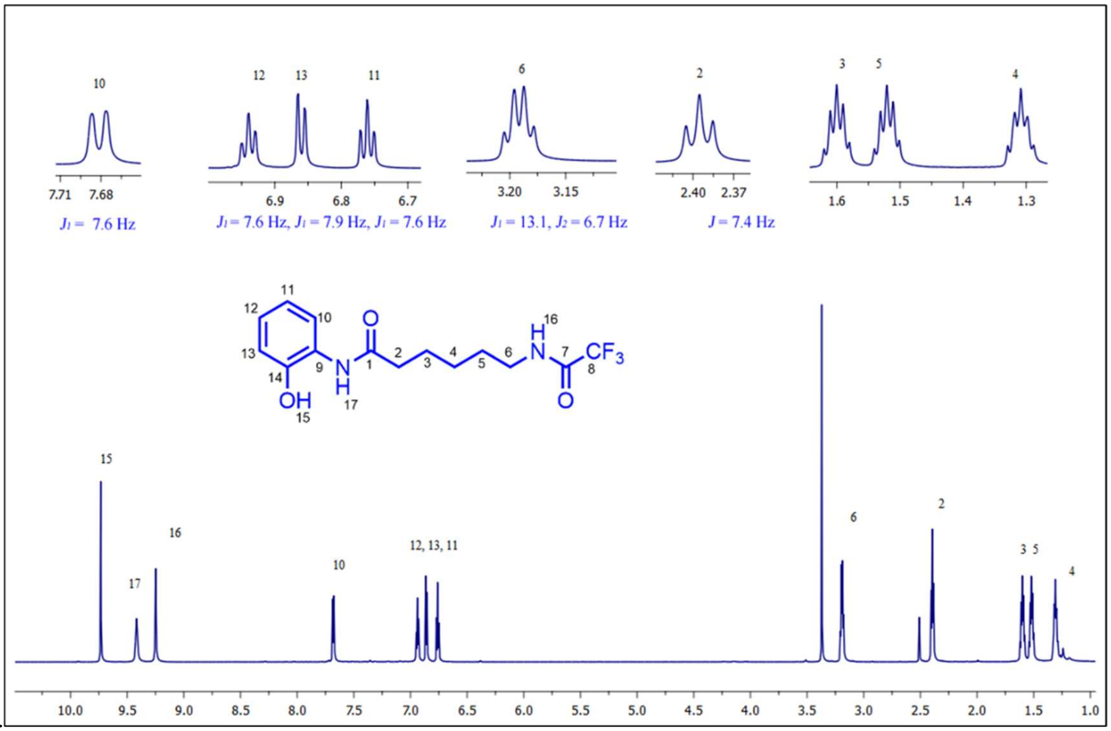

Figure S21. ^{1}H NMR spectrum, 300 MHz in DMSO-d_6 of compound **6a**.

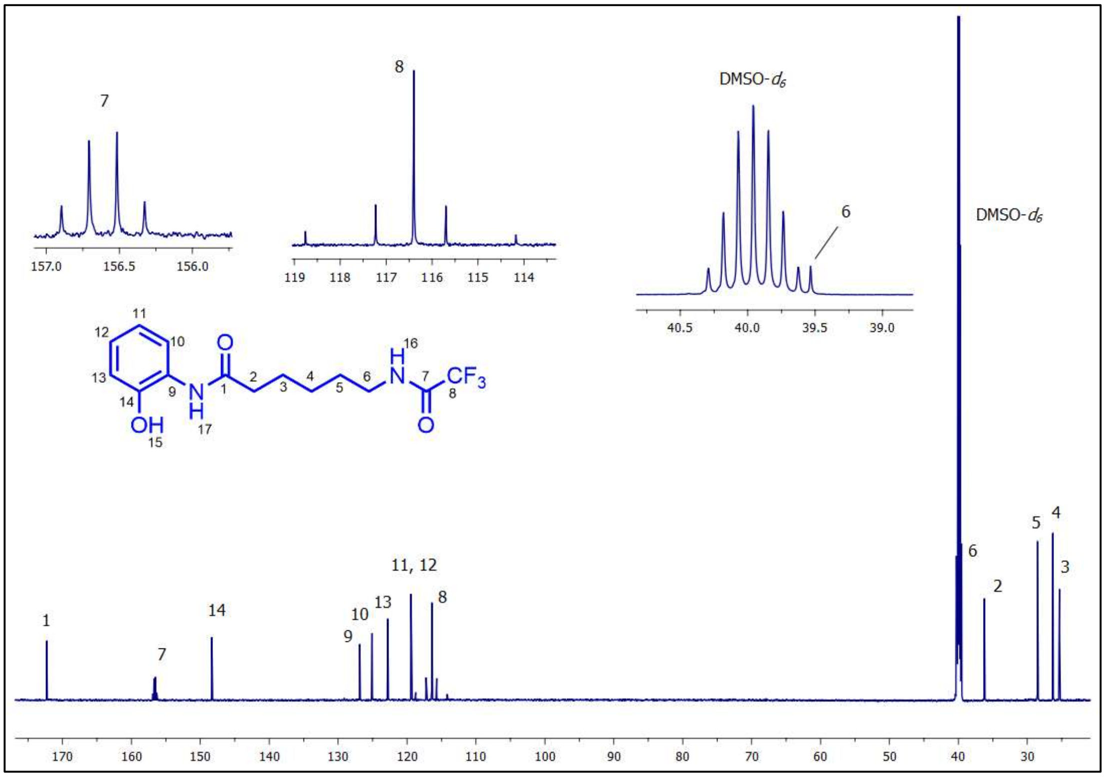

Figure S22. ^{13}C NMR spectrum, 75.5 MHz in DMSO-d_6 of compound **6a**.

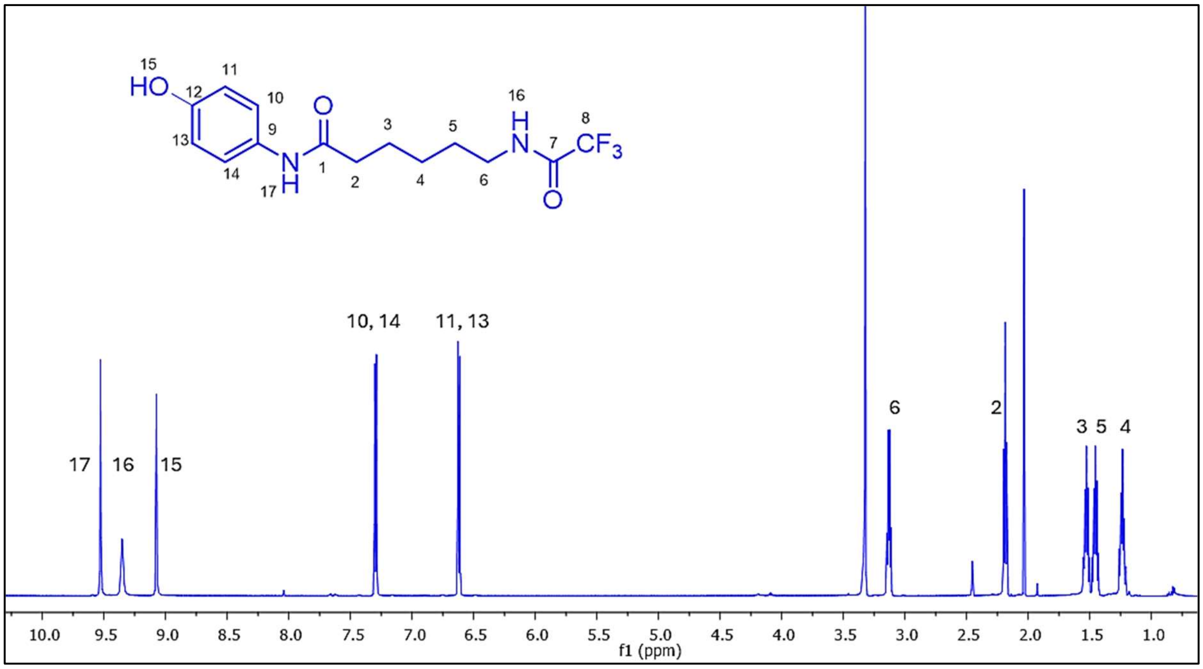

Figure S23. ^{1}H NMR spectrum, 300 MHz in DMSO-d_6 of compound **6b.**

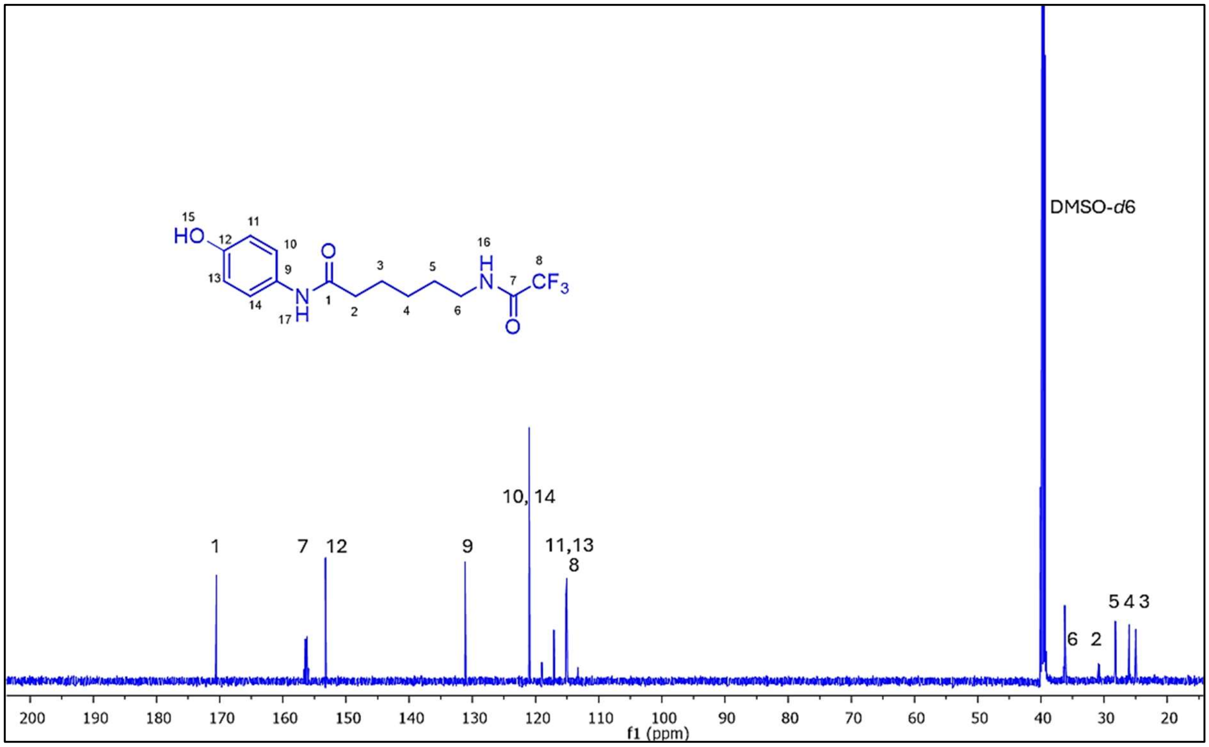

Figure S24. ^{13}C NMR spectrum, 75.5 MHz in DMSO-d_6 of compound **6b**.

High resolution mass spectrometry

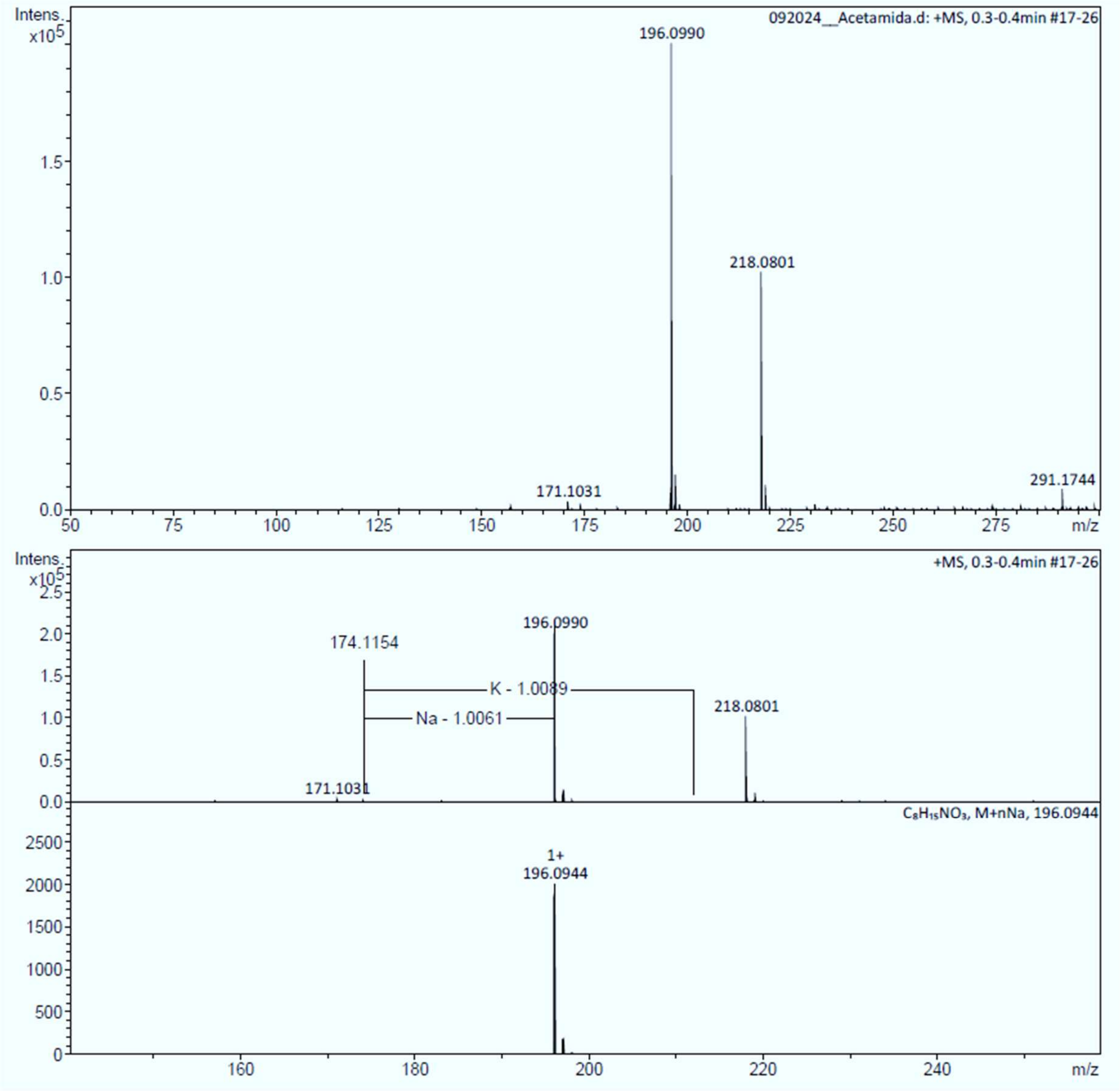

Figure S25. High resolution mass spectrometry of **2**.

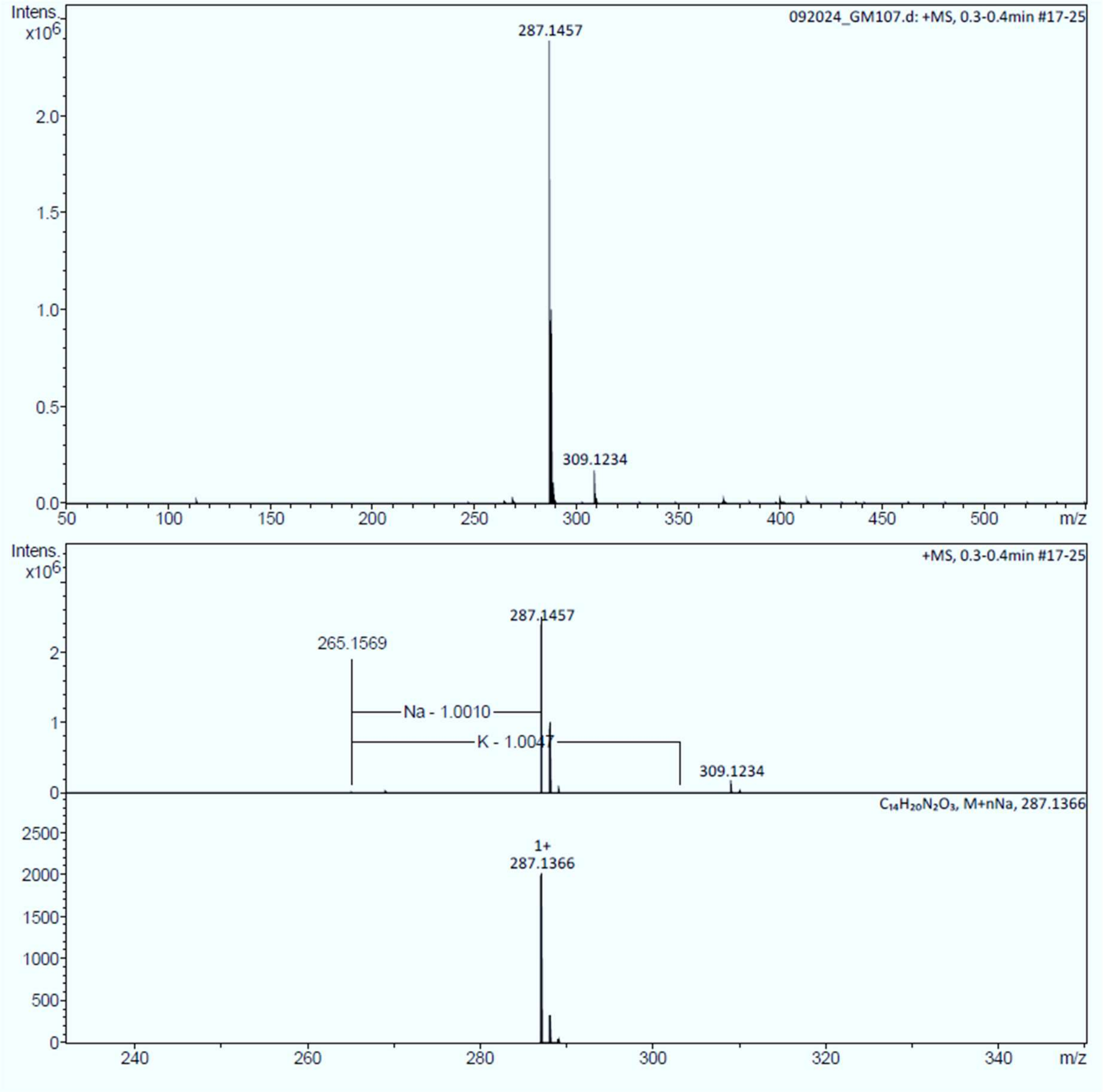

Figure S26. High resolution mass spectrometry of **5a**.

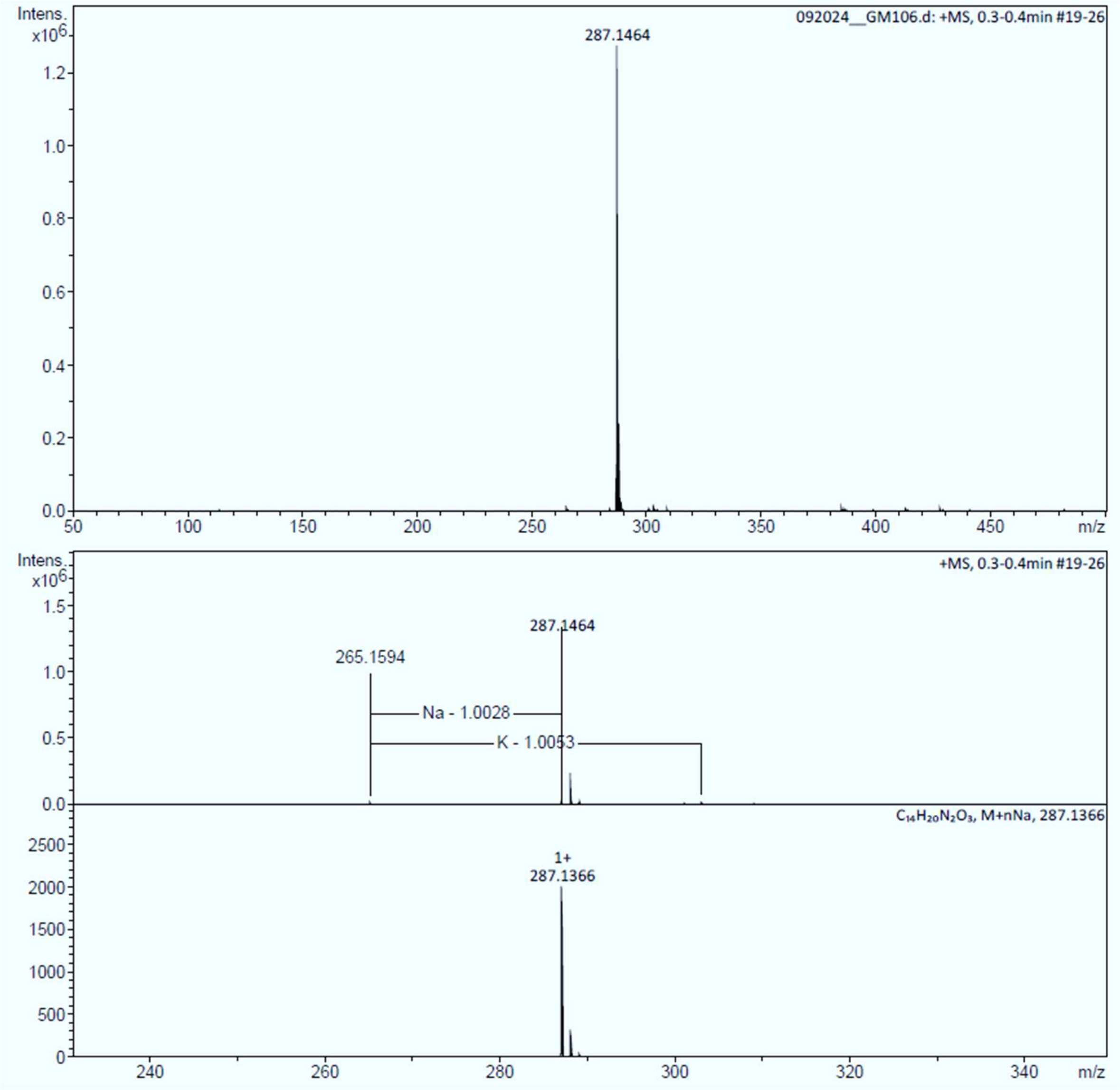

Figure S27. High resolution mass spectrometry of **5b**.

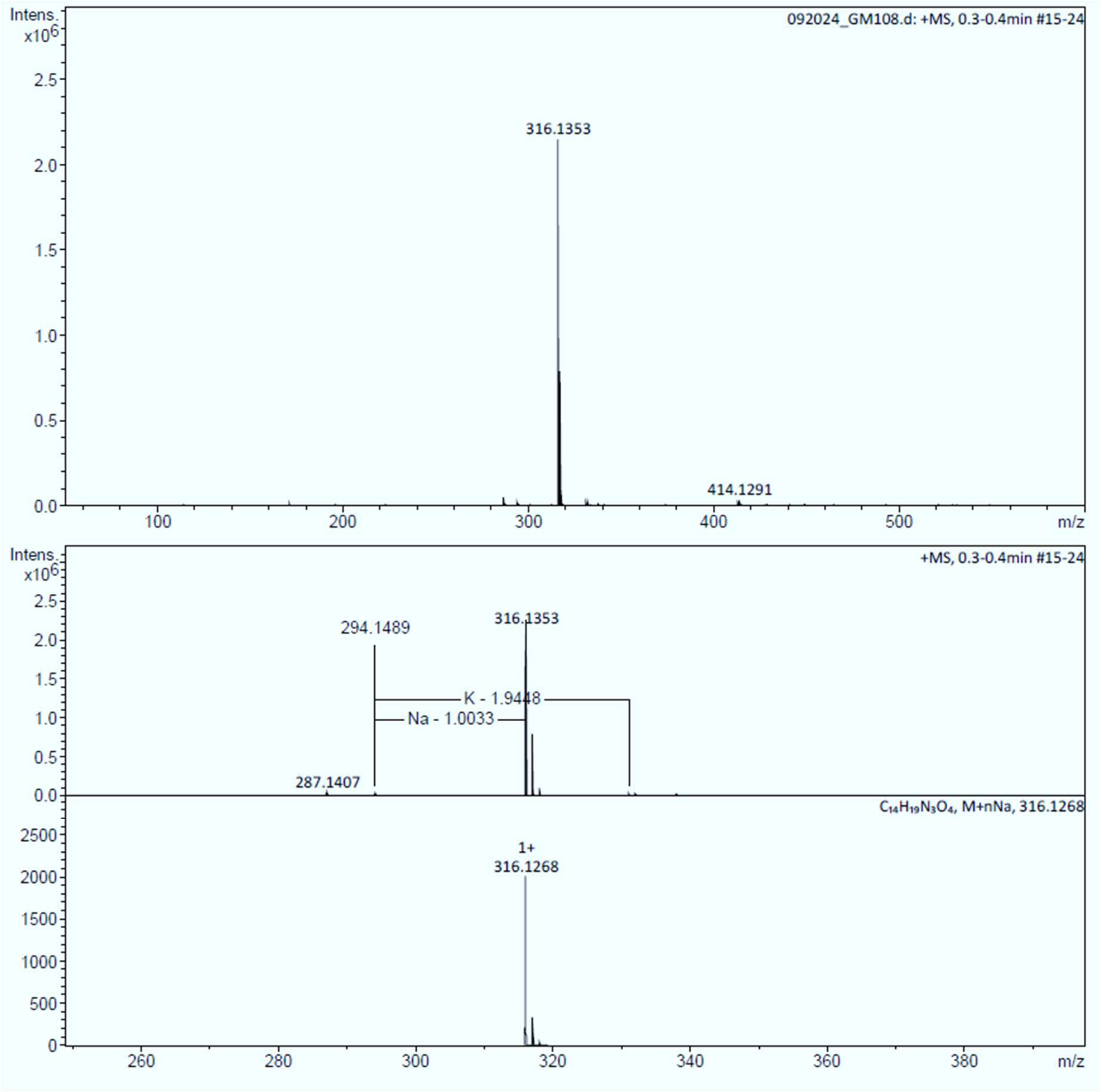

Figure S28. High resolution mass spectrometry of **5c**.

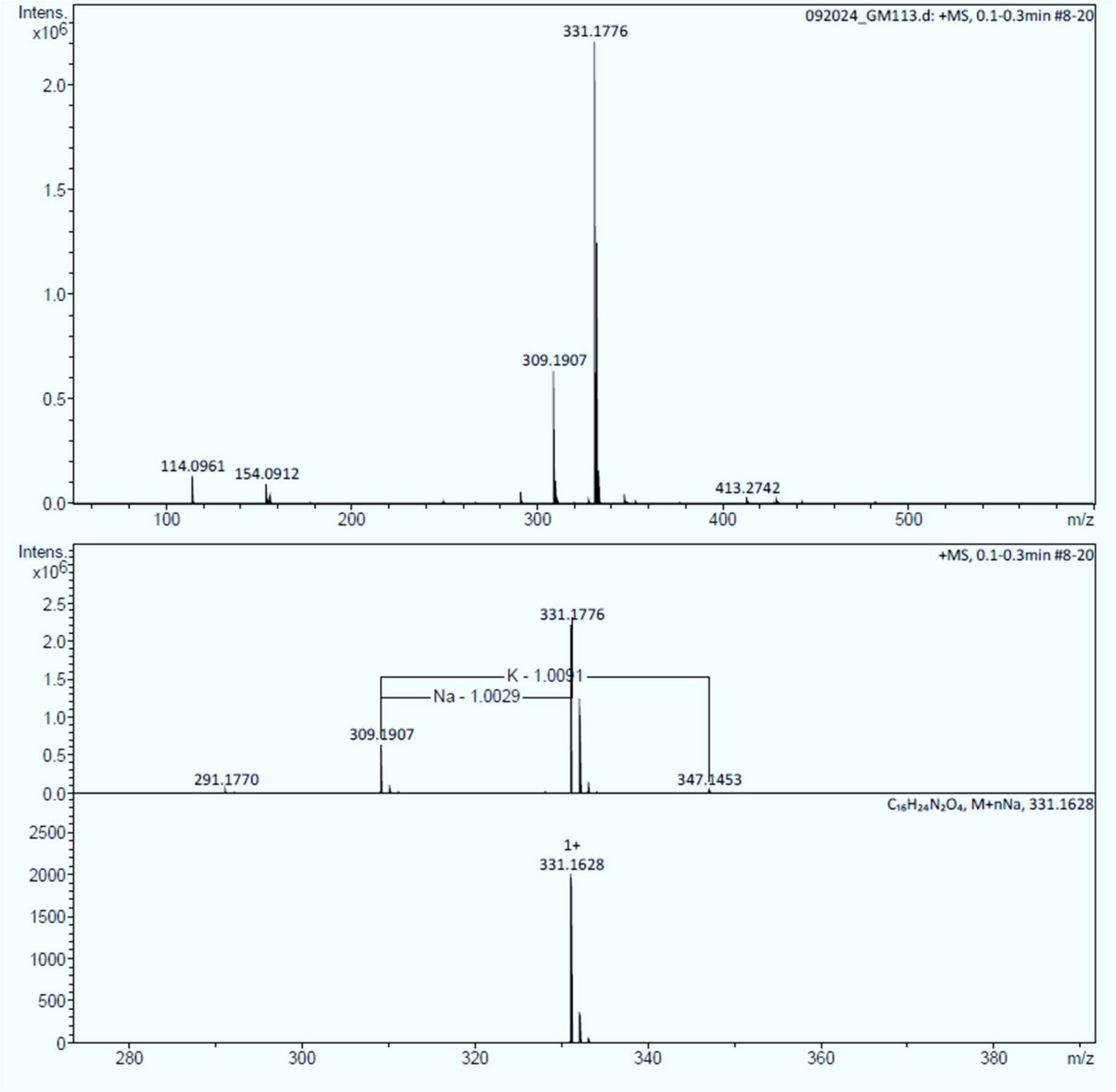

Figure S29. High resolution mass spectrometry of **5d**.

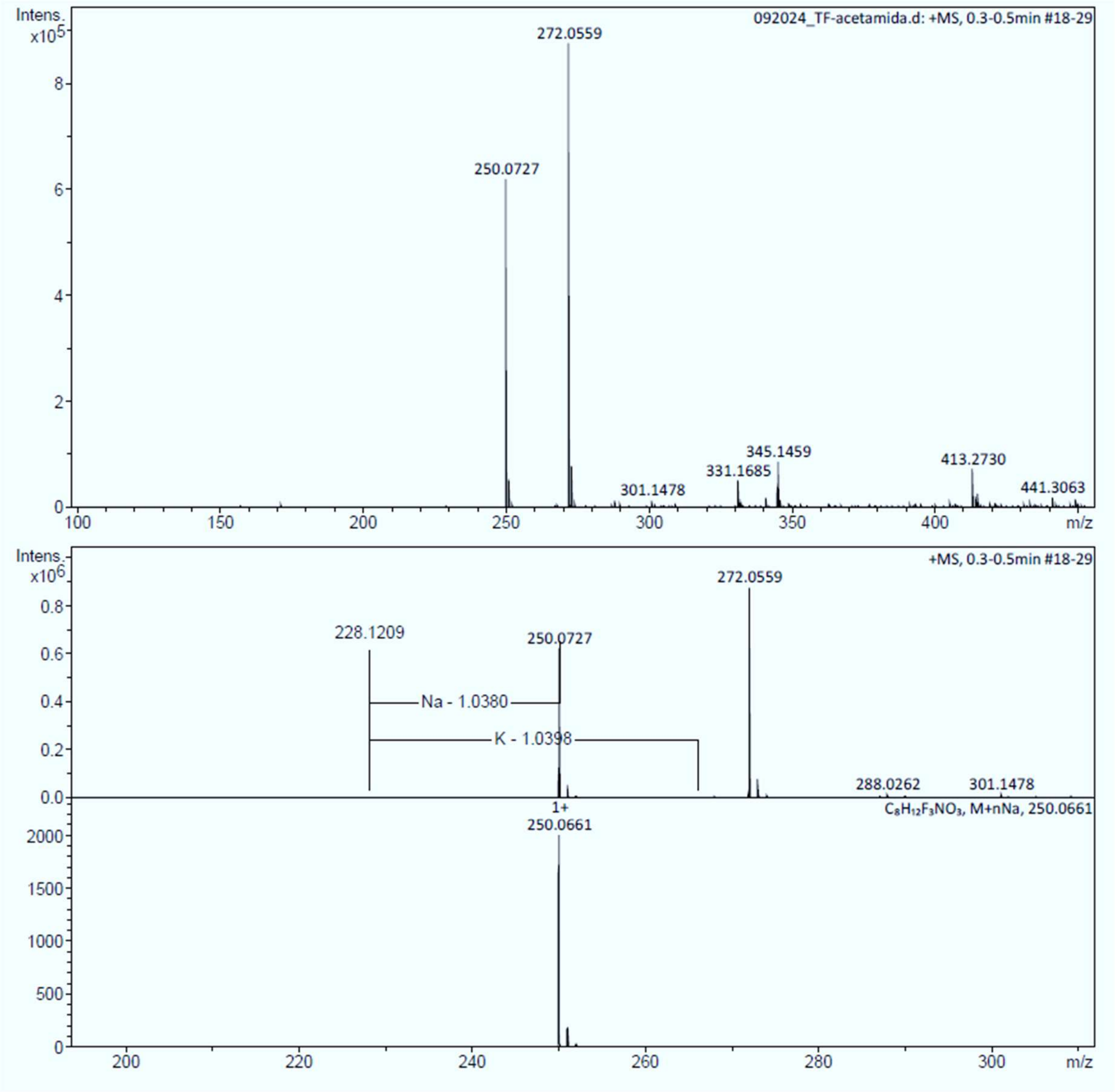

Figure S30. High resolution mass spectrometry of **3**.

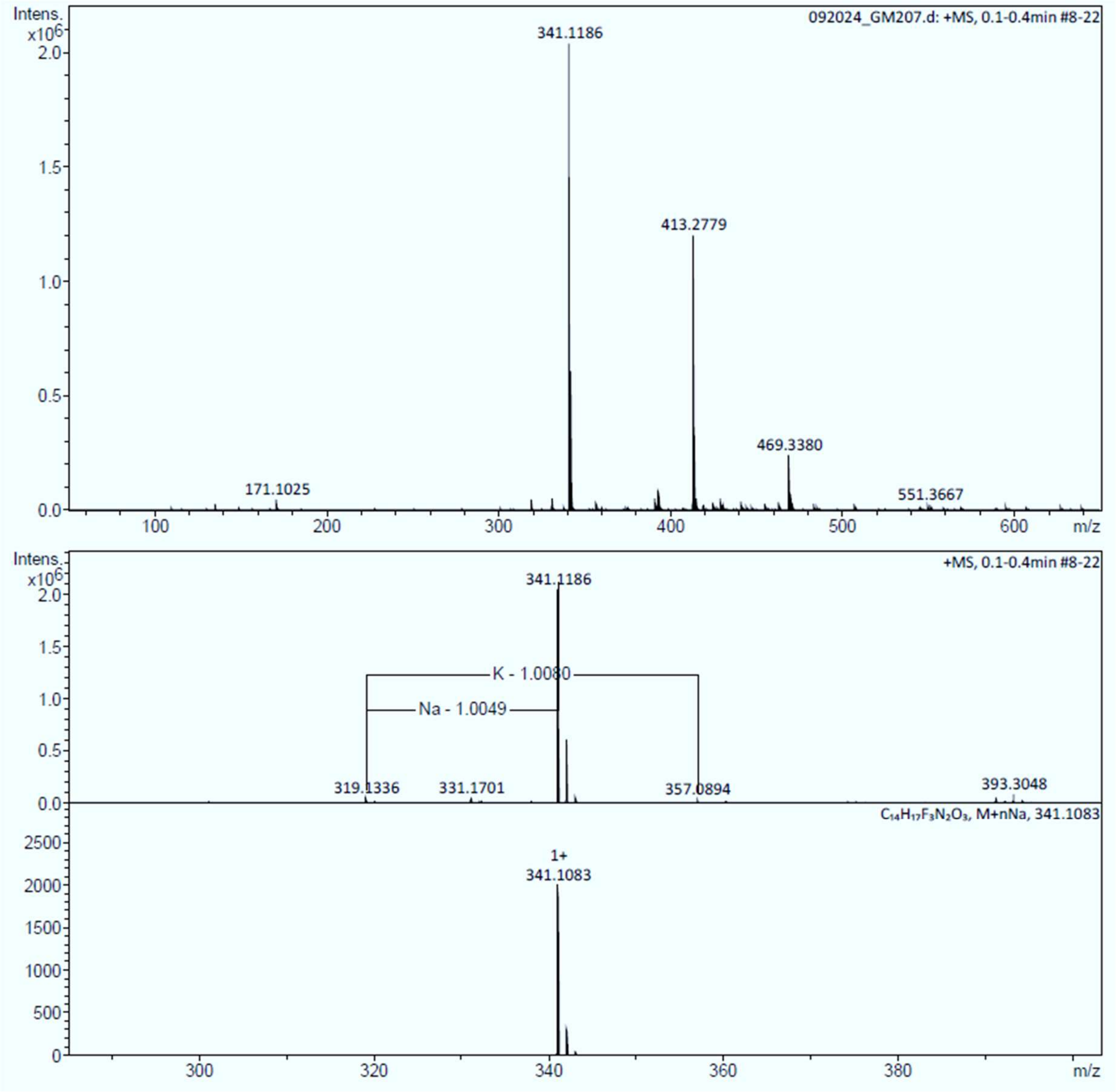

Figure S31. High resolution mass spectrometry of **6a**.

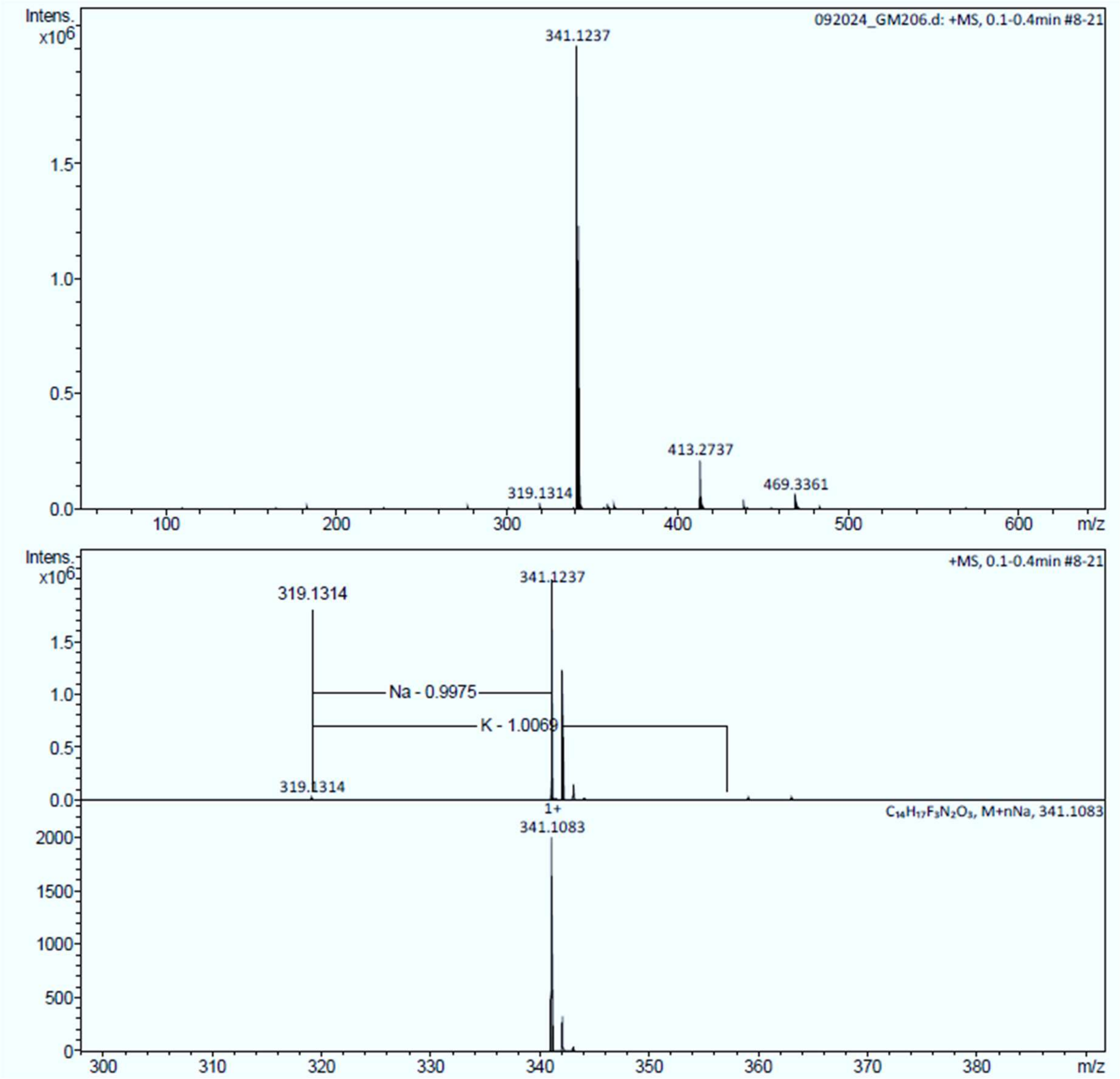

Figure S32. High resolution mass spectrometry of **6b**.

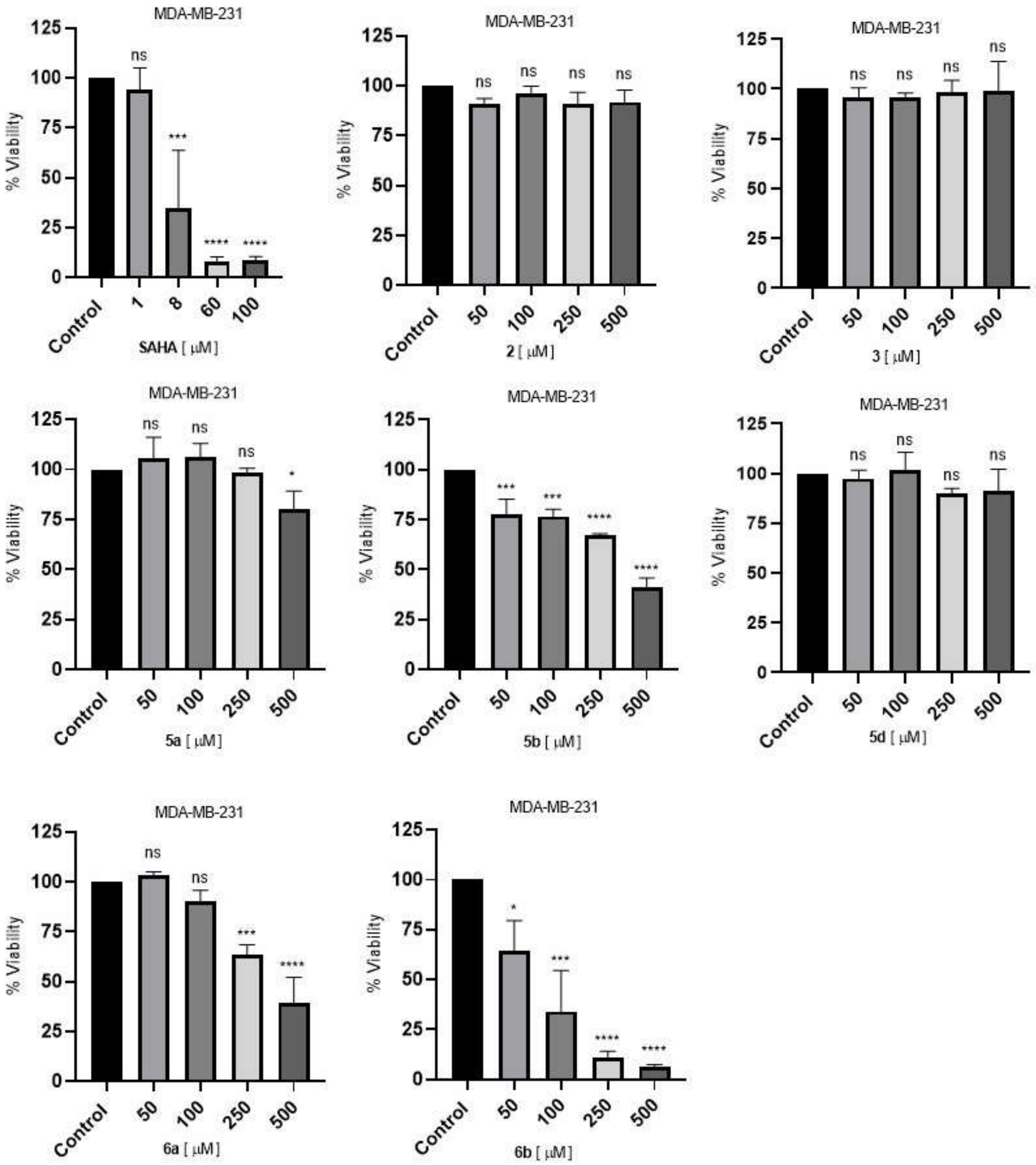

Figure S33. Initial viability experiment in MDA-MB-231 cells treated with (a) **SAHA** at 1, 8, 60 and 100 µM and (b) **2**, (c) **3**, (d) **5a**, (d) **5b**, (e) **5d**, (f) **6a**, and (g) **6b** at 50, 100, 200, and 500 µM by MTT assay. Experiments were performed in triplicate with n = 6 for each concentration. Data are presented as means ± SE. *p < 0.05 obtained by one-way ANOVA and Dunnet's as a post hoc test.

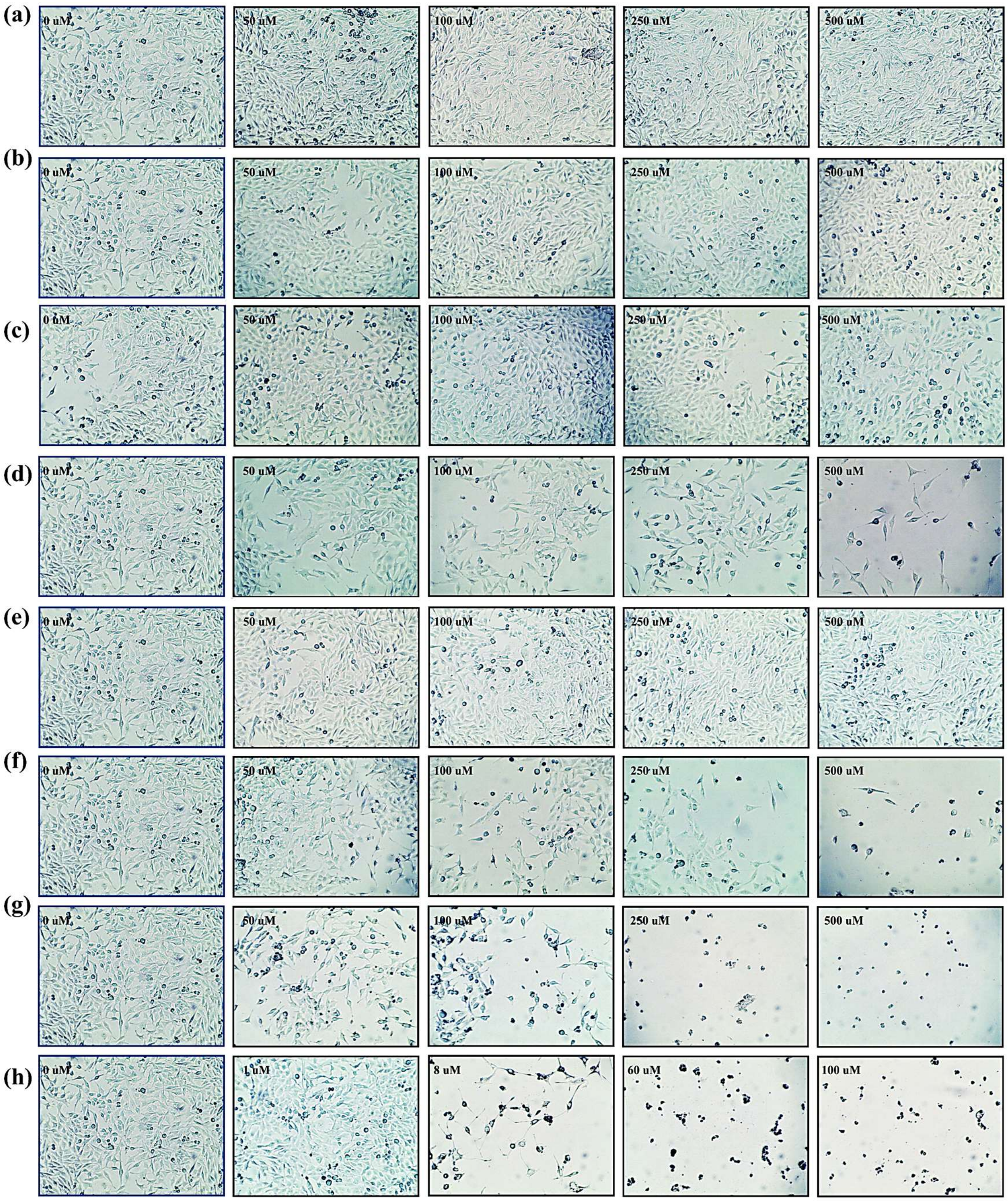

Figure S34. Optical microscopy (10x) of MDA-MB-231 cancer cells at different concentrations of (a) **2,** (b) **3,** (c) **5a,** (d) **5b,** (e) **5d,** (f) **6a,** (g) **6b** and (h) **SAHA** (Control is surrounded in blue color).

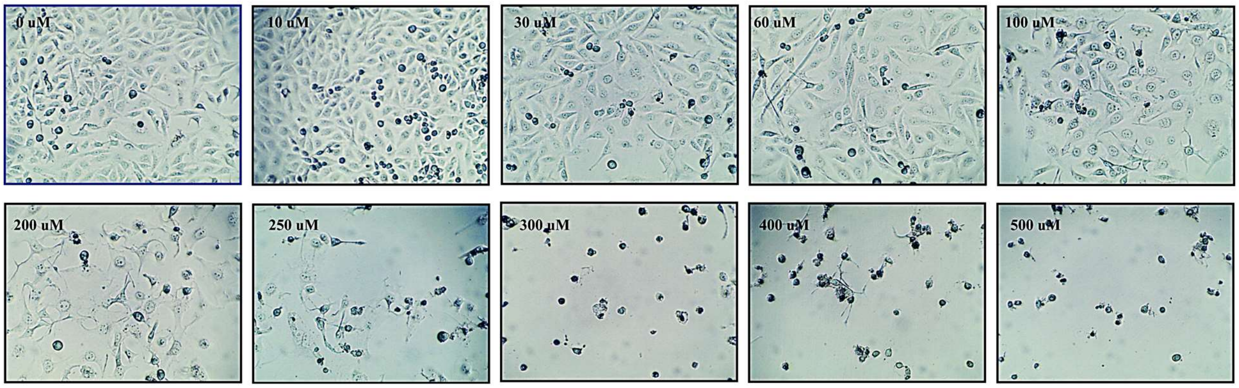

Figure S35. Optical microscopy (10x) of MDA-MB-231 cancer cells at different concentrations of **5b** (Control is surrounded in blue color).

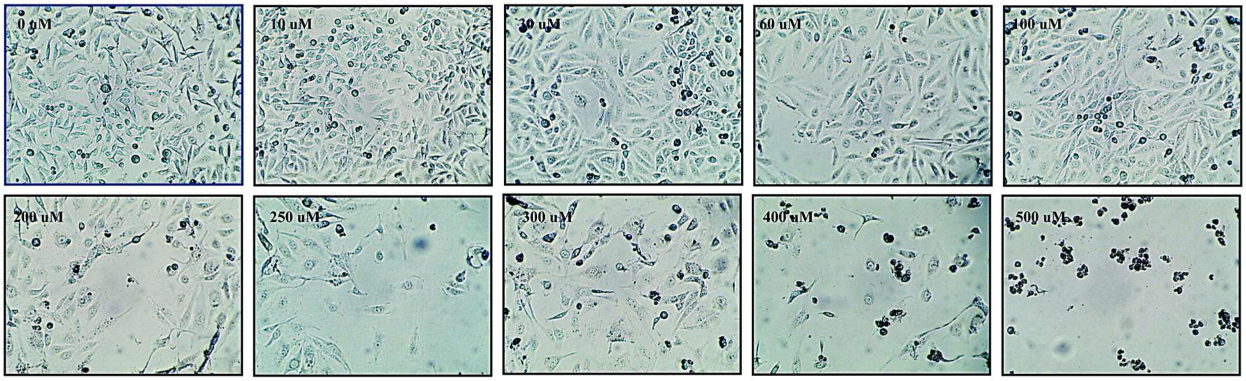

Figure S36. Optical microscopy (10x) of MDA-MB-231 cancer cells at different concentrations of **6a** (Control is surrounded in blue color).

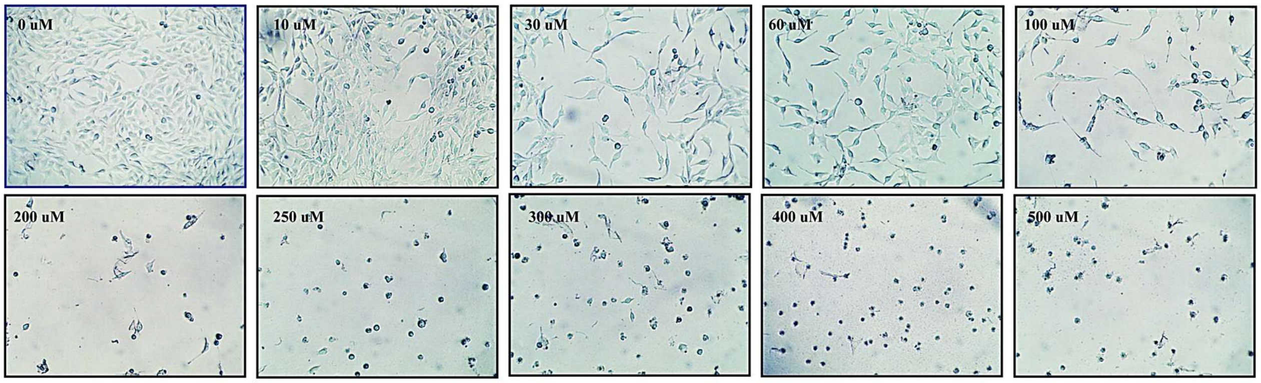

Figure S37. Optical microscopy (10x) of MDA-MB-231 cancer cells at different concentrations of **6b** (Control is surrounded in blue color).

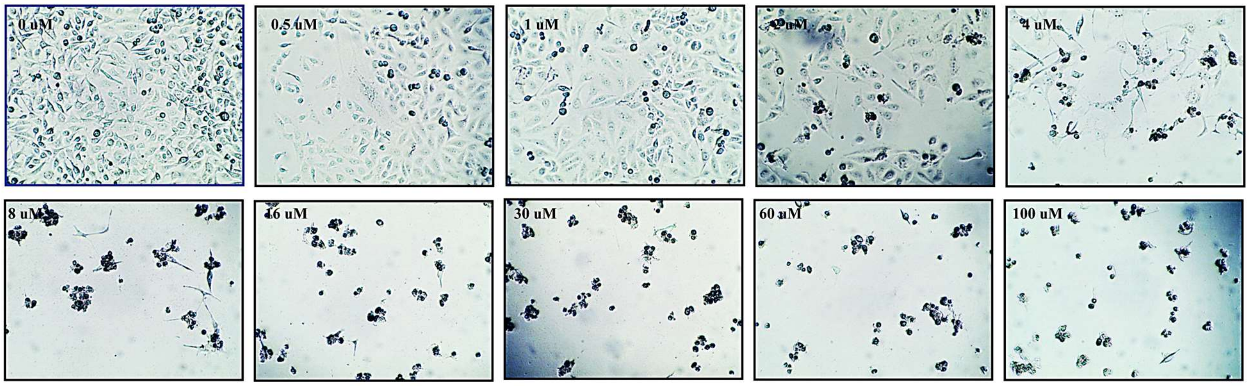

Figure S38. Optical microscopy (10x) of MDA-MB-231 cancer cells at different concentrations of **SAHA** (Control is surrounded in blue color).

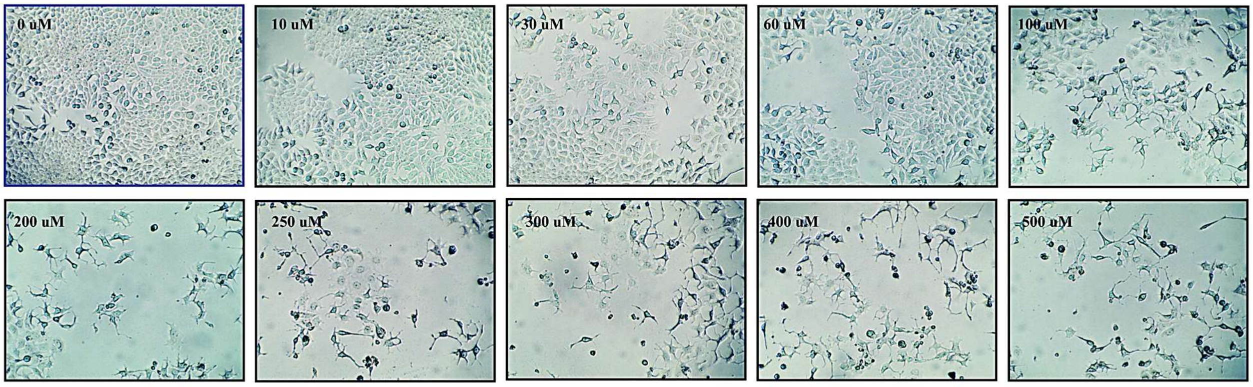

Figure S39. Optical microscopy (10x) of MCF-7 cancer cells at different concentrations of **5b** (Control is surrounded in blue color).

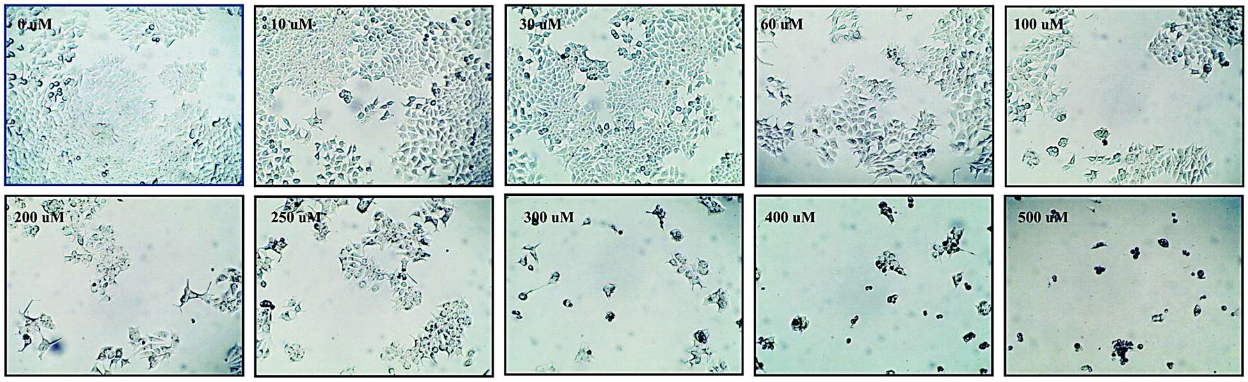

Figure S40. Optical microscopy (10x) of MCF-7 cancer cells at different concentrations of **6a** (control is surrounded in blue color)

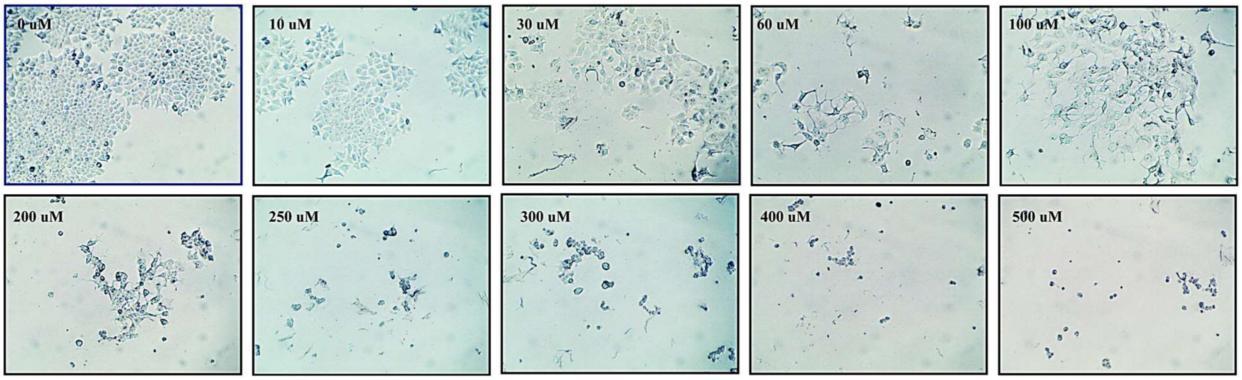

Figure S41. Optical microscopy (10x) of MCF-7 cancer cells at different concentrations of **6b** (Control is surrounded in blue color).

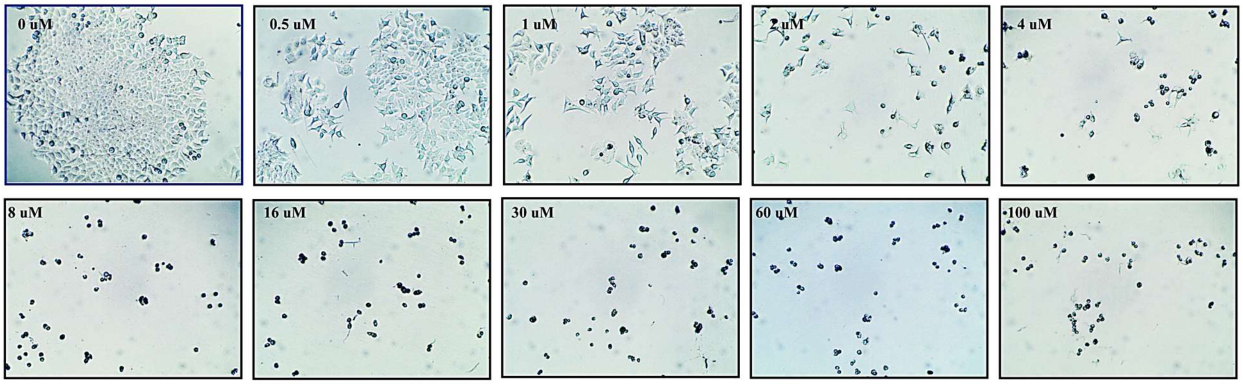

Figure S42. Optical microscopy (10x) of MCF-7 cancer cells at different concentrations of **SAHA** (Control is surrounded in blue color).

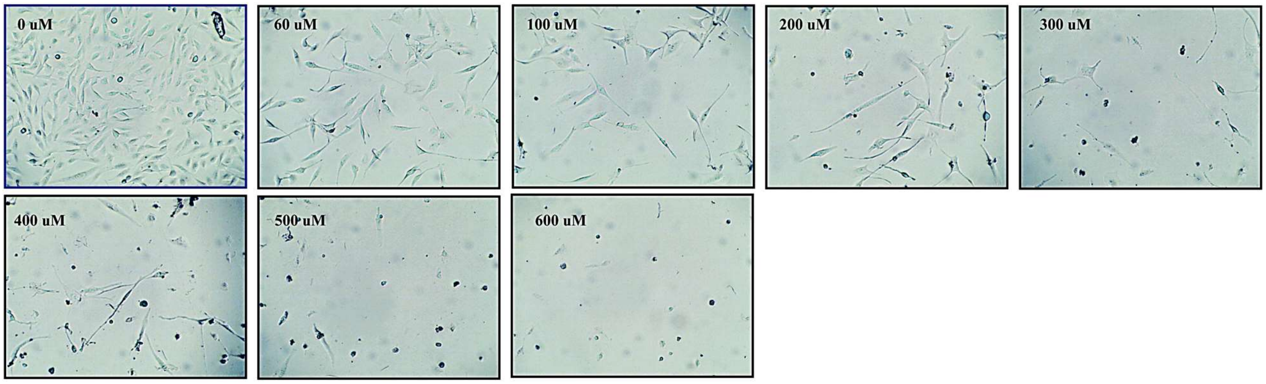

Figure S43. Optical microscopy (10x) of MCF-10A cells at different concentrations of **5b** (Control is surrounded in blue color).

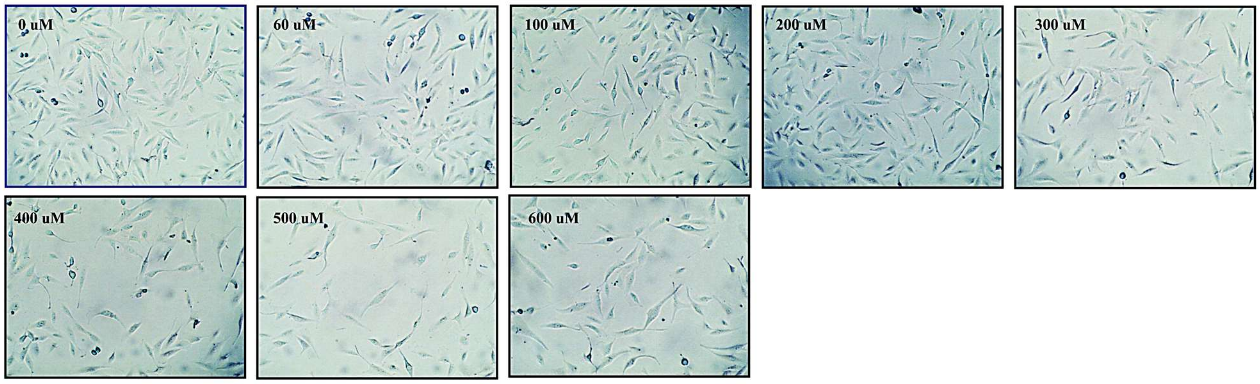

Figure S44. Optical microscopy (10x) of MCF-10A cells at different concentrations of **6a** (Control is surrounded in blue color).

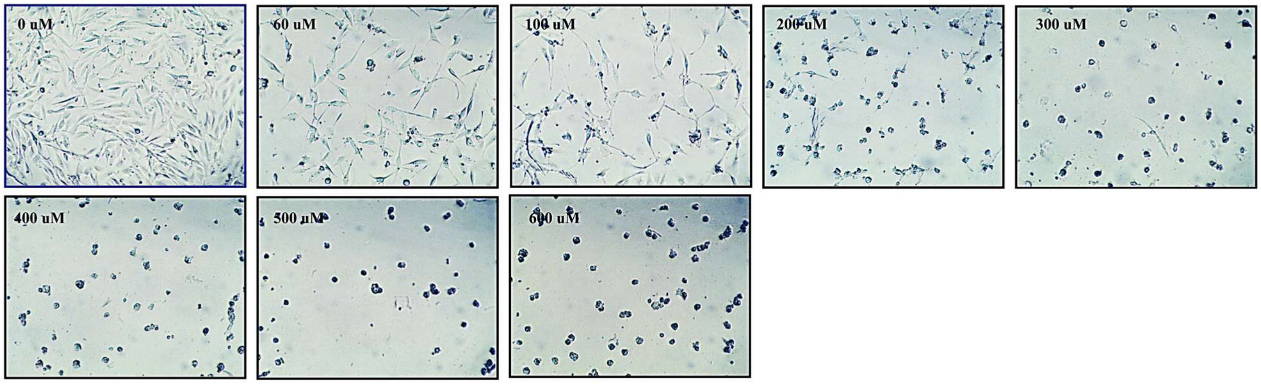

Figure S45. Optical microscopy (10x) of MCF-10A cells at different concentrations of **6b** (Control is surrounded in blue color).

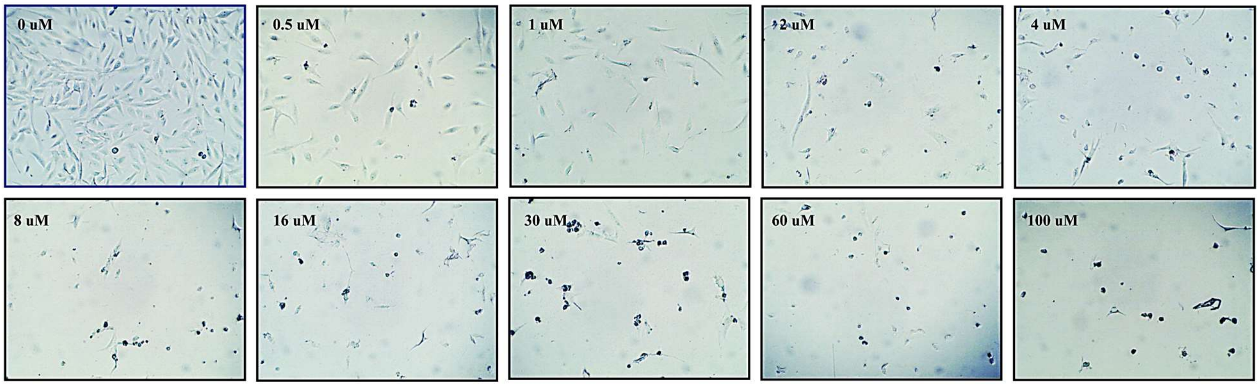

Figure S46. Optical microscopy (10x) of MCF-10A cells at different concentrations of **SAHA** (Control is surrounded in blue color).
